# Moving from Measuring, Reporting, Verification (MRV) of Forest Carbon to Community Mapping, Measuring, Monitoring (MMM): Perspectives from Mexico

**DOI:** 10.1371/journal.pone.0146038

**Published:** 2016-06-14

**Authors:** Michael K. McCall, Noah Chutz, Margaret Skutsch

**Affiliations:** 1 CIGA, UNAM, Morelia, Michoacán, Mexico; 2 Independent Consultant, Silverton, CO, United States of America; CIFOR Headquarters, INDONESIA

## Abstract

There have been many calls for community participation in MRV (measuring, reporting, verification) for REDD+. This paper examines whether community involvement in MRV is a requirement, why it appears desirable to REDD+ agencies and external actors, and under what conditions communities might be interested in participating. It asks *What’s in it for communities*? *What might communities gain from such an involvement*? *What could they lose*? It embraces a broader approach which we call community MMM which involves mapping, measuring and monitoring of forest and other natural resources for issues which are of interest to the community itself. We focus on cases in México because the country has an unusually high proportion of forests under community communal ownership. In particular, we refer to a recent REDD+ initiative—CONAFOR-LAIF, in which local communities select and approve local people to participate in community-based monitoring activities. From these local initiatives we identify the specific and the general drivers for communities to be involved in mapping, measuring and monitoring of their own territories and their natural resources. We present evidence that communities are more interested in this wider approach than in a narrow focus on carbon monitoring. Finally we review what the challenges to reconciling MMM with MRV requirements are likely to be.

## 1. Is community monitoring a requirement for MRV for REDD+?

In REDD+, there are five components which may be compensated for at the national level, and whose performance therefore, would need to be measured for this level: i) reducing emissions from deforestation; ii) reducing emissions from degradation; iii) conservation for forest carbon stocks; iv) enhanced forest carbon stocks; and, v) sustainable management of forests. Measurement is in terms of changes in carbon stocks over time, and should take into account any leakage. In their calculations, most countries rely on satellite data and IPCC (Intergovernmental Panel on Climate Change) default estimates (Tier 1) of typical standing stock levels in different forest types, as few have forest inventories which can provide comprehensive, time-series ground level data. In addition, measurements are needed for a range of safeguards which include (internal) social distribution; biodiversity; transparent and effective national forest governance structures; respect for the rights (and the knowledge) of indigenous peoples and local communities; full and effective participation of stakeholder actors; (national forestry) policy compatibility; and human rights [1, 2, 3, 4, 5].

The involvement of communities—indigenous, forest-dependent and local–in MRV was addressed in the Cancun Agreement COP16 2010 and at COP15 in Copenhagen, in Decision 4/CP 15, which stated that ‘COP encourages as appropriate, the development of guidance for effective engagement of indigenous peoples and local communities in monitoring and reporting’. This followed the earlier SBSTA30 statement (1/CP.13, paragraph 1 (b) (iii)) in Bonn 2009 that there is a “need for full and effective engagement of indigenous peoples and local communities in, and potential contribution of their *knowledge to*, *monitoring and reporting of activities* relating to. REDD+…”. This however stops short of saying that communities have to monitor; it is clearly not a requirement, but an option open to countries [2, 3, 4].

Whether monitoring at community level is useful to a country depends on the protocols deployed for setting up its national forest information system, particularly the choice of scales. Under UNFCCC-compliant REDD+, national performance will be assessed relative to an agreed national baseline. However, the country can choose to construct nested baselines with separate baselines for each state/province, or a three level system with baselines at national, state and local levels. Creating baselines for every landholding would be too expensive. The choice of whether or not to engage communities in monitoring also depends on how countries expect to distribute the compensation which they receive at national level. In-country distribution to participating communities could be based on their individual performance, clearly requiring data on performance (outputs) assessed against a local baseline for each community; however this is very difficult to implement in practice [6].

The term *community-based MRV* (measuring, reporting, verification) as often used in the context of REDD+ could in many ways be considered a contradiction in terms. MRV is not community-based; the M is driven by external needs according to externally determined parameters relating to measurement and precision, and the data are intended for national-level carbon accounting processes; whilst the R and V refer to specific processes by which the country reports its achievements to UNFCCC. We propose that *Community-based MMM* (mapping, measuring, monitoring), where the information-acquisition processes are specifically aimed towards local purposes and local users, is a more appropriate objective in terms of communities’ interests and participation—the challenge is to design and adapt it to also fulfil much of the ‘measuring’ and ‘reporting’ required in MRV.

## 2. Methods

The initial methodology employed is the review of literature on community participation principles and experiences in, not just REDD+ forest carbon projects, but, natural resource management in general. There is considerable research in community involvement in biodiversity monitoring and citizen science overall [7, 8, 9, 10, 11, 12, 13, 14]. In parallel, we assess the policy requirements for incorporating community monitoring in MRV, and, where there are no absolute requirements, then the expectations of external agencies in terms of efficacy, economic efficiency and other expected benefits of community monitoring (e.g. [2, 3, 15].

The second methodology is an assessment of community responses in a pilot REDD+ programme in Mexico that has been implemented by CONAFOR (Comisión Nacional Forestal—National Forestry Commission) in four communities in western Jalisco state (for details See Sect. 6.2 below). We observed and investigated the communities’ initiatives and reactions to the REDD+ programme, and in particular their stated, observed and derived rationales for local monitoring and measuring, and sometimes also mapping (MMM). The methods employed were workshops, focus groups, community mapping activities, and formal community presentations. Additionally, we have included findings and observations from other fieldwork areas in Mexico about communities’ own interests in observing and monitoring their resources and territories.

Finally from a qualitative analysis of these grounded findings and consideration of the literature, we identify five challenges to reconciling communities’ desires for doing MMM, with REDD+ interests in MRV. We first examine the motives of external actors to support and encourage community monitoring for REDD+ MRV, before moving to an analysis of what communities themselves are seeking and employing in their community-based MMM.

## 3. External rationales for community participation in monitoring

Participation by its nature has to build up trust and confidence and familiarity, which is normally a slow incremental constructive process. Therefore it tends to slow down planning or management processes—monitoring or otherwise, and thus has costs. Moreover, participatory activities can frequently be confrontational and disturbing because their transparency often raises formerly hidden conflicts. Therefore, we need to consider the framing in which planners and decision-makers encourage local community actor participation in monitoring. The frames range from ‘participation’ being promoted by policy-makers and REDD+ specialists as a matter of principle because they believe a participatory effort will strengthen empowerment and devolved planning, to the other extreme that it is simply to ‘grease’ community acceptance and therefore the uptake of a REDD+ or other environmental management project.

Even where community monitoring is not essential for the national forest information system for REDD+ reporting (as we see below for Mexico), we can identify reasons why policy-makers choose to involve communities in forest surveys for REDD+. These reasons fall into two essential categories: (i) for improving the content and quality of the monitored information, and (ii), above and beyond that, for capacity-building towards community empowerment. Firstly we consider three aspects related to content and quality, and then two related to empowerment motivations.

### 3.1 Input to national databases

The value of community participation in monitoring for REDD+ in terms of boosting national data quality has been argued by e.g. [6, 16]. Data from community-based forest surveys have a more intensive collection scale. Detailed information on carbon stock changes at the community scale can densify and strengthen the national database and provide higher levels of credibility to data linked with remote sensing, since changes in biomass density cannot be reliably established without ground level measurements. Community monitoring can provide ground level data against which to calibrate remote sensing, and for identifying different forest types difficult to distinguish in satellite imagery.

### 3.2 Greater range and quality of indicators

Relative to external expert measures, community-acquired information can be quick and current (up-to-date), and therefore is likely to be more appropriate for early warning. There is local specific knowledge of species, land and forest qualities, ecosystems, indicators, threats, degradation, drivers, and more [7, 8, 10, 13, 17], and of process knowledge (forest management decision-making processes), especially in comparison with measurements and judgements from periodic visits by external experts [18]. In countries where local communities rely on third party experts to evaluate natural resources on communal land and develop management plans, as in Mexico, participation by local landowners in monitoring efforts can also serve as a quality control and assurance to data reported by outside entities. Errors in the collection or reporting of field data by these third parties can significantly affect a community’s ability to qualify for financial support from government programs, and locally-derived data can serve to clarify discrepancies.

In forest resource management, and notably in the use and management of NTFPs (non-timber forest products), it is essential to seek out and give value to gendered knowledge. In most forest-oriented communities, Mexico being no exception, certain tasks, activities, skills, and knowledge are primarily in the domain of women in the community. Therefore any community-based monitoring not only has to include women in the MMM activities, but even before that, it is essential to elicit and recognise this class of knowledge (e.g. in Mexico: [19, 20, 21, 22, 23]; and in some other countries: [24, 25, 26, 27, 28]).

Community monitoring is also able to supply valuable historical information on the drivers of deforestation and degradation and on the impacts of projects and programmes intended to mitigate these (e.g. [29, 30, 31]). For external funders such as voluntary markets, community-level information on performance and safeguards might be considered more credible and authentic than data based only on national level assessments. Besides, in some countries, there are also legal or policy requirements for promoting local participation.

### 3.3 Cost efficiency

It has been shown that community monitoring can reduce operational and transaction costs of setting up REDD+ projects [9, 32, 33], and there is also the positive outcome of local employment generation. Costs of community forest inventory have been estimated at between US$1 and US$4 per ha. p.a. [33], including day wages for the community members involved and intermediaries, and a factor for ‘rental’ of the equipment (PDA, GPS). Partly because standard forest mensuration procedures have been well developed for decades whereas community forest inventory is still an infant procedure, start-up costs of community monitoring are currently higher, due to the substantial inputs by intermediaries such as NGOs, in project development, training and establishing the sampling plots. Average costs are also much higher in the smaller, fragmented, mixed forests commonly held by communities.

### 3.4 Identification of local interests

Alert external agencies recognise that they do not really know what all the local priorities are. Stronger community participation in on-going monitoring would give local values more prominence in the design of projects, thus, it is assumed, making them more likely to succeed and be sustained. Engagement in monitoring strengthens communities´ forest management practices by providing feedback to themselves and the external agencies on the outcomes of the management decisions [5, 9, 11, 34].

### 3.5 Commitment and ownership

In terms of supporting empowerment, there is a belief among many development agencies that, when communities monitor, this encourages a more general participation in improved natural resource management. Community (or individual) involvement in a participatory process supposedly leads to more local acceptance, local understanding, and ‘ownership’ of an externally-driven activity such as a REDD+ or PES (payment for environmental services) project [12, 32, 34, 35]. Overall, there is improved governance, including more transparency in procedures. Empowerment develops social capital and local capacities and builds self-confidence within the community in specific areas like the handling of technologies, processes and procedures.

External agents, especially NGOs, may have strong ideological commitments towards participation due to their strongly-held values and beliefs. However there are also instances of deliberate deception by external actors, of the depth or continuity of local involvement for the political goal of keeping up a facade of participation.

In communities where youth often leave in search of opportunities in urban environments, monitoring efforts that are officially recognized and even monetarily supported can be an incentive for young people to stay and assume a variety of leadership roles. As computer savviness and technological know-how assume an even greater role in MMM activities, young adults form an important part of a community’s monitoring efforts due to their generational familiarity with mobile devices and the internet.

## 4. Communities’ rationales for monitoring

The significant question we address is how communities themselves are likely to benefit from such participation. We seek to identify the motivations behind members of local communities becoming engaged in externally-driven measurement and monitoring activities which can be relevant to national MRV (cf. [7, 8, 11] especially Part III, [13, 18, 36, 37]). The effectiveness, value added, and benefits to the community lie both in the specific products of the participatory activities, and in the processes of participation.

### 4.1 Territorial claims

Communities already monitor their territories, the resources within them, and changes in these. The significant driver behind much local monitoring of community territory and forest areas is people’s concerns with ownership and entitlement. It is therefore related to reinforcing claims for customary territorial rights and entitlements to the land as a resource which extends to making claims for lands lost or being invaded [38, 39, 40]. Holding land implies rights over a broad range of land-based forest products, not only the timber, hydrocarbons and other minerals and water resources, but also claims to wildlife and biodiversity, and to spatial services such as rights of way for roads or transmission lines.

### 4.2 Stresses and vulnerabilities

A rationale for community checking is to note the stresses of different kinds which are affecting traditional local forest management or NRM in general; for example, degradation locations and their causes, including livestock pressures, woodfuel extraction, deterioration of NTFP stocks, extraction of construction materials such as sand and gravel, and any land use change. The locations and impacts of natural hazards—notably forest fires, water pollution sources, forest pests and diseases, flooding, or landslips—are monitored; as are forest and vegetation management aimed at improving supply and quality of water. Expanding rapidly in Mexico and elsewhere are communities’ economic stakes in ecotourism, where they are seeking ways to monitor threats to the ecological status or the aesthetic quality of the landscape, as well as seeking new ecotourism opportunities (e.g. [41]).

### 4.3 Requirements of external environmental programmes

Many communities are already involved in formal natural resources management programmes such as PES for hydrological services, erosion control, biodiversity, endangered species, pollination, or landscape aesthetics. PES projects for environmental services, notably biodiversity services, require reliable, detailed measurements of environmental indicators at community level, and communities have been engaged by projects to gather data, usually on a paid basis or in return for services. Many communities have been engaged for some time with good effect in monitoring biodiversity (e.g. [7, 8, 9, 10, 11, 12, 13, 14]). There are however, significant social and behavioural differences between the specifics of monitoring biodiversity, and monitoring carbon stock changes. Monitoring biomass carbon stock changes implies dealing with an ultimately invisible product not directly linked to traditional culture or local indigenous knowledge, it is tough work in the field, a very high level of precision is demanded, there is a time delay of several years before seeing useful results in the REDD+ framework, and essentially, it provides scant incentives and less fun for the keen youth who energetically monitor the presence of jaguars or rare plants.

If the community already has forest lands which are under certification schemes for timber, or forest products and forest quality, they are usually required to carry out intensive monitoring and verification (e.g. Forest Stewardship Council, Global Canopy Partnership). The motivation here is the increased value of the products in national or international markets. There are also non-timber products which have both livelihood and commercial value to the community, e.g. bamboo, honey, medicinal or house plants, which can require monitoring and verification.

### 4.4 Staking claims for political recognition

Communities increasingly are recognising that the ownership of the information on carbon stocks is crucial to establish their rights over carbon and their access to REDD+ rewards. Beyond this, there are political-institutional reasons for involvement, for example a need felt by the community to be ‘on the stage where things are happening’, in order to build a position for negotiation and benefit-sharing, or to spot opportunities in public programmes [42]. Likewise, community monitoring can be held up as an element of the community’s responsible management of forest territories, thus helping to justify their tenure claims.

## 5. Community monitoring data tasks for REDD+: MRV vis-à-vis MMM

In the literature, the focus on community monitoring for REDD+ tends to be on the immediate forest inventory tasks of measuring variables such as DBH (diameter at breast height) and identifying species, etc., but in fact, monitoring requires much more than this. Prior to making tree measurements, there is a need to map and classify types of forest and other woody vegetation to be included under REDD+, and to lay out a sampling frame to ensure the data gathered are unbiased statistically and sufficient to reach levels of certainty. Many of these tasks are generally considered technical and difficult for local people to carry out themselves, and are commonly done by external agencies. Furthermore, depending on the nature of the national REDD+ programme or project procedures, there are requirements to gather data on socio-economic variables, including on the achievement of safeguards ([5, 15, 43]; see Table 1). For consistency across a whole country, and if data are to be entered into a national database, MRV requires pre-prepared protocols which define to a high level of detail what data are to be gathered and how.

**Table 1:** Information for Community Forest Management and Carbon Sequestration.

**A. Spatial information for establishing the initial management scenario (project year 0)**	**Key characteristics:** Reliability of Source / Scale and Extent / Precision / Timeliness and Frequency / Replicability
Boundaries of the community and its forest areas intended for carbon payments project	High Precision
Community’s land claims	Essential local spatial knowledge, and of neighbours; Sensitivity
Community forestry management systems and approaches, land-use plans	Essential local spatial knowledge
Location and sources of forest degradation -: (illegal) logging, grazing, marginal agriculture, (illegal) settlements, hydrological adjustments	Essential local spatial knowledge
Locations potentially affected by hazards (e.g. fires, erosion, ecosystem damage, flood, storm)	Timeliness
Conflict areas	Essential local spatial knowledge; Sensitivity
**B. Information for forest biomass inventories (project year 0 and later)**	**Key characteristics**
Delimitation of forest ecotype strata (zones)	High precision
Location and geo-referencing of sampling plots	Very high precision; Replicability
Geo-referencing trees and features for future locating of sample plots	Ditto
Field measurement and storage of tree data: DBH, tree heights, species, status, etc. in databases	Ditto
Assessing leakage	Sensitive; Leakage extends outside the community, monitored at higher spatial scale but using local data
**C. Monitoring of Safeguards, and monitoring of social and environmental variables**	**Key characteristics–similar for all.** *Some do not have spatial indicators*
Conservation of natural forests and biological diversity	Essential local spatial knowledge; Reliability of sources; Sensitivity; Spatial precision and timeliness are not high priority
Human rights—especially indigenous and forest communities	Ditto
Transparency and effectiveness of national forest governance structures	Ditto
Respect for knowledge and rights of indigenous peoples and forest communities	Ditto
Full and effective participation of actors	Ditto
Equitable internal distribution of benefits	Ditto

The question is whether communities are interested in producing such standardised data for REDD+, and under what conditions. We propose, as a general principle, that the concept of community-based MMM (measuring, mapping and monitoring) is more apposite than MRV. Under MMM, measuring, mapping and monitoring are primarily for local purposes and for local users, and activities are essentially designed by communities themselves to meet local requirements, interests and priorities. Measuring, mapping, and monitoring are interrelated components of spatial information acquisition, three dimensions of information relating to an object of interest. *Measuring* is the dimensional component, the description of the item itself. *Mapping* refers to the spatial dimension, knowing where the object is in space and its spatial relations with other objects. *Monitoring* is the temporal dimension of the object over time, i.e. changes in the measurement of the object over time. The three components together add up to the full descriptors of the biomass and carbon dimensions: tree species, indicators of causes of degradation and deforestation, watershed management indictors, forest management practices, forest tenure, measures of social welfare and equity, and so on.

## 6. MRV and MMM: cases in Mexico

### 6.1 Community Territories, Forests and Carbon in Mexico

In Mexico, 55–59% of all forests fall within the territories of autonomous agrarian communities [44, 45]; these form the basic rural landholding units of the country, together with private properties, which account for at least 40% of the forests. Mexico´s REDD+ strategy involves a broad approach to sustainable rural development, in which communities and private property owners are heavily involved.

In terms of MRV, a national reference emissions level has already been proposed by CONAFOR [46], against which Mexico´s REDD+ achievements as a whole in reducing deforestation will be assessed, and compensated—in the immediate future, through the World Bank Carbon Fund. Each state will develop its own baseline. These do not include degradation or forest enhancement, although the carbon saved is considered to be property of the nation, and not of the states nor individual communities [47]. It is envisioned that the funds will be shared within the country on the basis of REDD+ relevant investments required, rather than on the basis of carbon performance. Importantly, this implies that there is no immediate need for baselines or for monitoring sound forest management at the community level, nor for leakage assessment as this would be tracked at the state or national level.

As currently conceived (mid-2015), any increases in sequestration (forest enhancement, growth in forest stocks) that communities achieve are intrinsically the property of the community, as distinct from carbon savings from reduced deforestation and degradation which accrue to the nation (see above). This means that in principle communities would be allowed to sell credits for any such carbon on any voluntary carbon market, and for this, both a local baseline and local monitoring would almost certainly be required. There is ample room for many communities to ´grow more carbon´ and this strategy is both sensible and convenient since, (1) it is much less likely to result in leakage than a strategy focussing on reductions in deforestation and degradation, and (2) communities cannot measure changes in deforestation and degradation in any case, because they do not have stock assessments for previous periods. What they can do is measure stocks today and in subsequent years to estimate the increases achieved.

Community monitoring is not currently used for the national forest information system supporting REDD+ in Mexico, nor is it used as the basis for monitoring the distribution of benefits within the national REDD+ programme. In this paper, we do not enter into the multiple social-political issues involved in ´fairly´ distributing benefits from carbon projects (see e.g. the case of benefit monitoring in indigenous community forest projects in Bolivia in [36]); rather we focus on the already complex-enough issues in community monitoring of the forest resources and carbon. Nevertheless, CONAFOR, as the forest agency responsible for REDD+, is developing community monitoring protocols, not only for carbon, but a variety of indicators. The objective is to develop a standard framework broad enough to cover communities´ own interests and to generate information that is required for participation in government programs aimed at natural resource management and sustainable rural development. A possible use in the long run for these data is to strengthen the national database and national carbon estimates for REDD+.

### 6.2 The LAIF Project

CONAFOR partnered with the Latin American Investment Facility (LAIF) http://www.conafor.gob.mx/web/temas-forestales/bycc/acciones-de-preparacion-para-redd/gobernanza-local-para-implementacion-de-atredd-laif/ to channel funds to develop land management plans for local watersheds in priority forest areas in Mexico and act as the principal agent for the implementation of REDD+ pilot programmes in *ejido* communities. For a century since the land redistribution of the Mexican Revolution, a central feature of the legal structure of land tenure has been community governance structures (*ejidos*) built around shared land and democratic decision-making processes. *Ejidatarios* (*ejido* members) are legal landowners and all the decisions made regarding land use and development take place within the *ejido* assemblies. This creates, in theory, a transparent political system open to all *ejido* community members, which feature is fundamental to the community MRV pilot project spearheaded by LAIF. It is from within these local decision-making structures that we can identify what communities prioritise to be their locally-specific benefits of monitoring. Every *ejido* operates according to a locally-specific set of livelihood and cultural practices, and each uses its forest resources for specific purposes and approaches them with different skills and knowledge bases.

CONAFOR-LAIF approached four *ejidos* to participate in the development of MRV-focused community monitoring pilot programmes in the state of Jalisco, and later, one in Quintana Roo. A prerequisite for involvement was that the communities fell within the jurisdiction of a *Junta Intermunicipal* (Inter-municipal Board), a multi-party, regional decision-making body formed under the LAIF project in order to decentralize environmental governance and empower policy-makers at the watershed level (S1 Appendix). Since then, this REDD+ pilot concept has been replicated by Alianza MREDD in the states of Oaxaca, Chihuahua, Yucatan and Campeche [48] and these pilots have provided further relevant experiences of how communities participate in monitoring. The piloted framework included the endogenous identification of key resources for monitoring that could then increase the local capacity for effective decision-making and sound land and forest management. Internally, the *ejido* decides which resources in their territory are of most importance to them and what tangible benefits are to be gained by collectively monitoring these. This process re-emphasizes the tailoring of the framework to the specific community contexts. Carbon was never explicitly mentioned, but the resources chosen by the community groups are all related to reducing forest degradation and improving forest health, which is the fundamental tenet of REDD+.

Any community member officially recognized as an *ejidatario* by the local *Asamblea* (Assembly) could volunteer to participate in the creation of a natural resource monitoring committee which the *ejido* leadership would then officially recognize and sanction. Thirty community members joined the first four monitoring committees, with the actual selection criteria being the responsibility of each specific community. There was thus an average of over seven self-selected, but community-approved, experienced people on each committee, in *ejidos* whose populations ranged from 50 to 100 families. Part of the requirements for the pilot LAIF programs was the internal identification of the members of the monitoring committees, the consistent interaction of these committees with the *Asamblea*, and the inclusion of forest resource monitoring into the legal architecture of the community. The LAIF team, while striving to respect traditional roles and leadership in the community, urged each participating *ejido* to recognize the contributions made by non-traditional actors in land management activities, such as women and young adults when selecting the committee members. Each *ejido* responded in various ways, with some recruiting men who were already members of other committees and others having both women and young men volunteer to take on specific roles (S1 Appendix; S2 Appendix). There is much unfilled potential for more equitable representation in MMM activities in terms of gender and age (cf. [25, 26]), as communities are provided with the tools to identify and monitor locally-relevant environmental, social and economic indicators.

### 6.3 Community motivations for MMM

Group brainstorming activities, key informant in-depth discussions and field visit observations in the *ejidos* of the LAIF project revealed the following priorities for forest monitoring (S2 Appendix; S3 Appendix). We also take note of fieldwork findings in other *ejidos* in Jalisco and Michoacán states, and from external literature. The names of the non-LAIF *ejidos* are kept confidential.

Before examining the positive motivations, it is necessary to note that, apart from the cost and time involved, there are other sound reasons why a community may choose not to monitor, at least not to share its information with the outside world. The protection and conservation of valuable and sacred places and artefacts can be a concern, with a fear that monitored data will be appropriated and used for the benefit of outsiders, such as the community being robbed of resources or control over them, a process popularly known as eco-piracy ([28]; see Sect. 8.2). Sometimes there are deliberate attempts to hide information, for example the location of sacred places or of rare plants and of minerals.

#### 6.3.1 Requirements for external certification

Rainforest Alliance and FSC certification for sustainable harvest, for example, stipulate that a monitoring program must be in place and actively contributing to timber management plans. In most such cases, *ejidos* pay a third-party consulting company to develop a management plan, execute its implementation and generate reports. However in the pilot programmes, *ejidatarios* stated that developing the capacities to carry out such monitoring plans internally provides new skills for more community members to participate, it increases land-user familiarity with forest management techniques, it adds a second layer of verification to the information generated by an outside consultant, it saves money, and it places more authority in the hands of, at least the *ejido* leadership, and maybe, the community at large.

#### 6.3.2 Forest health and ecosystem benefits

Two of the participating LAIF *ejidos* identified forest pathogens as the main threat to their communal lands, specifically the rapid and uncontrolled spread of dwarf mistletoe (*Arceuthobium spp*.). Both stated that their local timber-based economies were threatened without a comprehensive plan to monitor the spread of the pathogen and the outcomes of interventions. Other ejidos were concerned to monitor the health of their pine forests for pests, diseased trees, or *arboles bifurcados* (forked, twisted, or excessively branched trees), and in other cases, monitoring afforestation efforts with *ocote* (Chiapas pine). In another timber-intensive *ejido* in Chihuahua, very large in area but with a small resident population, wildfire monitoring was the main motivation [48].

#### 6.3.3 Wildlife habitat and forest aesthetics for conservation, cultural heritage and ecotourism

Table 2 lists a number of animal and insect species that community leaders or monitoring teams identified as being of special interest to monitor, regarding their changes in population, location, habits, and threats. In many cases, perceived opportunities for ecotourism were identified as a direct reason for establishing a local monitoring program; and/or other communities were proposing to develop UMAs (*Unidades para la Conservación*, *Manejo y Aprovechamiento Sustentable de la Vida Silvestre*—Management Units for the Conservation of Wildlife) based on the conservation of specific wildlife. One *ejido* is intending to monitor and track damage from off-road motor biking and quadbikes, in part because of its impact on ecotourism income.

**Table 2:** Wildlife Monitoring Interests, in LAIF and other Communities.

Animals, Insects	Monitoring the distribution, changes in population, location, habits, and threats
Large felines–especially jaguar, puma	Cultural heritage, potential ecotourism
*Venado cola blanca* (Whitetail deer)	Hunting for food or sport, population management & conservation, potential UMAs
*Jabalí* (peccary, wild pig)	Hunting for food or sport, population management & conservation, potential UMAs
Beaver, squirrel	Pests, hunting
Snakes, e.g. rattlesnake	Potential for herpetarium, medical use–antidotes to poison, cultural heritage
*Lagartos Ponzoñosos* (Gila lizard)	In danger of extinction, cultural heritage
Cicadas	Conservation for ‘beauty’, propagation, potential for ecotourism and UMAs
*Lombrices* (earthworms)	Important for soil quality and plant growth
Enos butterfly	In danger of extinction, cultural heritage, potential ecotourism
Amphibians, e.g. endemic salamander *Ambystoma*	In danger of extinction, potential ecotourism

Sources: Fieldwork with LAIF and other communities.

#### 6.3.4 Water supply and quality

Many LAIF *ejidos* selected water as the main monitoring priority and identified ways in which water supply and quality are related to forest health. One coastal *ejido* unanimously voted water as the most critical resource to monitor, because of its diminished supply due to cattle grazing. This monitoring committee was interested both in collecting information on current water supply and monitoring the effects of reforestation projects on water infiltration and retention in their cloud forests. They specifically wanted to ensure that their communal funds were being invested in successful replanting projects, and saw monitoring as the way to observe changes in land cover, soil erosion and water resources so as to inform community spending. Another *ejido* chose to monitor water quality in areas with ecotourism opportunities. Committee members stated the importance of the knowledge and tools to keep track of water quality to guarantee eco-tourist visits. Information generated from water quality monitoring informs discussions and local decision-making at the *Asamblea*.

#### 6.3.5 Monitoring forest resources for livelihood materials

There are a broad range of products from the forest–plants, foods, NTFPs, building materials and so on–that communities already keep a regular and deliberate eye on (even if not in a formal format) or the monitoring committees in LAIF expressed interest in systematising. Lopez et al. [21] and Toledo and colleagues [49] have enumerated more than a thousand (1,052 in primary and secondary forests) of these. These include mushrooms and other forest foods for local consumption and market—note that these types of products are usually in the domain of specifically women’s knowledge. Other cases include communities wanting to check the rarity and risk status of orchids whose market and ecotourism values are well-recognised, in addition to their intrinsic cultural heritage; potentially their sites could be set into an UMA.

In Chihuahua state, LaRochelle and Berkes [20] investigated an indigenous Sierra Tarahumara community monitoring edible, medicinal and other usable wild plants in their landscape as part of traditional ecological management. Klooster [30] studied a Purhepecha community in Michoacán monitoring the removal (including illegal entry by neighbouring mestizo communities) of woodfuel supplies for pottery kilns, brick-making and charcoal.

#### 6.3.6 Monitoring land invasions and threats

This monitoring mainly involves actual and perceived threats to the territorial integrity of *ejidos* by neighbours–whether those are other *ejidos* or rural communities or private land owners—who are directly, or potentially might be, invading and utilising the land for grazing, cropping, illegal logging, artisanal mining, extracting woodfuels or a host of other reasons (c.f. [30]). Other cases relate to land grabs by external powers such as mining companies, but usually these are too big a scale to be the concern only of the local community. In some cases, the threat is internal, i.e. some community members may be appropriating for themselves what are supposed to be communal land resources, such as converting (formally) communal forest lands to pasture or to tree crops (e.g. [39, 41]).

### 6.4 Tools of the trade: training communities in MMM in Mexico

The CONAFOR-LAIF project team worked with professionals experienced in forest mensuration and resource management to develop hands-on field trainings specifically designed for rural property holders. Team members designed a protocol that could accomplish three main goals: 1) sufficient standardization of steps and phases so that *ejidos* could progress in the same timeframe, 2) the protocol could be replicated in other *ejidos* in the future, and 3) each *ejido* could tailor it according to their locally-defined needs, strengths and cultural norms (S1 Appendix; S3 Appendix).

The protocol set up nine specific activities for each *ejido* to undertake as part of the pilot programme. Key activities were: a community-based diagnostic of local natural and cultural resources, identification of local monitoring priorities, community-led resource mapping exercises and a field tour of potential monitoring locations; these were followed by field training, setting a community monitoring work plan, then field data collection, and finally, data interpretation (S3 Appendix). All of these were facilitated by the CONAFOR-LAIF team together with the monitoring committees in each *ejido*, and were regularly subject to approval in the *Asambleas*. Mapping techniques played a critical role in all training activities. Monitoring mainly consisted of direct observation (ground truthing) and photo documentation, but the communities wanted also to use more technical tools including GPS, video, tracking apps such as CyberTracker, GIS, and reporting apps with text messages or web platforms.

The majority of the activities were completed within one day. The exception was the field training which was conducted over two days in each *ejido*, in order to adequately cover the range of field topics and give monitoring committee members sufficient time to become proficient in using field equipment. Participation varied according to each community and depended on the number of people who volunteered to join. The smallest committee had seven consistent members, whereas the largest had upwards of fifteen. Participation also depended on the specific activity. For example, the field tour in each *ejido* involved the largest groups, allowing for broader and deeper inputs regarding potential monitoring areas. The great majority of monitoring committee members were men, who have traditionally assumed roles in community natural resource decision-making. Only one *ejido* had a woman who volunteered and she was later officially recognized as the project leader. However, all the *Asambleas*, to which the monitoring committees report their work and results, were of mixed age and gender. The ages of participants varied between *ejidos*, largely dependent on who was actively engaged in resource management. In the smallest *ejido*, members were generally in their 50s and older, whereas the larger *ejidos* had more participation by landowners between 30 and 50. This could be a function of population size, because smaller communities have fewer potentially interested parties (S2 Appendix).

In addition to resource mapping and locating priority sites, participants gained exposure to natural resource monitoring with field measurements for specific resources, i.e. estimating timber stocks and growth, area infected by mistletoe, water flow rates, water chemistry and contaminant loads; and also sample design, data recording; data sheet creation for monitoring, and basic data analysis and techniques for making presentations to the *Asamblea*.

Young adults are more likely to participate in tech-based monitoring, owing to their generational familiarity with technology. The potential for this kind of exercise lies in the ubiquity of mobile IT devices and apps which have rapidly increased functionalities, at lower cost, and are becoming easier to handle. Hardware such as rugged Tablets and Smartphones with large memory for imagery or maps, with GPS capability, camera, video, and internet connectivity are replacing the PDA set-ups used in the first trials for carbon monitoring [50]. Geo-referenced images as bases for mapping forest are easily available at very low cost or free, from Google Earth, Virtual Earth or other ‘virtual globes’. The cost of LIDAR which provides very high precision imagery is dropping. There is big potential in UAVs / drones for communities to acquire their own dedicated imagery from air-borne sensors and their own capacity for real-time monitoring of forest threats—fires, pests, or invasions [51]. Apps with user-friendly interfaces are being adapted for forest and tree measurement with simplified data recording and interfaces in Mexico, in particular, CyberTracker, Plataforma eREDD, and Google’s ODK (Open Data Kit) and GeoODK [15, 42, 48, 52].

## 7. Five challenges to reconciling community MMM and MRV needs in REDD+

The five issues discussed below are in increasing order of complexity in terms of socio-cultural and political situations in communities, and the relations between communities and REDD+ demands, and are therefore also increasingly complex in terms of seeking solutions or amelioration.

### 7.1 Quality control and timely supply of data in measuring carbon stocks

Quality of carbon data is essential from a REDD+ MRV perspective but much less so from the perspective of communities themselves. It is clear that if data are to be used in external systems–a national database, or to satisfy conditions of particular donors or carbon purchase systems–communities will have to accept standardised protocols of one sort or another. Moreover, punctual reporting of outputs of community monitoring MMM will be demanded by whoever is acting for the REDD+ agencies, and sufficient detail and precision will be required. Because the data are needed at regular but infrequent intervals, training exercises and processes will have to be set up and repeated over time.

The requirement for frequency and regularity of data supply are more likely to cause friction between external agencies and communities than the quality of the data itself. In the few studies specifically examining the performance of local measurers following pre-determined protocols, the results are generally positive [9, 12, 33]. Although some have expressed doubts whether communities will be able to provide reliable, unbiased, good quality data [37], the evidence is that they can. In the K:TGAL project, independent professional forest companies carried out surveys in order to test the reliability of the communities’ estimates of carbon stock [33]. In every case, there was no more than 5% difference in the estimate of mean carbon stocks between the professionals and the community [15]. That field measurements are made equally well by community teams as by professional surveyors, does not necessarily mean that the accuracy is high. Measurements are often made rather rapidly, by both groups, with a variety of errors entering the process. The main challenge is the precision of DBH measurements which can be compromised by measuring DBH at the incorrect height, using the tape too slackly, or missing some trees [53]. These matter less for an initial survey, but more if the same trees are re-measured in permanent plots to estimate very small growth parameters.

Using field data recorders and apps to record and store the data probably reduces errors—the data are recorded only once, meaning only one opportunity for error in transmission, unlike in recording on paper in the field. It is possible to introduce filters into the software, such that if an unlikely figure is entered e.g. for a DBH of a particular tree, the computer prompts a query and the error is correctable at source. But it is always recommended to keep a hard copy of the data in the field as well, and accuracy of the data and their analysis does improve with repetition and training [42]. If permanent plots are set up by the community for their monitoring exercises, there may be a tendency for additional exceptional protection of these, such that they are no longer typical of the forests in that area; for example, being protected from cattle grazing, or from NTFP and timber collection. On other hand, the measurement process itself (DBH, height estimates, understory biomass measurement, soil carbon) creates damage through trampling, disturbance, paths, and therefore measurably reduces biomass and carbon in the target area. Training sufficient trainers for carbon stock measurement could also be a major problem.

### 7.2 When conflict avoidance hinders monitoring–leakage and degradation

Measuring leakage (which would occur in neighbouring communities or elsewhere in the region) is an issue that has not been carefully thought through. Leakage is like a waterbed, push down on practices which cause deforestation and degradation in some place, and inevitably they pop up somewhere else. The degradation practices are often in grey areas between external (official) legality and customary practice. They are very likely to be bound up in customary rights, entitlements, and local activities. Monitoring and reporting of leakage can exacerbate or create discrepancies, contestations and outright conflicts within and between communities. Therefore it is not easy to integrate leakage information into community-based MMM. Communities may willingly report leakages from other communities which negatively affect themselves, but they are less happy to report their own leakage into other areas.

### 7.3 Selection of participants and sustainability

The question of who carries out the monitoring is important. Are participating community members self-selected, or are they chosen by external experts? Do they originate only from involved NGOs? Is it an obligation, or can anyone choose to join in? The idea that community monitoring is advantageous because there is an unlimited labour force pool is questionable. In the pilot projects in Mexico there were plenty of young people (male and female) available and interested in getting out in the field and mapping/measuring the biomass when the team from outside arrived. They were relatively under-employed and willing to learn. But this approach is not necessarily sustainable—these people may not be there on the next monitoring date, and it is highly unlikely they form a permanent cadre of monitors in the village. The ´best´ young people tend to leave - ´best’ in the sense of having the technical skills, interest and energy. New youth have to be trained, which implies continuing overhead costs, and there is no build-up of a reservoir of accumulated skills in measurement. Garcia and Lescuyer [54] considered this as one of the reasons behind the collapse of so many community monitoring systems. In Cambodia, the IGES CCA Project claims that community teams retain their learnt skills—in 2012 they observed a community monitoring team which had received training a year earlier who demonstrated they had retained their knowledge and skills—“Local people who participate in a well-designed training programme can be relied upon for future forest assessments” [55]. We agree, but the determining factor is: only if those trained people are still there.

One question raised by the LAIF project is how will the ability of the community itself to finance monitoring efforts incentivize young adults to advocate for and commit to this type of employment? The young people who participated in the pilot programs were duly acknowledged in the *asambleas* for their contribution to monitoring efforts, yet long-term commitments to finance their positions, either by themselves or by the *ejido*, were not investigated. Although working with older community members is more stable, the drawbacks are a slower learning curve and less energy—it is hard work taking biomass measurements out in the forest. Involving women in monitoring efforts touches on complex socio-cultural dimensions that vary from one community to another. Mandatory inclusion can upset local norms and cause conflict, taking time away from other responsibilities and creating stress in the community. The LAIF project indicates that communities which are empowered to self-organize and identify priorities for MMM activities select those they consider to be appropriate participants to guide these efforts. Further investigation is needed in applying this model to identify which priorities women, youth, the landless, and other groups not traditionally involved in land management decisions identify as important to their community’s health and wellbeing and who would subsequently participate as part of the monitoring team.

### 7.4 Incentives and cultural frames

Concerns arise as to whether the monitoring team, if say it is selected by external agents, becomes an elite group which can capture benefits not available to the rest of the community, and whether appointment to such a monitoring group implies favouritism within the village community. If monitoring is a paid activity with monitors receiving a daily wage, then there is a risk. Therefore a social alternative is payments into a community fund instead. In moving towards MMM, the community should itself select the ´best´ persons (i.e. those with the most appropriate skills and attitudes), and create a distributive system for monitoring, such as rotating duties. The merging of MRV with MMM, with their differing requirements, is problematic in this.

Engaging communities (and individual actors) in monitoring requires addressing the issue of participation “in breadth” vs. “in depth”. There are plenty of downside difficulties for local community actors who want to enter into MMM activities—involvement in MMM is not easy and people do not choose to do so lightly. There is a limited number of actors who for personal belief reasons engage “deeply”, that is, commit to and meet the challenges of intensive, time-consuming participation, perhaps across many stages of an MMM process. But are these ‘volunteers’ a representative constituency of the relevant community? Alternatively, will an MMM process that involves a larger number of participating actors be sufficiently meaningful in the depth and usefulness of their engagement? Big issues of compensation arise here, with many externally-promoted projects expecting that participants in monitoring will be donating their time and effort as well as their knowledge, without direct financial compensation, because ´it is in the long term interests of their community´. Whether people are willing to do this will be much determined by who decides on the types of data to be gathered and where they go—gathering carbon data to feed a national database with no direct return to the community, or gathering carbon data for some community purpose.

In REDD+ discourse there are proposals for financial payments to be specifically for monitoring for MRV, and not for the carbon enhancement and credits per se. This would be a paid employment, structured by skills training, registration, and independent (re-)testing. The payments could be to the community members doing the work, in fair compensation for labour time and any disruption to other tasks (consider, peak labour periods), and for risks. Direct payments for work accomplished are seen as a distinct positive for the community. The intended advantage of such a protocol in terms of data quality and security is that there would be less incentive to tweak the results and exaggerate carbon gains/understate carbon losses. But in reality the local community surveyors would be well aware that their measurements would have significance for the continuation of payments to any REDD+ project. Therefore the key is that the local surveyors would need to be convinced that it is the *regularity* and *consistency* of their measurements which have significance for the continuation of payments.

Garcia and Lescuyer [54] concluded from their review of 11 cases of community monitoring of natural resource management, that any ‘community income resulting from better monitoring‘ is likely to be lower than the total costs of participatory monitoring, and this is what brings about much of the abandonment that they observed. But the prospect of paying communities for monitoring raises another contrasting critique within some communities, who ask: ‘why be paid for activities which communities are doing anyway?’ Some communities even feel that it is a devaluation of their efforts and denigrating. In Mexico, these critical views concerning payments for community-based MMM or indeed for community-based forest management activities in general are particularly heard from indigenous communities, rather than in the *ejidos* where many such changes have already happened. In this vision, financial incentives are seen as driving a monetised attitude towards the environment, and as exacerbating a loss of youth interest in the traditional customary management of forest lands. Elders fear that young people will come to expect direct financial benefit from what was formerly collective and voluntary labour.

### 7.5 Conflict of purpose–mapping land

Communities monitor their territory and forest areas in the context of claims for customary territorial rights and entitlement to land resource, and for making claims for lands lost or invaded by other people. In REDD+ MRV, there is an underlying set of items to be mapped [15, 43], but the bottom line is that the lands need to be defined, identified, classified, measured and mapped—and here the trouble begins. Among many local and especially indigenous communities there is the concern that external global drivers behind such mapping exercises go beyond the practical immediate needs of REDD+ MRV, and towards deeper political-economic intentions. The stated purposes behind the mapping needs of REDD+ are found in the recommended good practices and guidelines ([5, 15]; see Table 1). We can summarise them as: (a) ‘*resource mapping’* to simplify, classify, and spatially zone the forest resources and uses of the forest; and (b) ‘*behaviour mapping’* in order to assess different types of management of forest / carbon landscapes, and some safeguards, and to understand the interrelationships between people and their forests. Both are necessary for planning and management and for allocation of payments. But the concern and the risk for affected local and indigenous populations—to whatever degree that is well-founded—is that there is a hidden third driver in REDD+, that is, (c) ‘*appropriation mapping’*, as an intentional, but non-transparent step towards the appropriation of local/indigenous territory. This concern can be found in the stances on REDD+ taken by many indigenous groups (e.g. [56, 57, 58, 59]). As people’s perceptions of the intentions of REDD+ mapping processes move from (a) along to (c), the conflicts sharpen, between what are REDD’s drivers for mapping changes in carbon stocks, and the people’s own interests in mapping their forest land resources.

## 8. Key messages and directions

### 8.1 Trust and confidence–credibility and acceptability

Encouraging and facilitating participation depend on confidence-building and trust, especially between the ‘professional REDDers’ and the local community actors. A critical problem in all participatory methodologies is the need to convince higher policy-making levels (i.e. higher levels than the local carbon survey team) of the validity, credibility and scientific ‘soundness’ of the inputs and products of local ‘non-professional’ surveyors. This issue of acceptability appears not only within the MMM exercises per se, but ultimately when their results are being assessed and implemented by the epistemic community of REDD+ scientists and national decision-makers, and also the general public.

### 8.2 Sensitive knowledge

MRV carbon surveys for various applications in developing national databases want to collect a large amount of detailed and spatially-specific information, not just on biomass growth rates, but on many topics which are sensitive for legal, social, economic, cultural or even spiritual reasons. Surveys can reveal confidential, sensitive information to outsiders, and can easily raise or exacerbate conflicts with the neighbours, especially stirring up the sleeping dogs of boundary disputes. There may be reluctance to report negative impacts or activities within the community which are, from the official point of view, illegal, and from the local point of view, sensitive. Besides leakage issues, these could include illegal uses of forest land, invasions, drug production, etc. Many more activities are semi-illegal but customary, long established activities such as collecting NTFPs, cattle grazing, hunting, etc. Moreover, in many forest-linked communities, especially indigenous communities, there are places and activities which are considered internal secrets, such as sacred sites or the location of rare plants with medicinal and financial value ([20, 60]; cf. [39, 49]).

Whether these are officially legal or illegal, people will be reluctant or absolutely unwilling to divulge them; and this is especially complicated when the local knowledge is gender-specific or otherwise highly significant to a particular sub-group. A simplistic approach to ‘community self-monitoring’ will not resolve this issue. There are incentives for community surveyors to hide or disperse such information (for the ‘communal good’ of the community, or for their own safety); alternatively, they are liable to accusations of being a spying unit. A solution to this could be that the local data transferred to the national REDD+ authorities, should not be geo-tagged to link them to the specific community. Of course they are geo-referenced, otherwise there could be no time series surveys of growth rates, etc. But the data could be treated in an analogous way to population census data, that is, the figures would be used anonymously to estimate sequestration and emission rates for particular forest types and regions (and cross-checked by satellite data at a coarser scale). By not routing the specific data measurements back to the specific communities, two challenges are reduced–the incentive for field data figures to be adjusted (so as to present the local situation in a more positive light), and the reasonable fear of communities that they will be held accountable not only for ‘negative’ changes to carbon, but also for the identification of ‘undesirable’ activities in their neighbourhood.

### 8.3 The power of land

Community-based MMM for carbon, biodiversity or other environmental services has potential significance for communities who are trying to consolidate their claims to places and land [61, 62]. Therefore, connecting monitoring to formalising and enforcing local land titling, and making it a condition for project entry, are a powerful incentive in many countries—although it is not such an issue in Mexico where communities already have full legal rights over their lands. Therefore, the key to encouraging communities to engage in MMM activities which are compatible with REDD+ monitoring, would be to collaborate with their claims against the loss of territorial rights and of their entitlements to land resources. This would strengthen their defences against illegal invasions, or the legal expropriations of traditional lands.

More existentially, for many forest communities or their representatives, the implementation of REDD+ and even the discourse itself, are felt to be part of a process of switching away from acknowledging the material land resources of a community as locally-claimed and owned territory. An accepted recognition that the land belongs to the local (frequently indigenous) forest community is perceived–by these communities—to be being replaced by a global discourse which could be interpreted as ‘carbon in trees’ is a common property with global environmental value, and thus a global patrimony. The implication of this discourse would be that these forest lands would be taken out of, and beyond, the specific responsibility of the people who live there. Underlying deep concerns about such a disastrous outcome for forest peoples are a continuing political and social barrier to communities’ involvement in REDD+ and its monitoring activities (see e.g.: [56, 57, 59, 63, 64]).

### 8.4 The future–MMM in place of MRV

There are plenty of reasons why local community actors may not want to get immersed in MMM activities. Participation is always slow by procedural design. It can be very time-consuming, maybe clashing with other activities in people’s livelihoods, and may not reach conclusions which can be used by the community itself. On the other hand there are many specific reasons why communities are motivated and are already involved in mapping and monitoring their own local environmental conditions and changes, or have a serious interest in doing so. We need to be clear that carbon is not usually the priority, and to ask who the information is for and why it would be useful for the community. “Communities are not interested in biodiversity and safeguards, but about species they eat, pollinators, pest controllers, and other species that have sacred value. It is exactly the same when we ask them to collect information about carbon.” ([52], p.6).

Community-based long-term (carbon) monitoring is more appropriate where local people have other active significant interests in knowing the status (stocks, changes, threats, potentials) of natural resources, environmental services, or other indicators of territorial well-being. Most communities have informal systems of checking or monitoring; they notice changes in forest condition and climatic parameters, they can tell if things have changed over a number of years, and they discuss the reasons for this in their assemblies. However the information is rarely formally recorded, quantified or systematised—which are the essence of a multi-level (national) monitoring system [11, 12, 42, 65].

If the monitoring activities are not for the community’s own interests as above, then a system based on local people carrying out designated tasks for a higher-level REDD+ authority will only be sustainable when the data and any benefits of the monitoring are perceived and experienced locally. In the case of REDD+, there must be a clear link between the monitoring efforts and visible benefits to the community, whether in the form of carbon credits, or social infrastructure services, or recognition of land rights, or direct cash payments for labouring in the monitoring. In the LAIF case, *ejido* committee members acknowledged the empowerment experienced when they were able to generate information that is seen as useful and valuable by their community. The combination of hands-on technical training and full, legal backing by the collective *ejido* is fundamental to sustaining interest within communities in investing time, resources and people in an exercise that does not generate a direct income to its participants.

All, or some parts, of the fine-detailed external MRV data requirements could be incorporated within the less formal but rich observations and checking that are part of community-based MMM. Since community members are unlikely to volunteer to meet all the detailed MRV conditions and procedures, probably some amount of external payment would still be needed, but less than if the community were doing only MRV. Therefore it is pertinent to look for funding MMM in the context of other programmes; for example in Mexico, in the *Ordenamientos Territoriales Comunitarios* (community land use planning) which are being promoted towards more sustainable development in rural areas.

The local specificity of community monitoring is the key positive factor that makes community-based MMM attractive for local people, who make use of it to raise awareness and deal with problems relating to their own resources, threats and potentials. The MMM of resources, threats, potentials, and problems is precisely what the community is looking for—they are interested in local MMM of local issues, whereas in C-MRV, localness is a negative. National policy therefore needs to recognise the distinctions between the tighter demands of the biomass / carbon monitoring data requirements (the MRV data) of REDD financing instruments, and, the broader flexible information needed to monitor social issues. The design and sustainable operation of monitoring needs to be a collaboration between the outside demands for ‘hard data’, and the rich internal understanding and recognition of local conditions and local priorities.

## Supporting Information

S1 Appendix Documento de Sistematización. (PDF)

S2 Appendix Lecciones aprendidas y buenas prácticas. (PDF)

S3 Appendix Guía participativa Monitoreo Comunitario. (PDF)
