# Supplementary material for: Moving from Measuring, Reporting, Verification (MRV) of Forest Carbon to Community Mapping, Measuring, Monitoring (MMM): Perspectives from Mexico
Source: PLoS One. 2016 Jun 14;11(6):e0146038. doi: 10.1371/journal.pone.0146038 (PMC4907456; doi:10.1371/journal.pone.0146038)
Supplement: S1 Appendix — (PDF) [file pone.0146038.s001.pdf]

2013

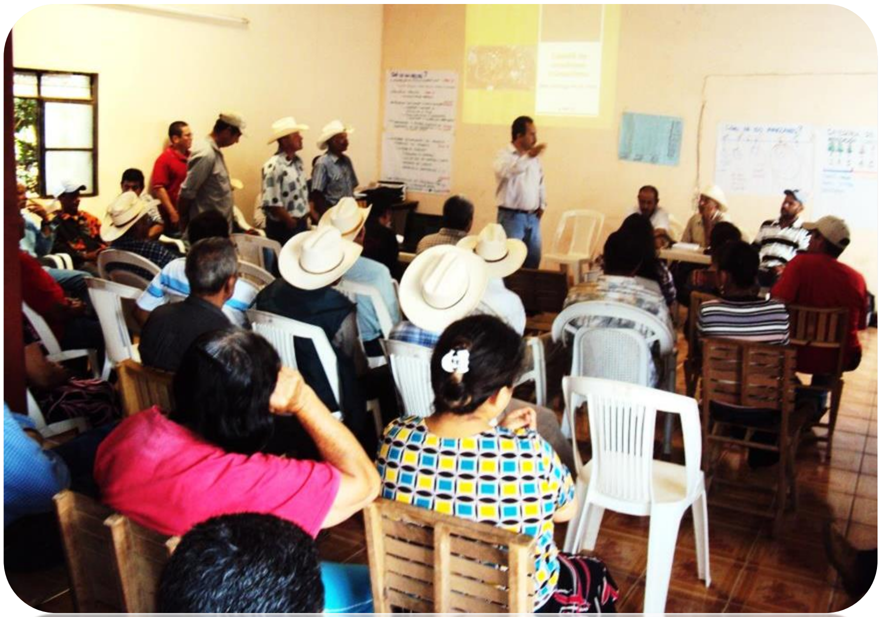

PRODUCTO FINAL

Documento de Sistematización

Acompañamiento del Programa de Monitoreo Comunitario en Áreas Forestales que se encuentran en la Región Cuencas Costeras de Jalisco

Forma parte de uno de los productos finales del proyecto llamado "instrumentación y sistematización de la experiencia del monitoreo Comunitarios de Recursos Naturales en Jalisco" como parte del convenio CGCRB/CONAFOR-AFD/041/2013 / CRB005/ZQ005/13.

incidencia social

incidencia social
Asociación Civil

Noviembre de 2013
Jalisco, México.

DOCUMENTO DE SISTEMATIZACIÓN

* * * * * * * * * *

Acompañamiento y Sistematización del Programa de Monitoreo Comunitario en Áreas Forestales que se encuentran en la Región de Cuencas Costeras de Jalisco.

AFD Agence Française de Développement — SEMARNAT Secretaría de Medio Ambiente y Recursos Naturales — CONAFOR Comisión Nacional Forestal — CONABIO Comisión Nacional para el Conocimiento y Uso de la Biodiversidad

Asociación Civil

Documento de Sistematización

* * * * * *

Acompañamiento y Sistematización del Programa de Monitoreo Comunitario en Áreas Forestales que se encuentran en la Región de Cuencas Costeras de Jalisco

Textos e ideas:
Renato Ravelo Rodríguez, y
Camilo Tlacaelel Simancas Del Águila

Incidencia Social, A.C.
Calle: Altamirano No.15,
Col. Guerrero 200,
Chilpancingo de los Bravo,
Guerrero, México.
Tel.: +52 (747) 11609-44
E-mail: incide.guerrero@gmail.com

CONTENIDO

Introducción 5
- La iniciativa REDD+ 6
- La sistematización 7

Los actores 11
- Las juntas intermunicipales 11
- Los ejidos 14
  - Ejido Barranca del Calabozo 14
  - Ejido El Empedrado 16
  - Ejido Santiago de los Pinos 19
  - Ejido El Jorullo y Anexos 22

Primeros hallazgos 25
- Actividades económicas de los ejidos 25
  - Análisis de la integración de las empresas sociales 26
  - Estructura social 27
  - Capital social y gobernanza local 27
  - Asambleísmo y toma de decisiones 28
  - Seguimiento a procesos 29
  - Roles y equidad 30
  - Grupos de poder excluyentes e incluyentes 30
  - Arreglos, acuerdos y normas 30
  - Acceso a los recursos comunes 31
  - Presión sobre los recursos comunes 31

Actividades realizadas 33
  - Desarrollo de capacidades 35

La experiencia 38

Lecciones aprendidas 48

Recomendaciones 50
- Gubernamentales 50
- Sobre lo local 50

Referencias bibliográficas 51
- Páginas de internet 52
- Legislación mexicana 52

INTRODUCCIÓN

En este documento se presentan los resultados finales del proyecto *Instrumentación y Sistematización de la Experiencia de Monitoreo Comunitario de Recursos Naturales en Jalisco*, que desarrolló la consultoría Incidencia Social A.C., con el auspicio financiero de la Agencia Francesa de Desarrollo a través del Proyecto LAIF (Fondo de Inversión para Latinoamérica) México. La Comisión Nacional Forestal y la Comisión Nacional para el Conocimiento y Uso de la Biodiversidad fueron las dependencias que se encargaron de la operación y administración del proyecto. Se contó asimismo con la participación de Peace Corps U.S., institución que acompañó el proceso en los cuatro ejidos en el estado de Jalisco, México.

México recientemente ha asumido compromisos basados en distintos acuerdos internacionales y ha diseñado estrategias interinstitucionales para abordar integralmente el tema de cambio climático, la reducción en las tasas de deforestación así como degradación, la conservación y aprovechamiento de los recursos mediante el fortalecimiento del manejo sustentable de los recursos por parte de los dueños y poseedores de los bosques y selvas en México.

Estos propósitos colocan a ejidos y comunidades agrarias como agentes principales en la planeación territorial y el diseño de estrategias de uso sustentable de la tierra, pues se parte del principio de que si los integrantes de los núcleos agrarios se integran plenamente en la formulación de las iniciativas que les dan seguimiento, mediante procesos con una clara orientación participativa, se incrementan notablemente las probabilidades de que los impactos, tanto ecológicos como socio económicos, generen los beneficios deseados y les proporcionen las herramientas que se necesitan para cuidar a su propio patrimonio natural.

Los dueños y poseedores de terrenos forestales, principalmente ejidos y bienes comunales, y en menor grado, otros tipos de tenencia como la propiedad privada, históricamente han tomado decisiones para el uso de su territorio que explican el estado en que se encuentran. Las decisiones locales y muy específicas sobre el uso de los recursos y del territorio –sea para su conservación, protección, aprovechamiento o degradación- es importante analizarlas con cierto detalle porque serán clave para explicar la viabilidad –constitución y funcionamiento eficaz- de los comités de monitoreo, que se abordarán más adelante.

El uso que le dan al territorio y a sus recursos naturales los ejidatarios y comuneros obedece por lo general, a usos, costumbres y acuerdos locales que se han construido a lo largo del tiempo. Estas regulaciones de acceso a los recursos, muchas veces favorecen la permanencia de un bosque o una selva, pero otras más, también, han generado la degradación de sus terrenos y del patrimonio común y en última instancia entran en un círculo de pérdida de sus activos ambientales, lo que genera más pobreza.

Otras comunidades agrarias y ejidos en cambio, han fortalecido sus procesos tradicionales de uso y disfrute de sus recursos, logrando incluso procesos de transformación especializados y con organizaciones productivas sólidas y complejas; esta condición partió de algún proceso inicial y fueron adquiriendo capacidades técnicas locales para un manejo y aprovechamiento de sus recursos y posteriormente la transformación más especializada.

Es así que en comunidades con amplia experiencia sobre el manejo de los recursos y su aprovechamiento, tienden a tener bosques más conservados. En contraparte, comunidades que presentan estructuras desgastadas y poco capital social internamente no tienen sólidos acuerdos internos y existe poca participación, y por lo general registran altas tasas de deforestación y degradación de sus recursos naturales.

En este marco referencial se presenta este proyecto que busca la construcción de capacidades técnicas e institucionales a nivel local para la implantación de Acciones Tempranas de Reducción de Emisiones por Deforestación y Degradación (REDD+).

Una de las variables que se busca relacionar, es precisamente la conservación de los recursos, su manejo y aprovechamiento con respecto al grado organizativo, el capital social y las fortalezas de una comunidad en términos de sus estructuras, versus las comunidades desorganizadas y con débiles estructuras, con su grado de deforestación y degradación. A partir de la observación de estas variables se generaron datos muy relevantes acerca de los comités comunitarios de monitoreo.

La iniciativa REDD+

Uno de los propósito de la iniciativa REDD+ es frenar las tasas de deforestación y degradación, para lo cual será necesario tener puestos los reflectores en un aspecto fundamental: las causas y motivos que impulsaron la toma de decisiones de los dueños y poseedores de esos

terrenos forestales para ejercer la presión sobre los recursos y el costo de oportunidad que reflejó la deforestación frente a la conservación o manejo forestal.

En este marco, las comunidades son depositarias de un conocimiento de su entorno, se han asumido parte del mismo, y por tanto han tomado decisiones sobre el territorio sabiéndose dueñas del mismo. La premisa inicial de este trabajo es conocer "si fortaleciendo las capacidades locales basadas en el conocimiento adquirido para valorar y analizar los recursos naturales (inventario forestal y monitoreo) con que cuentan, tiene un impacto directo o indirecto en la decisión de las asambleas sobre la deforestación". Así también, dilucidar si estas nuevas capacidades adquiridas en la medición sistemática aunado al conocimiento de sus recursos (información auto construida), empodera y madura decisiones colectivas en torno a la conservación.

Este trabajo busca resaltar la pertinencia de incluir en los procesos de planeación, diseño e instrumentación que tienen que ver con las comunidades y sus esquemas del uso del territorio y sus recursos, a las instituciones gubernamentales con sus estrategias de fomento a la conservación y manejo forestal así como a las demás instituciones educativas, ONG y consultorías técnicas que inciden en un determinado territorio, para que pueda generarse colectivamente una construcción de modelos acorde a las condiciones locales pero nacionalmente funcionales.

La sistematización

De manera particular, este documento incluye el trabajo de sistematización de la experiencia, como un ejercicio de construcción de un acervo documental y para la generación de saberes y conocimientos críticos desde la práctica. Se buscó que no fuera una mera recopilación de datos o narración de eventos ni tampoco la producción de un informe síntesis de una experiencia, sino más bien, un insumo útil para futuros esfuerzos de monitoreo comunitario a nivel local y también un referente en el diseño, construcción e implementación sobre el tema de Cambio Climático y REDD+, en el componente de Monitoreo, Reporte y Verificación (MRV), al ser un documento que pone énfasis en la necesidad de fomentar la construcción de capacidades, el involucramiento y apropiación del proceso de los actores locales para la instalación de un sistema de MRV como referente local, regional y nacional.

El presente texto también considera una sección de lecciones aprendidas que buscan contribuir a la conceptualización de un sistema de MRV sub-nacional abordada desde un enfoque principalmente social pero retomando los elementos técnico metodológicos fundamentales a considerarse para el trabajo en el andamiaje local.

El proyecto se desarrolló entre los meses de mayo y octubre. Los actores centrales fueron los ejidatarios de cuatro núcleos agrarios cuyos núcleos agrarios que se localizan en el estado de Jalisco,[1] entidad piloto seleccionada por los altos índices de deforestación que presenta y con la intención de correr un ejercicio de gobernanza local, basado en las figuras de Juntas Intermunicipales como instrumento de planeación, gestión y manejo integral del territorio a partir de la cuenca.

Como parte de la identificación de aspectos aun no desarrollados dentro del sistema de Medición, Reporte y Verificación, se determinó poner en marcha la integración y consolidación de "comités pilotos de monitoreo comunitarios", a través de un proceso participativo e incluyente, en el que sean, los mismos dueños y poseedores de las tierras ejidales, quienes se involucren en ello.

El criterio de selección utilizado fue proponer núcleos agrarios que reflejaran un tejido social relativamente fuerte, basados en su proceso económico productivo, ligado al uso y aprovechamiento de los recursos naturales. Las propuestas de ejidos fueron emitidas por dos juntas intermunicipales: la Junta Intermunicipal para la Gestión Integral del Medio Ambiente de la Sierra Occidental y Costa (JISOC), y la junta intermunicipal de la Cuenca del Río Coahuayana (JIRCO), así como de la misma Comisión Nacional Forestal:

- Ejido El Jorullo y Anexos, municipio de Puerto Vallarta.
- Ejido Santiago de los Pinos, municipio de San Sebastián del Oeste.
- Ejido El Empedrado, municipio de Mascota.
- Ejido Barranca del Calabozo, municipios de Pihuamo, Tecatitlán y Tuxpan.

Los ejidos, luego de que se hizo la promoción, se interesaron en diferentes medidas en el proceso de monitoreo comunitario y eligieron temáticas diferentes en relación con sus recursos naturales, a saber: manejo forestal, plagas forestales y su grado de afectación en el predio, así como el monitoreo del recurso hídrico. Como se comentará más

[1] Región de los municipios de la Junta Intermunicipal de Medio Ambiente para la Gestión Integral de Sierra Occidental Costa (JISOC) y la Junta Intermunicipal de Medio para la Gestión Integral del Río Coahuayana (JIRCO).

adelante, al final sólo se concluyó el proceso en tres de esos núcleos agrarios.

El solo interés o disposición, aunque importantes para iniciar este tipo de procesos, no son factores suficientes para alcanzar resultados positivos. La esencia de este trabajo de sistematización es precisamente escudriñar, con base en el trabajo de campo y de contacto con los ejidatarios, aquellas circunstancias y elementos que pueden obstaculizar, favorecer o facilitar un proceso de monitoreo comunitario, sobre todo la integración y funcionamiento de comités.

Uno de los indicadores clave para hacer el análisis es sin duda el nivel de organización interna que presentó cada núcleo agrario; se propusieron para este fin diferentes sub-indicadores que pueden mostrar las fortalezas y debilidades en este aspecto: formas de organización social, usos y costumbres, reglamentación interna y su aplicación, asambleísmo y capital social esquema de gobernanza local.

Otro indicador importante se refiere al grado de desarrollo económico-empresarial de cada núcleo agrario a partir del manejo, uso y aprovechamiento de los recursos naturales con que cuentan. En este punto se observaron aspectos como la tipología de productores, la participación de no ejidatarios en las empresas sociales, la distribución de las utilidades de las empresas, entre otros.

También se observó, como otros de los indicadores, el desarrollo de capacidades que han tenido los ejidatarios de los diferentes núcleos agrarios. En este aspecto fue interesante observar el efecto que han tenido los diferentes eventos orientados a la capacitación de los ejidatarios promovidos y aplicados por la Comisión Nacional Forestal en estos núcleos agrarios, la relación con los prestadores de servicios profesionales, entre otros.

Sin duda, el caso del ejido el Empedrado, municipio de Mascota, en el que no se logró concluir con la instrumentación del comité de monitoreo ni con su validación social, proporcionó datos relevantes y una rica experiencia para el estudio, pues aportó elemento necesarios para la reflexión y que nos permitirá considerar el involucramiento de diversos actores externos en los ejidos y comunidades en los que se pretenda realizar este tipo de trabajos.

Para realizar el trabajo de sistematización y aprendizaje en los cuatro ejidos seleccionados se consideraron los siguientes objetivos:

General:

- Sistematizar la experiencia del proceso de fortalecimiento institucional y de capacidades técnicas a través de la instalación y consolidación de comités de monitoreo comunitario en áreas forestales, así como documentar las lecciones aprendidas con cuatro ejidos piloto, bajo un esquema de gobernanza local intermunicipal en Jalisco, México, como parte de las acciones tempranas de REDD+.

Específicos:

- Facilitar a los ejidos pilotos que se encuentran en las Cuencas Costeras de Jalisco las herramientas técnicas y metodológicas necesarias para instalar un sistema de monitoreo comunitario en la región de cuencas costeras de Jalisco.
- Contribuir a la preparación organizativa y técnica de los comités de monitoreo para mejorar el diseño y la implementación preliminar de un sistema de monitoreo de recursos forestales dentro de los territorios de los ejidos.
- Sistematizar el proceso de creación, operación y evaluación de los comités de monitoreo a efecto de obtener una herramienta útil para futuros esfuerzos de monitoreo comunitario a nivel local.
- Obtener lecciones aprendidas para guiar la conceptualización de un sistema MRV sub-nacional.
- Caracterizar de manera general la dinámica local considerando lo ambiental, social, cultural y económico de cada núcleo agrario involucrado.

El monitoreo comunitario de los recursos naturales es una herramienta que puede contribuir al fortalecimiento de los núcleos agrarios, porque es un proceso en el que los ejidatarios se apropian de un concepto que tiene mucho significado a la hora de la toma de decisiones, como es la vigilancia y el cuidado de los recursos. El monitoreo hecho por los propios ejidatarios (as), si bien es complejo porque requiere del desarrollo de capacidades muy especializadas y un buen nivel de organización en los ejidos, implica también un proceso de sensibilización y un alto grado de autogestión.

Para lograr comités de monitoreo fuertes y eficaces se requirió de una serie de pasos que en este documento hemos tratado de sistematizar. El involucramiento de los ejidatarios y sus autoridades en el proceso fue clave para lograrlo, pues sostenemos que si no se promueve la participación al interior de los núcleos agrarios, cualquier iniciativa de desarrollo estará condenada al fracaso.

LOS ACTORES

Las juntas intermunicipales

En el marco del modelo de gobernanza de las juntas intermunicipales de medio ambiente, creadas con la figura de organismo público descentralizado, nació JIRA, la cual fue creada inicialmente para resolver un problema compartido, concreto, creciente, con aristas diversas, basado en el territorio. Este esfuerzo dio frutos con el principio de "la unión hace la fuerza" y presenta las siguientes características:

- Una fuerte presencia e incidencia en términos político territoriales, con zonas de influencia en el área total de los municipios asociados.
- Fue creada con la intención de resolver un problema ambiental común muy concreto, que impactaba en el ámbito de la salud, económico y político.
- Lograron concurrencia de los tres órdenes de gobierno para atender la problemática con diversos sectores temáticos del gobierno (desarrollo rural, medioambiente, salud, etc.)
- Un principio intrínseco para atender la problemática en el territorio, fue la visión de cuenca. Con esta base lograron que amplios sectores gubernamentales incidieran a partir de la planeación y desarrollo para intervenir con recursos sobre el territorio para resolver la problemática concreta.
- Con esta experiencia, se pusieron en la mesa de discusión diversos temas paralelos que tenían que resolverse como cultura y educación ambiental, así como otras problemáticas que por coyuntura se pusieron en el tintero para poder dar seguimiento y aprovechar la plataforma del OPD (Organismo Público Descentralizado)
- El ejemplo de la JIRA como modelo para atender el tema de manejo integrado de la cuenca como una entidad que involucra a los tres órdenes de gobierno, los cuales organizan, planean, promueven y gestionan procesos de desarrollo a partir de las necesidades concretas del territorio es innegable. La replicabilidad del modelo estará sujeta a encontrar los elementos que detonen la alianza para resolver problemas concretos y sortear la voluntad política y los factores de corta temporalidad de los presidentes municipales quienes tienen gran peso en las decisiones de las Juntas. Esta condición aunada a la planeación de corto plazo que tienen los municipios y su visión de largo plazo pesa menos que la de corto plazo (enmarcado prácticamente por el periodo de

gobierno municipal) está justificado por los compromisos de campaña política.

- Actualmente la JIRA, como está integrada por un conjunto de municipios que comparten una misma cuenca, le permite atender de forma coordinada las agendas de mitigación y adaptación al cambio climático y los co-beneficios de conservación de la biodiversidad y mejoramiento en el abastecimiento y calidad del agua.

Aunado a esto, la iniciativa para replicar la metodología del modelo de gobernanza intermunicipal en otras áreas, en su creación, el Gobierno Federal, busca agregar la implementación de mecanismos REDD+ a nivel local, buscando tomar las experiencias aprendidas de la JIRA creando otros modelos de gobernanza en regiones prioritarias para la implementación de REDD+. Entre las actividades que desarrollan para ellos ha sido el desarrollo de talleres, entrevistas y otros procesos consultivos y de identificación de elementos de replicación.

La región Costera de Jalisco, se caracteriza por tener una gran variedad de ecosistemas naturales y por presentar una alta diversidad biológica. Incluye diez Áreas Naturales Protegidas para la conservación del jaguar y una de las regiones de mayor importancia para la conservación de aves migratorias de América del Norte. Provee de agua a todo el estado de Colima y al desarrollo turístico de la Costalegre de Jalisco incluyendo a la ciudad de Puerto Vallarta. Incluye también importantes áreas bajo manejo y aprovechamiento forestal maderable y no maderable.

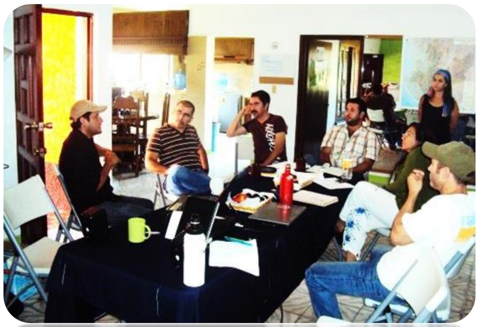

En esta misma región, se trabajó, además de la JIRA, con dos juntas intermunicipales más: la del Río Coahuayana (JIRCO) que integra a 12 municipios, y la de la Sierra Occidental-Costa (JISOC) que se forma por siete municipios. Las tres forman parte de la región “Cuencas Costeras de Jalisco”.

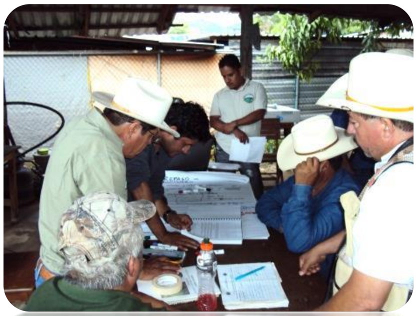

De acuerdo con las fichas técnicas proporcionadas por la Secretaría de Medio Ambiente y Desarrollo Territorial del gobierno de Jalisco, la JISOC y JIRCO son asociaciones de municipios constituidas como Organismos

Públicos Descentralizados Intermunicipales, creados con el objeto de dar apoyo técnico a los municipios integrantes para la elaboración, gestión e implementación de los proyectos y programas relacionados con el medio ambiente y manejo de recursos naturales de aplicación en sus territorios sobre los temas de Ordenamiento ecológico del territorio, ordenamiento urbano, impacto ambiental, restauración ecológica, creación y manejo de áreas naturales protegidas de carácter municipal, manejo y protección de bosques, información ambiental a la ciudadanía, educación ambiental, mejoramiento de la prestación de los servicios públicos municipales y todas las áreas relacionadas con el medio ambiente que sean de interés de los municipios.

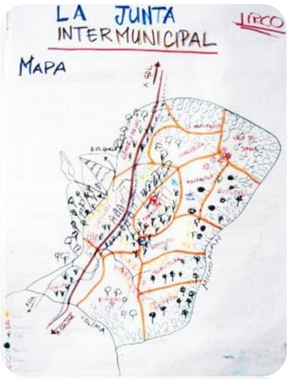

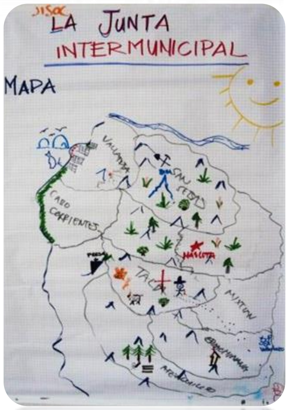

En el caso de JISOC, los ayuntamientos municipales considerados son Atenguillo, Cabo Corrientes, Guachinango, Mascota, Mixtlán, San Sebastián del Oeste y Talpa de Allende. Mientras que para JIRCO, los ayuntamientos municipales participantes son Concepción de Buenos Aires, Gómez Farías, Mazamitla, Pihuamo, Quitupán, Tamazula de Gordiano, Tecalitlán, Tonila, Tuxpán, Valle de Juárez, Zapotiltic y Zapotlán el Grande.

La acción temprana REDD+ contempla dos ejes principales:

a) Integración de políticas públicas y gobernanza para la mitigación y adaptación al cambio climático, enfocadas a detener las causas de deforestación y degradación forestal
b) Desarrollo de un sistema de Medición, Reporte y Verificación (MRV) de las emisiones de carbono forestal.

La experiencia inició en 2010 y se continúa fortaleciendo mediante diversos instrumentos, estos se plantean en tres fases:

1) Fortalecimiento institucional a través de la consolidación de otras asociaciones intermunicipales, después de JIRCO y JISOC.
2) Elaboración de estrategias locales REDD+, para fortalecer la estrategia de mitigación y adaptación al Cambio Climático.
3) Desarrollo de actividades de desarrollo rural sustentable acorde a las adecuaciones al POA.

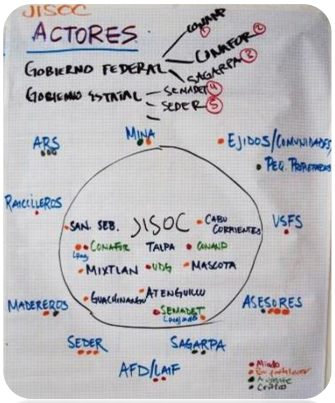

La apuesta es que las asociaciones de municipios funjan como agentes técnicos encargados de implementar los programas de SEMARNAT–CONAFOR y SAGARPA, así como los programas de Secretaria de Desarrollo Rural (SEDER) y la Secretaría de Medio Ambiente para el Desarrollo Sustentable (SEMADES) del gobierno de Jalisco. Será esta acción temprana REDD+ parte del proceso de fortalecimiento de capacidades de gobiernos locales para instrumentar políticas y programas de conservación y desarrollo rural sustentable.

Los Ejidos

Ejido Barranca del Calabozo

Datos generales. La superficie del núcleo agrario es de 1,809.06 hectáreas; su resolución presidencial data del 6 de febrero de 1968, publicada en el DOF el 4 de abril del mismo año. El número de personas que cuenta con derechos agrarios es de 42 ejidatarios.

Son dos los asentamientos humanos del ejido: San José del Tule y San Isidro, mientras que la distribución de los seis polígonos se localizan en los municipios de Pihuamo, Tecatitlán y Tuxpan, de la región "sureste y sur", del estado de Jalisco.

De acuerdo con el INEGI, Barranca del Calabozo tiene una población de 470 personas que viven en ambas localidades, 243 de las cuales son mujeres, y 227 hombres.

Asambleísmo. El ejido convoca a asambleas generales de ejidatarios el primer domingo de cada mes, acuden a éstas 38 ejidatarios en promedio. Frecuencia y asistencia se observaron en las asambleas de este núcleo agrario, lo que significa que hay un activo asambleísmo y por lo tanto un gran potencial de desarrollo.

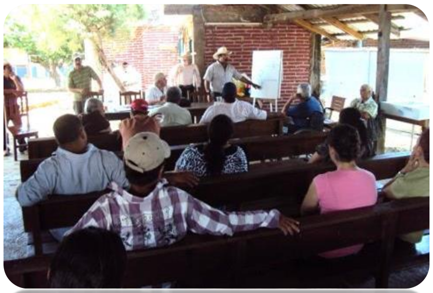

Cuando hay elección de los órganos de representación y vigilancia acuden todos los ejidatarios, síntoma de un interés grande por

lo que sucede en el ejido. Las asambleas se celebran en el aserradero, en un espacio específico, que ocupan también para impartir algunos talleres y cursos de los seminarios de comunidad a comunidad.

Cada año se reúnen para el reparto de utilidades de las empresas del ejido en asamblea general, en éstas también acuerdan los montos para reinvertir en la cadena productiva de su empresa, y destinan una parte para cubrir los impuestos que deben pagar a los Ayuntamientos (existe una ley estatal de catastro).

Empresa social. El núcleo agrario de Barranca del Calabozo es considerado como punta de lanza en el aprovechamiento sustentable de los bosques, ya que se han apropiado de todo el proceso (desde el mismo aprovechamiento hasta la elaboración de muebles de madera, pasando por el proceso del aserradero). Se constituyó como una empresa social para generar empleos con el núcleo de población ejidal y mejorar la calidad de vida de los mismos, este proceso fue posible por la participación y gestión de apoyos ante el Programa de Silvicultura Comunitaria de la Comisión Nacional Forestal.

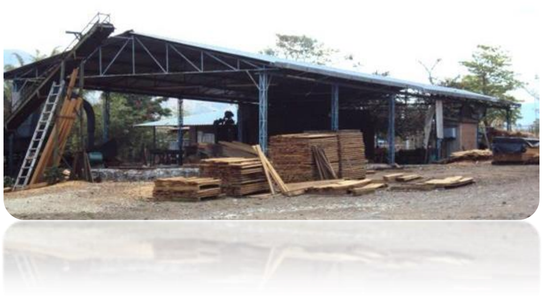

El ejido tiene la capacidad de brindar servicios como el seminario de comunidad a comunidad y acompañamiento, seguimiento y capacitación para la instalación y operación de aserraderos, uso de grúa para la extracción de los árboles derribados, entre otros.

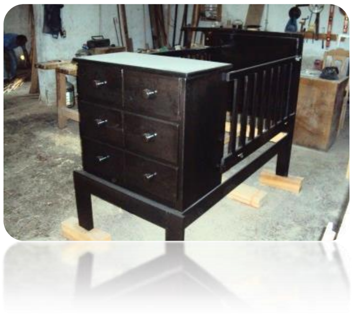

En el levantamiento de datos para el control del aprovechamiento maderable del ejido, sólo dos ejidatarios se involucran porque viven cercanos a los rodales fraccionados y estudiados, y son avecindados e hijos de ejidatarios los que se involucran directamente en el control del aprovechamiento, el cual fue elaborado con el Sistema de Conservación y Desarrollo Silvícola.

En el ejido se observan diferentes opiniones, acuerdos y desacuerdos, esto fue verificado mediante la aplicación de un ejercicio o dinámica para valorar la comunicación, coordinación, y organización de los integrantes del comité de monitoreo comunitario, cuyos comentarios aludieron a que realmente les hace falta organización, comunicación y

coordinación, pero que no es algo que esté fuera de sus perspectivas, sobre todo cuando tienen un proceso de desarrollo forestal comunitario.

Asesoría Técnica. Fundamentalmente se ha buscado el apoyo en diversos conceptos ante la Comisión Nacional Forestal, tanto al Programa de Desarrollo Forestal Comunitario como del Desarrollo de la Cadena Productiva Forestal como se describe a continuación:

PROYECTOS DEL EJIDO BARRANCA DEL CALABOZO EN EL 2013 A TRAVÉS DE LOS PROGRAMAS DE LA CONAFOR

Concepto de Apoyo	Modalidad de Apoyo	Monto de Apoyo
FC1. **Fortalecimiento del Capital Social y Humano**	FC1.4 Estudio de Ordenamiento Territorial Comunitario	$41,200.^{00}
	FC1.6 Seminario de Comunidad a Comunidad	$58,500.^{00}
	FC1.9.1 Talleres y Cursos de Capacitación a Productores Forestales	$23,200.^{00}
FC2. **Desarrollo de Capacidades de Gestión**	FC2.5 Asesoría para Ejidos y Comunidades con Actividad Empresarial	$60,000.^{00}
CP1.3 **Estudios Técnicos, de Factibilidad, Plan de Negocios y Proyecto de Certificación**	CP1.3 Estudios Técnicos, de Factibilidad, Plan de Negocios y Proyecto de Certificación	$70,000.^{00}

El Ejido Barranca del Calabozo está considerado, según la tipología de la Conafor, como productores con capacidad de transformación y comercialización (tipo IV), porque disponen de infraestructura para transformar bienes y servicios en productos y subproductos terminados para su comercialización directa en los mercados.

Ejido El Empedrado

Datos generales. El núcleo agrario cuenta con una superficie de 6,780 hectáreas, su resolución presidencial es del 7 de mayo de 1958, publicada el 18 de agosto del mismo año en el DOF. San Miguel de Tovar es el único asentamiento humano, y éste se localiza en el municipio de Mascota, en la región “sierra occidental” del estado de Jalisco.

La población total suma 208 personas, de las cuales 55 por ciento son mujeres y 45 por ciento hombres. El núcleo agrario cuenta con 31 ejidatarios legalmente reconocidos.

Las festividades del núcleo agrario son: el 29 de septiembre, en honor a San Miguel Arcángel, y el 16 de octubre en honor a Santa Eduviges.

En el territorio ejidal se presentan problemas como basura, cacería furtiva, tala clandestina, escasez y contaminación del agua, baja productividad en los suelos de cultivos, incendios forestales, caminos en mal estado, falta de operatividad del comité de vigilancia en el bosque, falta de organización en las tareas comunitarias, falta de participación en asambleas general de ejidatarios, los responsables de la empresa forestal comunitaria no informan a la asamblea de los ingresos y egresos.

Asambleísmo. En el ejido se realizan asambleas en determinadas fechas acudiendo puntualmente alguno ejidatarios, en ellas se resuelven prácticamente problemáticas locales en la que se ven involucrados tanto ejidatarios como avecindados.

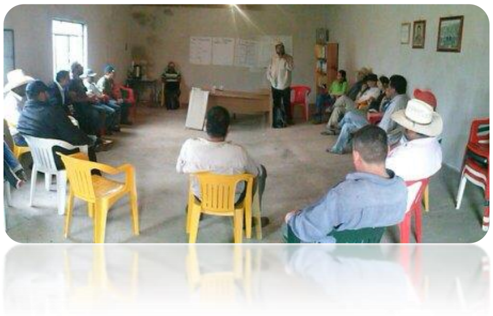

Solo cuando hay elecciones acuden puntualmente más de la mitad para tomar el acuerdo de quién los representara durante tres años que dura el encargo del comisariado y consejo de vigilancia.

La reciente creación de la empresa social comunitaria no ha permitido que se realice una asamblea para determinar el reparto de las utilidades o bien su reinversión, tampoco ha habido disponibilidad de los encargados de la empresa del ejido en querer celebrar una asamblea para informar (rendir cuentas) de las problemáticas en las que se envuelve el aserradero.

Para la elaboración de actas de asamblea dependen mucho del visitador agrario y su asesor técnico.

Empresa social. El ejido refleja un avance en cuanto a la iniciativa de una empresa forestal comunitaria, porque ya se está ejecutando un aprovechamiento forestal maderable en una superficie de 2,828-38-00 hectáreas.

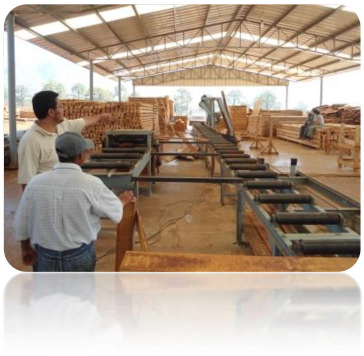

El ejido Barranca del Calabozo capacitó a los ejidatarios para la puesta en marcha del aserradero que hasta la fecha funciona, pero hay deficiencias en la maquinaria y sobre todo el conocimiento de los locales para solucionar los problemas que se presentan al respecto.

El ejido es beneficiario del pago por Servicios Ambientales Hidrológicos, por lo que atienden y realizar actividades concernientes a mejorar estos servicios, sin embargo el área se encuentra en riesgo por la plaga de muérdago que avanza 650 metros cada año.

La población no se involucra, sobre todo los jóvenes, en las actividades comunitarias como en el apoyo de Servicios Ambiental Hidrológicos ni en la cadena de la empresa, lo cual propicia la dependencia hacia los asesores técnicos externos al ejido.

Asesoría Técnica. El ejido ha gestionado los siguientes conceptos de apoyo ante la Comisión Nacional Forestal:

PROYECTOS DEL EJIDO EL EMPEDRADO

Año	Concepto de apoyo	Programa
2012	Estudio de ordenamiento territorial	Programa de Desarrollo Forestal Comunitario
2012	Seminario de comunidad a comunidad	Programa de Desarrollo Forestal Comunitario
2012	Talleres y curso de capacitación a productores forestales	Programa de Desarrollo Forestal Comunitario
2012	Asesoría para el fortalecimiento de empresas forestales comunitarias	Programa de Desarrollo Forestal Comunitario
2012	Apoyo a la industrialización	Desarrollo de la Cadena Productiva
2012	Promotor forestal comunitario local	Programa Especial Cuencas Costeras en el Estado de Jalisco

PROYECTOS DEL EJIDO EL EMPEDRADO QUE HAN SIDO REASIGNADOS A TRAVÉS DE LOS PROGRAMAS DE LA CONAFOR

Año	Concepto de apoyo	Programa
2012	Pago de servicios ambientales	Programa Especial Cuencas Costeras en el Estado de Jalisco

Los apoyos recientes fueron los siguientes:

PROYECTOS DEL EJIDO EL EMPEDRADO QUE SE LLEVARAN A CABO EN EL 2013 A TRAVÉS DE LOS PROGRAMAS DE LA CONAFOR

Concepto de Apoyo	Modalidad de Apoyo	Monto de Apoyo
**FC1.Fortalecimiento del Capital Social y Humano**	FC1.5 Comité de Vigilancia Participativa	$39,000.00
	FC1.6 Seminario de Comunidad a Comunidad	$58,500.00
	FC1.8 Promotor Forestal Comunitario	$31,500.00
	FC1.9.1 Talleres y Cursos de Capacitación	$23,200.00
**FC2. Desarrollo de Capacidades de Gestión**	FC2.3 Estudios técnicos especializados para el establecimiento de áreas de alto valor para la conservación comunitaria	$75,000.00
**A1 Estudios Forestales**	A1.2 Programa de manejo forestal maderable	$56,928.00

**A3 Certificación**	A3.3 Otras certificaciones, acreditación y/o Acompañamiento	$70,000.00

Según la tipología de la Comisión Nacional Forestal, a los ejidatarios del Empedrado se les considera como productores con capacidad de transformación y comercialización (tipo IV), porque disponen de infraestructura para transformar bienes y servicios en productos y subproductos terminados para su comercialización directa en los mercados.

Ejido Santiago de los Pinos

Datos generales. La resolución presidencial del 21 de junio de 1939, publicada en el DOF el 29 de agosto de 1942, dota a este ejido de una superficie de 8,114 hectáreas. Tiene tres poblados (Santiago de los Pinos, Real Alto de Oxtotipac y San Sebastián del Oeste), y se localiza en el municipio de San Sebastián del Oeste, en la región "sierra occidental" del estado de Jalisco. De acuerdo con el ordenamiento territorial comunitario, la superficie del ejido está distribuida de la siguiente manera:

DISTRIBUCIÓN DEL TERRITORIO EJIDAL SEGÚN
EL ESTUDIO DE ORDENAMIENTO TERRITORIAL COMUNITARIO

Uso actual del suelo	Total Ha.	%
Producción forestal maderable	1,116.92	12.68
Conservación del bosque	3,843.69	41.76
Bordo	0.54	0.01
Área degradada	136.61	1.48
Agrícola	1,756.22	19.08
Pastizal	283.97	3.09
Arbustos	13.00	0.14
Roquerías	21.67	0.24
Renuevo	20.08	0.22
Fruticultura	6.26	0.07
Minería	12.48	0.14
Superficie no estudiada	1,867.51	20.29
Núcleo ejidal	71.37	0.78
Unidad de manejo para la conservación de la vida silvestre UMA	3.05	0.03
Vivero forestal	0.26	0.00

De acuerdo con los indicadores del Censo de Población y Vivienda 2010 del INEGI, hay una población total de 1,271 personas, de las cuales 51 por ciento son hombres y 49 por ciento mujeres. El número de personas que cuenta con derechos agrarios dentro del territorio ejidal es de 35 ejidatarios.

Asambleísmo y capital social. La gente se organiza para realizar varias festividades, pero la más importante es el 20 de noviembre en honor al Santo Santiago. En este tipo de eventos se puede observar el capital social que tiene el ejido, pues aún se pueden observar una marcada identidad, además de lazos de confianza entre los habitantes. La festividad que se comenta se organiza en asamblea general, lo cual expresa la importancia que tiene para el pueblo; se elige a un comité de fiestas que se encarga de organizar el festejo, con la cooperación y el apoyo de todos los vecinos y las autoridades locales. Durante el evento, hay peregrinación, se lanzan cohetes de pólvora y al terminar de misa ofrecen una comida en la plaza principal amenizando el mariachi, por la noche se realiza el baile.

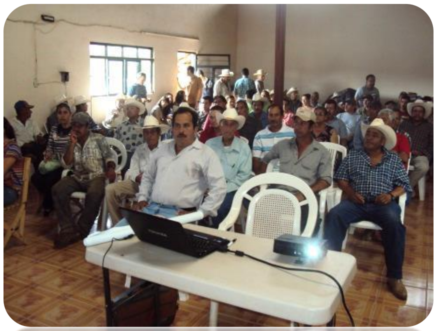

Las asambleas se celebran regularmente el primer domingo de cada mes, convocadas por el comisariado ejidal. Como se sabe, la asamblea general es el máximo órgano de toma de decisiones del núcleo agrario; además del comisariado ejidal y del consejo de vigilancia, también existe la figura del agente municipal, regido por la Ley Orgánica del Municipio Libre del Estado de Jalisco. El agente municipal es el puente de comunicación entre el ayuntamiento y los ciudadanos de las localidades, en ocasiones esta autoridad auxiliar municipal, nombrada por el pueblo, tiene vínculos con las autoridades del ejido. Sin embargo estos agentes tienen un vínculo más estrecho con los comités que se integran de acuerdo a las necesidades locales.

Aprovechamiento forestal. El ejido ha tenido un proceso paulatino respecto del manejo, cuidado, uso y aprovechamiento de sus recursos naturales. El 9 de diciembre de 1996 se efectuó un estudio forestal maderable, que fue aprobado por la SEMARNAT, y renovado el 17 de diciembre de 2008. En el 2012, (19 de enero) se registró el Programa de Manejo para la Conservación de la Vida Silvestre (UMA) SEMARNAT-UMA-IN-360-JAL. Pero los ejidatarios han dejado de aprovechar, desde hace más de 4 años, el volumen de madera que les autoriza el programa de manejo, ya que se ha convertido en un problema muy grave la presencia de una plaga en los pinos (muérdago).

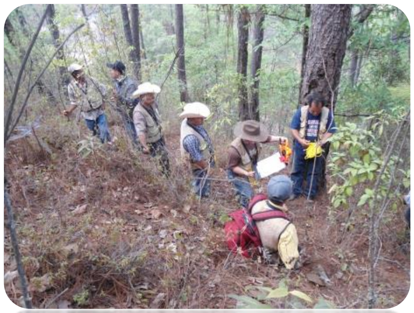

En 2012 solicitaron al Programa de Desarrollo Forestal Comunitario de la Comisión Nacional Forestal, apoyo para la creación de un comité de vigilancia participativa, con la finalidad de promover la participación social de forma organizada, informada, transparente y responsable al interior del núcleo agrario. Dicho comité quedo integrado por 10 personas, fue avalado por la asamblea general de ejidatarios y acreditado en la Procuraduría Federal de Protección al Ambiente.

Asesoría Técnica. Algunos antecedentes del ejido, en cuanto a las solicitudes de apoyo a los diferentes programas de Comisión Nacional Forestal son los siguientes:

PROYECTOS DEL EJIDO SANTIAGO DE LOS PINOS

Año	Concepto de apoyo	Programa
2012	Estudio de Ordenamiento Territorial Comunitario	Programa Especial Cuencas Costeras en el Estado de Jalisco
2012	Comité de Vigilancia Ambiental Participativa	Programa de Desarrollo Forestal Comunitario
2012	Promotor Forestal Comunitario	Programa de Desarrollo Forestal Comunitario
2012	Talleres Didácticos de Educación Ambiental	Programa de Desarrollo Forestal Comunitario
2012	Cultivo Forestal en Aprovechamientos Maderables	Programa Especial Cuencas Costeras en el Estado de Jalisco

En el caso de 2013, se solicitaron y ejecutaron los siguientes apoyos:

Concepto de Apoyo	Modalidad de Apoyo	Monto de Apoyo
**FC1.** Fortalecimiento del Capital Social y Humano	FC1.6 Seminario de Comunidad a Comunidad	$66,300.^{00}$
**FC2.** Desarrollo de Capacidades de Gestión	FC2.6 Talleres para la Constitución de Empresas Forestales Comunitarias 2013	$44,200.^{00}$
**CP1.2** Acta Constitutiva 2013	CP1.2 Acta Constitutiva 2013	$9,680.^{00}$

El *Ejido Santiago de los Pinos* está considerado, según la clasificación de la Comisión Nacional Forestal, como productores que venden en pie, clasificado como de tipo II, porque están autorizados para el aprovechamiento de bienes y servicios en los que éste se realiza por parte de terceros mediante contrato de compra-venta, sin que el propietario o poseedor participe en alguna fase del proceso de productivo.

Ejido El Jorullo y Anexos

Datos generales. Tiene una superficie de 13,767 hectáreas; la resolución presidencial data del 27 de marzo de 1940, siendo publicada el 16 de agosto de 1940 en el DOF. Es un núcleo agrario en el que se localizan los poblados de *Agua Zarca, Guácimas, El Hundido, El Jorullo, El Almacén (Los Almacenes), La Joyita, El Nogalito, Vallejo, Aguacatera, La Arenita, Los Llanitos, San Pedro, Las Higueras del Hundido, La Horta, el Vergel, y Cruz Ignacio*. Hay un padrón de 180 ejidatarios legalmente reconocidos. La municipalidad a la cual pertenecen dichas localidades es la de Puerto Vallarta, en la región "sierra occidental" del estado de Jalisco.

De acuerdo con los indicadores del Censo de Población y Vivienda 2010 del INEGI, hay una población total de 434 personas en trece de las dieciséis localidades, la mayor parte son hombres (54 por ciento) y el resto mujeres (56 por ciento). El número de personas que cuenta con derechos agrarios es de 155 ejidatarios.

La empresa social. Entre las actividades de su proceso de desarrollo, el ejido constituyó una empresa de ecoturismo el 13 de abril del 2005, la Sociedad Cooperativa Sierra Madre Canopy Tour, S.C. de R.L.; en el 2011, recibieron por parte de la consultoría Servicios Agroforestales de Occidente, S.C., asesoría para el fortalecimiento de empresas forestales comunitarias en el que abordaron temas como tipos de gerencia, organigrama funcional de la empresa, tipos y funciones del gerente, y liderazgo, así como la atención al cliente de los servicios que se brindan

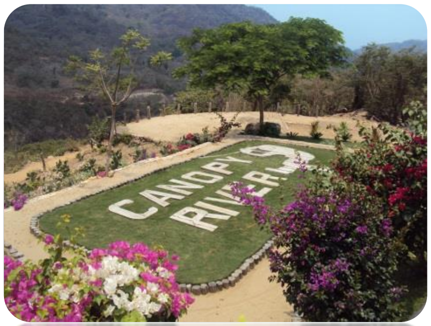

La empresa, sin embargo, fue constituida sólo con las personas que pudieron hacer una aportación en el momento de su conformación, por lo que quedaron ejidatarios fuera por la falta de recursos económicos y otros por falta de iniciativa, según comentarios de ejidatarios. Esto creó una diferencia dentro del núcleo de población, lo que puede ser un factor preponderante para detonar procesos colectivos de beneficios tangibles e intangibles, como lo mencionan en el informe final del taller participativo de servicios ambientales.

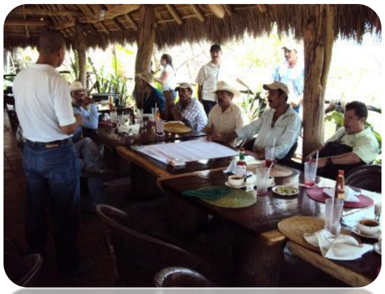

En el 2011, también crearon un comité de vigilancia participativa, asesorado por Servicios Agroforestales de Occidente, S.C., donde sólo ocho personas, de un universo de 180 ejidatarios decidieron involucrarse integrándose al comité y a la vez incorporarse a la red vigía que promueve la Profepa. La consultoría recomendó al comité tomar en cuenta la orientación de crecimiento que tendrá el centro ecoturístico, para prevenir con acciones de planteamientos ambientales y arremeter posibles problemas derivados de paulatino crecimiento.

Pese al poco conocimiento de actores que intervienen con el desarrollo comunitario al interior del ejido, también están presentes las consultorías que apoyan con la ejecución de los conceptos de apoyo que solicitan a la Comisión Nacional Forestal y otros programas que se encuentran al interior de esta institución tanto estatal como la federal.

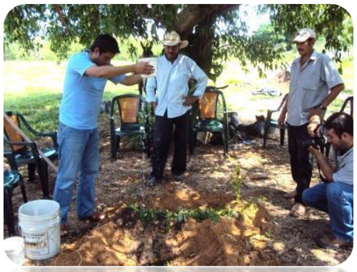

La necesidad de abatir la problemática relacionada con la escases del agua lleva al mismo comité de vigilancia participativa (comité de monitoreo comunitario) a tomar acciones para ello. En el comité de monitoreo se sumó una persona de sexo femenino, cuya disponibilidad por realizar acciones que contribuyan a mejorar las condiciones del ejido, permitió que ocupara la representación del grupo de ejidatarios para el caso del monitoreo comunitario.

Asesoría Técnica. Algunos de los antecedentes del ejido, en cuanto a las solicitudes de apoyo a los diferentes programas de Comisión Nacional Forestal son los siguientes:

PROYECTOS DEL EJIDO EL JORULLO Y ANEXOS

Año	Concepto de apoyo	Programa
2012	Talleres participativos de Servicios ambientales	Programa de Desarrollo Forestal Comunitario
2012	Talleres y cursos de capacitación a productores forestales	Programa de Desarrollo Forestal Comunitario
2012	Promotor forestal comunitario local	Programa Especial Cuencas Costeras en el Estado de Jalisco
2012	Programa PREDIAL de desarrollo forestal de mediano plazo	Programa Especial Cuencas Costeras en el Estado de Jalisco

Para el seguimiento en el desarrollo comunitario del *Ejido El Jorullo y Anexos,* los asesores técnicos, realizaron las gestiones de diversos conceptos de apoyo a la Comisión Nacional Forestal, al Programa de Desarrollo Forestal Comunitario, siendo aprobados los siguientes:

PROYECTOS DEL EJIDO EL JORULLO Y ANEXOS

Concepto de Apoyo	Modalidad de Apoyo	Monto de Apoyo
FC1. **Fortalecimiento del Capital Social y Humano**	FC1.8 Promotor Forestal Comunitario 2013	\$31,500.00
	FC1.9.1 Talleres y Cursos de Capacitación a Productores Forestales	\$23,200.00
FC2. **Desarrollo de Capacidades de Gestión**	FC2.4 Estudios técnicos especializados para la recuperación de áreas degradas por disturbios y/o perturbaciones antropogénicas 2013	\$39,000.00

El *Ejido El Jorullo y Anexos,* está considerado según la clasificación de la Comisión Nacional Forestal como productores con capacidad de transformación y comercialización, de tipo IV, porque disponen de infraestructura para transformar bienes y servicios en productos y subproductos terminados para su comercialización directa en los mercados.

PRIMEROS HALLAZGOS

Actividades económicas de los ejidos

Las dinámicas de los ejidos están definidas por la actividad económica que realizan; el trabajo agrícola, pecuario y en menor medida el forestal, son las actividades productivas que sobresalen en esta región, aunque de manera marginal, uno de los ejidos realiza actividades en el sector terciario de servicios, porque operan un proyecto de ecoturismo en el ejido El Jorullo y Anexos. Por lo tanto, el sector primario, como en la mayoría de los núcleos agrarios del país, es el que tienen mayor importancia para la población de esta región.

Tres de los cuatro ejidos constituyeron empresas sociales para el uso y aprovechamiento de sus recursos. De hecho, ese fue uno de los criterios que emplearon las juntas intermunicipales para seleccionar a los ejidos que participarían en este proyecto: que contaran con una empresa social, o en su defecto, que estuvieran considerados dentro de la tipología de la Comisión Nacional Forestal como productores con capacidad de transformación y comercialización, de tipo IV, porque disponen de infraestructura para transformar bienes y servicios en productos y subproductos terminados para su comercialización directa en los mercados.

- Servicios Ecoturísticos de El Jorullo. Empresa exitosa del sector social, con figura asociativa de cooperativa, es considerada modelo de empresa social; cuenta con servicios diversos como tirolesa, negocio de alimentos y bebidas, paseos en mulas y motocicletas, estudio de fotografía y video, una tequilera y una tienda. Además diariamente promueven eventos para sus clientes, de tipo artístico y cultural.

- Productos de Servicios Ambientales San Miguel, Sociedad de Producción Rural de Responsabilidad Limitada. Realiza diversas actividades productivas como el aprovechamiento forestal maderable principalmente, aprovechamiento de lechuguilla y envasado de agua de manantial, así como en actividades de turismo de naturaleza.

- En el caso del ejido Barranca del Calabozo, el aspecto productivo maderable, es la actividad que de manera colectiva define al ejido por su tradición de varios lustros en la actividad de aprovechamiento y transformación de materias primas.

- Santiago de los Pinos. Las actividades económicas están enfocadas principalmente a agricultura, ganadería, la minería y lo forestal.

Análisis de la integración de las empresas sociales

La composición de las empresas sociales de estos ejidos es determinante para conocer los consensos que se han dado al interior, pero también son indicadores de las posibilidades futuras de desarrollo y de la aparición de potenciales conflictos entre ejidatarios.

En el caso del ejido Barranca del Calabozo son socios de la empresa social todos los ejidatarios, casos diferentes son los de El Empedrado y el Jorullo, que no incluyen a todos los ejidatarios como socios, incluso tienen registrados a personas que no son ejidatarios como socios.

La intervención de no-ejidatarios en la empresa social podría derivar en conflictos y desacuerdos internos en el mediano plazo en el ejido El Empedrado, ya que la empresa es de reciente creación y aún no se pude advertir la potencial conflictividad de esta situación. Pero en El Jorullo y Anexos, hay una aceptación tácita de que haya socios no-ejidatarios pues en el momento de creación de la empresa social, fue decisión personal de cada ejidatario de si ingresaban o no. En asamblea general se tomó el acuerdo y al parecer es sólido, pues no se observa que ponga en riesgo la estabilidad del ejido. De cualquier forma, está abierta la posibilidad de ingresar como socio activo pero con la participación del pago de las acciones económicas correspondientes; destacando que el ejido como institución comunitaria tiene una acción dentro de la empresa y sus respectivos beneficios y reparto de utilidades.

Las experiencias de estas empresas muestran que debe haber un amplio consenso en las asambleas respecto de la integración de las empresas sociales, pues su funcionamiento puede obstaculizarse con el surgimiento de conflictos expresos o latentes, cuando las decisiones, sobre todo aquellas trascendentales como la incorporación de no-ejidatarios, no son tomadas con un convencimiento pleno.

Para el caso del ejido El Empedrado, existe la posibilidad de que en un futuro próximo se generen conflictos que detengan el proceso productivo por la discrepancia y reclamo por la exclusión de ejidatarios con derecho a ser socios de la empresa. Esto puede emerger en el momento del reparto de utilidades y la rendición de cuentas o al cambio de los representantes, ya sea agrarios o de la empresa. Sí el conflicto emana, será por el reclamo de las ganancias de la materia prima antes

del proceso de transformación, o sea de las ganancias del aprovechamiento y no así de la ganancia de la madera aserrada, pero podrá escalar, ya que es la empresa quien comercializa dicha materia prima. Como esta materia prima es madera de todos los ejidatarios y no todos han sido considerados en la empresa, iniciaran un reclamo focalizado por el dinero circulante que genera; esencialmente, las ganancias de la materia prima vendida a la empresa de aserrío, y posteriormente por la inclusión de esos ejidatarios en la empresa.

Estructura social

La estructura social que presentan los núcleos agrarios es la que se plantea en la legislación agraria: la asamblea general de ejidatarios como máximo órgano de decisión, las autoridades del comisariado ejidal como representantes legales de su núcleo agrario y que ejecutan los acuerdos de la asamblea, y algunos comités locales.

Cabe mencionar que hay claridad en cuanto a los ámbitos de intervención de cada autoridad, ya que los comisariados ejidales y los gerentes o directores de las empresas rinden cuentas a la asamblea general de ejidatarios, pero su rol es diferente.

Capital social y gobernanza local

Al hacer el análisis de los ejidos participantes, se revisaron varios puntos como el nivel de la colaboración social entre los diferentes grupos en el interior del ejido y el uso individual de las oportunidades surgidas a partir de ello, teniendo tres fuentes principales: la confianza mutua, las normas efectivas y las redes de amistad, parentesco y grupos de trabajo (tejido social).

De manera general, se observó que hay algunas debilidades en estos aspectos, ya que no hay trabajos colectivos de beneficio común, sino que sólo se realizan si hay un pago de por medio. Sin embargo, en otros ámbitos sí se encontraron lazos de confianza como en la organización de las fiestas de los diferentes poblados, en donde es posible encontrar disposición para colaborar y cooperar económicamente para la organización y realización de estos eventos. Hay también algunos casos aislados donde se puede percibir el apoyo mutuo, como es el caso del ejido Santiago de Pinos, cuya experiencia de siembra colectiva es un gran aliciente para impulsar procesos de organización en el ejido.

Dentro de los ejidos se evidencian ciertos liderazgos naturales, que sí bien han impulsados a los ejidos a iniciar procesos productivos como la transformación de materias primas y el ecoturismo, también pueden ser cotos de poder que, al concentrar mucho peso en las decisiones, puedan generar en un futuro viciar el proceso o desvirtuarlo. Las asambleas, así como las estructuras jerárquicas y los órganos de representación se ven opacadas en algunos casos como en El Empedrado y Santiago de los Pinos por los liderazgos, mismos que asumen este rol mutuamente de lucha de poderes.

El ejido que no tiene una asociación formal para la comercialización de sus materias primas es el Ejido Santiago de los Pinos. El ejido ha vendido madera y cuenta con un programa de manejo forestal maderable autorizado por Semarnat, el cual está actualmente suspendido por la afectación de muérdago que le ha dañado aproximadamente 700 has. diagnosticadas hasta hace dos años y con tratamientos para sanear al bosque que no han tenido éxito, al igual que en el ejido El Empedrado que ha sido atacado con la misma especie de muérdago.

Las asambleas agrarias de estos dos ejidos han tomado cartas en el asunto con sus ingenieros forestales que los asesoran pero ciertas condiciones legales sobre los saneamientos les han impedido tener resultados favorables y la plaga sigue avanzando y reproduciéndose.

En todos los ejidos participantes, la asamblea, en algunos aspectos como en el acceso a los recursos comunes, tiende a tomar decisiones generales; en las decisiones particulares y donde la mayor cantidad de responsabilidad debería recaer en los integrantes del comisariado, frecuentemente se sustenta en los liderazgos y en las empresas sociales, las cuales están actualmente representada por los liderazgos de manera formal.

Asambleísmo y toma de decisiones

En los cuatro ejidos, las asambleas son programadas con anticipación cada 30 o 60 días. El poder de convocatoria no es en sí una fortaleza del núcleo agrario pero no dejan de asistir a las reuniones, que se llevan a cabo con un número de ejidatarios que en algunos casos apenas alcanzan el quórum legal. La asamblea toma decisiones sobre temas comunes, principalmente aspectos de territorio en términos de la tenencia, y en el aspecto forestal últimamente. Los grupos dentro de la asamblea están poco polarizados en la mayoría, a excepción de El

Empedrado donde los liderazgos naturales tienen con su opinión un gran peso en la toma de decisiones, los comisariados siendo representantes legales están confrontados con los liderazgos naturales. Esta situación es compleja y deben trabajarse puntos de coincidencia e interés que den esfuerzos en concurrencia y no en contra.

Seguimiento a procesos

Ha sido basado en las estructuras locales existentes, y se ha impulsado por los liderazgos, y algunos procesos en el comisariado, principalmente en los generados con solicitudes en las que el representante legal tiene que impulsar y autorizar como con solicitudes de la Comisión Nacional Forestal y otras dependencias como Semarnat para la autorización de los saneamientos, esto para el caso de Santiago de Pinos y El Empedrado.

En el caso del manejo forestal, aún continúan sustentados en sus Prestadores de Servicios Técnicos Forestales (PSTF) para decidir las acciones, sin que en esta etapa de manejo se hayan apropiado de ella por parte del ejido; aún la información y decisión se basa solamente en los PSTF y el conocimiento locales de los ejidatarios no se toma en cuenta (p.ej. el conocimiento sobre la observación del ciclo de la plaga presente o el rumbo de crecimiento de esta misma que para los ejidatarios es muy clara)[2].

Los comités locales funcionan de acuerdo a la necesidad humana a que responden, por lo general en los ejidos la aportación individual de trabajo para beneficio colectivo es más de manera efímera. Lo momentáneo es más común que lo permanente dentro de las actividades. La premura de terminar los procesos hacen que se cumplan las funciones y acciones, pero por presión o insistencia externa, más que por ejecutar un plan de corto, mediano o ya no decir de largo plazo. Los comités de agua potable, de prevención de incendios forestales, de salud, de padres de familia, son comités que funcionan permanentemente, consultan y se adecuan. Los comités que han logrado permanecer dentro de las comunidades a través del tiempo son precisamente los que han nacido y partido de necesidades concretas colectivas.

---

[2] Taller de auto-diagnóstico socio-ambiental en el Ejido Santiago de Pinos, llevado a cabo el día 1 de agosto de 2013 donde se analizó el problema de la plaga por muérdago y describieron las fases y ciclos de vida de la especie. Así también hicieron una propuesta comunitaria sobre la atención fitosanitaria. Incidencia Social A.C. / CONAFOR.

Roles y equidad

Dentro del rol o sistema de cargos, los ejidos presentan una condición de poca participación de la mujer y de grupos vulnerables, que frecuentemente son susceptibles a embates de diversas condiciones de marginación. Los roles y la forma de ocuparlos, dentro del ejido, son poco institucionalizados y dependen no de un sistema formal de cargos por escalafón o de democratización de la elección del candidato a ocupar un cargo, sino están más acostumbrados a impulsar, y en algunos casos, a imponer algún cargo unilateralmente con criterios de parentesco, afinidad y cercanía por grupo de interés común, con altos niveles de discrecionalidad.

Grupos de poder excluyentes e incluyentes

Los grupos incluyentes dentro de los ejidos están vinculados con las estructuras de poder de liderazgos y de las empresas sociales, principalmente por la generación de empleo y movimiento y flujo de efectivo circulante. Esta condición de poder, paradójicamente excluye también a otros grupos, como a las personas no afines a los liderazgos y confronta y desestima las estructuras e instituciones formales comunitarias. En este periodo en dos de los ejidos no se han generado muchas sinergias ni acompañamiento mutuo entre grupos de poder como el comisariado y la empresa o el comisariado y el liderazgo, caso de El Empedrado y Santiago de Pinos, respectivamente. En este sentido, los otros dos ejidos si han logrado hacer sinergias y acuerdos donde se han realizado una valoración colectiva de los beneficios de la coparticipación. Otro grupo de poder es el derecho y acceso a la tierra, tanto de manera formal como semi-informal. Esta condición también genera dentro del ejido exclusiones a personas no afines al comisariado y viceversa, inclusiones en algunos casos particulares, como a personas allegadas o afines al grupo de poder en turno.

Arreglos, acuerdos y normas

Aunque los cuatro ejidos cuentan con su reglamento interno, hay debilidades en su aplicación. Es un documento que no ha cumplido con su cometido de regular diversos ámbitos de la vida ejidal, pues sólo se aplica para resolver aspectos muy generales y no se ha fortalecido después de la elaboración del Ordenamiento Territorial Comunitario que algunos ejidos han realizado. De hecho, han sido más funcionales los acuerdos tomados en las asambleas, pues han tenido un mayor seguimiento por parte de las asambleas.

Los acuerdos de largo plazo son fuertes en la medida de que sean estrictamente necesarios como es el tema de la empresa, el respeto al acceso a la tierra, las rendiciones de cuentas y los aspectos de convivencia que están en el ámbito municipal, dentro de estos acuerdos que permanecen está la participación de un gran número de ejidatarios en el manejo forestal y las tomas de agua para consumo doméstico.

Respecto a los arreglos, se identificaron los relativos a la empresa y el manejo forestal (correlación de fuerzas), los cuales están en un andamio débil, que con aspectos mínimos detonan confrontaciones internas en el caso específico del ejido El Empedrado. Sobre sus fortalezas, los ejidos han logrado arreglos sobre el tema de ganado y agricultura.

Acceso a los recursos comunes

En lo que respecta al aspecto forestal y de tenencia y acceso a la tierra, el comisariado ejidal con el respaldo de toda la asamblea es el que busca la regulación basado en los acuerdos y normas, principalmente externas regulatorias como las leyes referentes al aprovechamiento forestal y la Ley Agraria. El acceso a recursos como el beneficio de arbolado para uso doméstico, leña, construcción u otro fin que no sea comercial, está permitido y se sustenta en acuerdos de asamblea siempre y cuando así lo soliciten. Las áreas destinadas para tal fin están marcadas en los OTC.

En el caso de las empresas ejidales, ha incursionado y ganado terreno al comisariado ejidal en el manejo reciente del bosque y el servicio ecoturístico. La plaga forestal, para Santiago de los Pinos y El Empedrado ha sido un aspecto en el que el comité de aprovechamiento y la empresa ejidal, respectivamente, han tomado iniciativa para el saneamiento y el comisariado ha asumido un papel más de acompañante del trámite necesario que promotor del mismo para realizar el saneamiento.

Presión sobre los recursos comunes

Las zonas bajas se han destinado para ser parcelas de labor desde hace muchos años en la mayoría de los ejidos; son tierras que presentan de baja a mediana presión, pues son trabajadas con rotación de cultivo y descansos anuales. Empero los grados de deforestación a partir del

aprovechamiento maderable seguramente han disminuido no se ha cuantificado estas tasas anuales.

La ganadería ha generado alta presión sobre las áreas, más aún las zonas forestales permanecen cuidadas y sin invasión del ganado; el OTC donde se ha instrumentado, ha contribuido a mejorar este aspecto de cuidado sobre el bosque.

Las actividades de monitoreo forestal en los ejidos, con las primeras iniciativas para realizarlo se basaron en la preocupación legítima del saneamiento forestal para el caso de dos ejidos, que es evidente como una preocupación generalizada. Las dependencias y actores como los técnicos forestales que tienen la responsabilidad solidaria del ejido en términos del manejo forestal ante SEMARNAT, han contribuido a la propagación de la plaga de muérdago. Esta situación deja en franca zozobra operativa a los integrantes del ejido pues no cuentan con elementos técnico-legales para enfrentar el problema, porque la normatividad forestal mexicana carece de esquemas de manejo, saneamiento y sustento legal, a criterios del saneamiento necesarios para combatir estas plagas presentes en los ejidos; con ello, los intentos realizados les ha implicado grandes esfuerzos, sin resultado favorable; esto ha sido causante exponencial de la propagación del muérdago.

La participación de las juntas intermunicipales, ha ganado un nivel de confianza y en el mediano plazo podrán ser eslabón importante para que el ejido a través del monitoreo y con el acompañamiento de estas juntas puedan plantear a SEMARNAT, PROFEPA Y CONAFOR, un nuevo esquema para atacar la plaga forestal, basado en el monitoreo comunitario, el conocimiento en la observación y la experiencia, los Técnicos Forestales, para que en conjunto con las Juntas, presenten una alianza para la gestión del manejo integrado del territorio y de ser una institución promotora de nuevos esquemas de manejo integrado de la cuenca.

Este trabajo busca contribuir a la reflexión de la forma y del fondo para trabajar con los ejidos y comunidades donde se tenga una iniciativa local de establecer comités de monitoreo de recursos naturales; al mismo tiempo, para que se planteen algunos elementos y aspectos que debe de considerarse y conocerse previamente como sus costumbres, acuerdos, normas, arreglos, tradiciones, estructuras así como durante el proceso de aprendizaje mutuo donde se pretende partir de la base y el reconocimiento de los conocimientos comunitarios basados en la experiencia y la tradición.

ACTIVIDADES REALIZADAS

De acuerdo con el propósito de facilitar a los ejidos del proyecto piloto que se encuentran en las Cuencas Costeras de Jalisco su preparación para instalar un sistema de monitoreo comunitario, se realizaron actividades con un enfoque participativo y acordes con el perfil de los ejidatarios y especialmente de quienes integrarían los comités de monitoreo comunitario; se emplearon técnicas basadas en la educación popular, que es una propuesta metodológica apropiada para el trabajo de facilitación de procesos de desarrollo comunitario, con el que es posible desarrollar alternativas de superación, construyendo colectivamente oportunidades para avanzar a partir de las realidades que viven los ejidatarios.

Uno de los principios fundamentales de la educación popular es que se basa en dinámicas y aspectos sociales complejos locales, para promover el desarrollo de ejidatarios y comuneros y propiciando que ellos se conviertan en los actores del cambio por medio de la identificación de sus problemas, la investigación de sus manifestaciones, el análisis de sus causas y consecuencias, la formulación de alternativas y organizándose para llevarlas a cabo.

Se aplicaron herramientas participativas de diagnóstico como la identificación de los *actores internos y externos* del ejido, el *diagrama de Venn*, *dinámicas de conocimiento* como la del "*objetivo*", *mapas regionales*, *calendarios estacionales*, *saberes locales*, *maquetas de cuencas*, entre otras.

Los facilitadores y acompañantes del proceso del programa piloto monitoreo comunitario en los cuatro ejidos propuestos, desarrollaron las siguientes actividades:

> *Visitas de presentación y acercamiento con las autoridades locales, así como un recorrido de campo*. Durante las primeras visitas se conocieron las estructuras y esquemas de gobernanza local de los núcleos agrarios (autoridades auxiliares, sistemas de cargos, usos, costumbres y tradiciones), así como sus principales actividades económicas y de desarrollo comunitario. El recorrido se planteó con la intención de entender las problemáticas y preocupaciones de los ejidatarios con respecto al manejo y conservación de sus recursos y su territorio.
>
> *Acopio de información e investigación documental*. También fue necesaria la búsqueda de información en fuentes documentales y

electrónicas para contar con antecedentes y contextualizar el trabajo con los ejidos. Es un punto en el que se debe poner atención, ya que la información es fundamental para dar un contexto a cualquier iniciativa de desarrollo en los ejidos; sin embargo, en este caso particular se observó que los núcleos agrarios no cuentan con estos datos en sus archivos, ni tampoco las instituciones gubernamentales que han operado algún proyecto o estudio en dichos ejidos, tiene a disposición los resultados. Fue necesario obtener una parte de los documentos por medio de solicitudes de información basados en la Ley Federal de Transparencia y Acceso a la Información pública Gubernamental, que es un procedimiento tardado.

Aplicación de entrevistas semi-estructuradas con actores claves. Se entablaron diálogos con algunos actores el proceso, destacadamente con las autoridades de los núcleos agrarios y funcionarios de entidades gubernamentales locales y del estado.

Reuniones con actores externos a los ejidos –especialmente con las juntas intermunicipales-, para identificar su aportación a los comités de monitoreo comunitario y el reconocimiento del papel que juegan como instituciones intermunicipales y los alcances de la interacción de su personal con los ejidos.

Asambleas ejidales para la definición de los problemas específicos de cada núcleo agrario y estrategias de acción para solucionarlos. Se abordaron los temas de gobernanza y revalorización de los recursos y el territorio, asimismo se obtuvieron los acuerdos de integrar un comité de monitoreo comunitario, su función y resultados. En esta asamblea se especificaron los temas que son prioritarios de atender e importantes de monitorear a partir del conocimiento local aunado a la construcción y adquisición de métodos de mediciones más sistemáticas. Los temas más relevantes fueron plagas forestales (muérdago), medición y manejo forestal (incremento de volúmenes) y agua.

Desarrollo de capacidades. Los temas fueron dados con base en el análisis de los comités integrados en cada asamblea; dentro de este mismo rubro, se realizaron reuniones de intercambio de experiencias para socializar el proceso en el que se abordaría el proyecto. Para apoyar esta actividad, se consiguió apoyo del Servicio Forestal de Estados Unidos para que donara equipo y materiales para la medición forestal, el cual está a resguardo de cada junta intermunicipal.

Desarrollo de capacidades

Los enlaces operativos de las juntas intermunicipales fueron piezas clave en la planeación y desarrollo de las etapas siguientes. Porque propiciaron el acercamiento a los ejidos y acompañaron el proceso comprometiéndose en ocasiones algunas de las circunstancias que se presentaban con base a las necesidades de los comités.

La capacitación se realizó en dos vertientes. La primera, fue orientada a obtener un diagnóstico participativo del núcleo agrario. Indudablemente, el uso de dinámicas y de técnicas de corte participativo en cualquier actividad de desarrollo de capacidades es indispensable, especialmente para la elaboración de diagnósticos comunitarios, porque permiten tener información de primera mano para la identificación de las necesidades locales, pero también para ubicar áreas de oportunidad para detonar, fortalecer o acompañar procesos de desarrollo comunitario. Con este tipo de herramientas podemos identificar las circunstancias en las que se encuentra un núcleo agrario y sobre todo su apropiación de cada circunstancia, porque son los actores locales quienes las viven cotidianamente.

Hay casos en que los ejidos ya cuentan con diagnósticos o proyectos en ejecución, sobre todo aquellos que han iniciado un proceso de desarrollo forestal comunitario, y éstos pueden ser de gran utilidad para conocer la vida en el interior de la comunidad, los documentos que se requieren son: evaluación rural participativa, estudios de ordenamiento territorial comunitario, informes de cursos de capacitación, proyectos de inversión o productivos, entre otros.

Aunque la creación de un comité de monitoreo comunitario de recursos naturales requerirá en mayor medida información documental que de tallereo para elaborar su diagnóstico, es importante dedicarle un par de talleres para afinar e identificar las fortalezas y las amenazas internas y externas, así como para tener localizados los actores locales que intervienen en la vida del ejido o comunidad agraria, porque éstos son dinámicos y presentan un proceso de cambio continuo.

La segunda vertiente del desarrollo de capacidades enfatiza que partiendo de la identificación de los conocimientos y capacidades locales, se requirió adaptar los talleres y reuniones a cada una de las temáticas que corresponden a las necesidades y problemáticas a resolver internamente. Las temáticas específicas fueron las siguientes:

- Dos ejidos presentaron como prioridad para el monitoreo la plaga de muérdago que afecta gravemente a especies de Pinus spp, en

este caso la capacitación versó sobre técnicas que permitieran cuantificar superficie mediante el SIG y daños mediante criterios forestales.

- Un ejido decidió monitorear su aprovechamiento forestal, y para ellos se capacitó en metodologías de medición forestal básica de volúmenes y apegados a su PMFM.
- Un ejido eligió el tema de monitoreo de agua, los aprendizajes fueron sobre medición de caudales y cantidades del recurso hídrico. El tema de calidad de agua no se abordó por la carencia de equipo básico necesario para dicho monitoreo, dado que la inversión para la compra de los kits adecuados a la metodología de Global Water Watch es grande. De cualquier forma, no se percibió que la medición de la calidad sea eje motor del comité (aunque no la descartan como importante).

Primer ejercicio de monitoreo. Luego de la capacitación técnica básica, se definieron y acordaron conjuntamente las áreas, sitios, amplitudes, intensidades de muestreo y delimitación de áreas. De este primer ejercicio de monitoreo se generaron diversas dudas, sobre todo con respecto al uso y manejo de instrumentos de medición y también sobre la metodología a utilizar para los sitios de monitoreo y las intensidades de los muestreos. En esta etapa se realizó una recapitulación del proceso en diferentes ámbitos:

- Social: evaluación del nivel de aceptación del proyecto, conceptualización y participación, utilidad del monitoreo para la toma de decisiones, fortalecimiento de la institución comunitaria.
- Ambiental: identificación de actividades de protección, conservación y manejo identificadas con oportunidad de fortalecerlas. Grado de apropiación.
- Económica: identificación de la relación costo-beneficio del monitoreo. Inversión de largo plazo y beneficios secundarios.

En cada núcleo agrario se analizaron aspectos del proceso de manera cronológica, específicamente de los sucesos ocurridos. También se emprendió el tema de la planificación de cómo abordar y entregar la información a la asamblea y una propuesta de compromiso del ejido para el seguimiento del proceso.

Resultado del análisis fueron las iniciativas internas generadas por la construcción colectiva del modelo de atención a la problemática ambiental concreta a monitorear. Esta propuesta de modelo de atención, se llevaría a la asamblea para afianzar compromisos y su complejidad dependió de varios factores internos como las capacidades

técnicas previas existentes de los ejidatarios, los conocimientos y aptitudes, compromiso social de cada integrante del comité, conocimiento general del predio, arraigo y sentido de pertenencia.

Las asambleas de cada núcleo agrario, recibieron la información por parte del Comité Interno sobre el tema en específico monitoreado y destacaron la importancia de continuar con el monitoreo y tomar acciones y decisiones con los resultados del mismo. En esta asamblea se trabajó explícitamente la importancia de la información generada, pero implícitamente la fortaleza de que esta información se generó a partir de los mismos ejidatarios, rompiendo así un dogma común en las comunidades ("requiere venir alguien externo para que la información sea verdad, sea lo que sea, es más creíble si proviene de un externo"). A esto se le llama validación social y auto-reconocimiento comunitario.

Con este capital humano instalado, con el reconocimiento de la asamblea de las nuevas capacidades locales y con la información generada por el mismo comité, se tomó la decisión de atender la problemática inicial. En tres de los cuatro ejidos hubo respuestas significativas que se tradujeron en acciones como esquemas de atención de la plaga, un modelo de restauración de suelos y agua, así como el fortalecimiento del sistema de manejo comunitario de su bosque, respectivamente.

La sistematización se llevó a cabo durante todo el proceso estando estructurado con una base crono-temática, de acuerdo a las actividades, que estuvieron apegadas a una planeación con las propias comunidades sobre los tiempos y etapas del proceso que se diseñaron.

LA EXPERIENCIA

La integración y fortalecimiento de comités de vigilancia de los recursos naturales en cuatro ejidos del estado de Jalisco fue una experiencia enriquecedora desde diferentes puntos de vista, pero sin duda enfocarla como un proceso de enseñanza-aprendizaje, con una fuerte orientación participativa, deja lecciones que pueden replicables en ejidos o comunidades agrarias similares en circunstancias semejantes.

En los ejidos y comunidades agrarias del país, cualquier iniciativa de desarrollo en la comunidad -social, económico, productivo- ineludiblemente debe considerar el componente de formación y capacitación como un ingrediente necesario para generar habilidades, destrezas y capacidades que permitan a los pobladores de los núcleos agrarios tomar en sus manos las riendas de su propio desarrollo.

Este tipo de procesos no son lineales ni responden a recetas inflexibles, en el proceso se presentaron diversos retos dados precisamente por el nivel organizativo de los ejidos, sus usos y costumbres, el capital social, la manera en que se apropiaron de sus empresas sociales y la sensibilidad y concientización hacia sus recursos naturales. También, por indicadores más específicos como el perfil de sus habitantes: personas adultas, con cierto grado de analfabetismo funcional, y con dificultades para el aprendizaje conceptual.

En el siguiente apartado se sistematizan los momentos etapas, procesos y actores significativos de la integración de comités de monitoreo. Son un ensayo que busca generar información útil para la creación de más grupos de monitoreo en otros núcleos aprovechando la experiencia que tuvieron los cuatro ejidos del estado de Jalisco.

Actividad	Actores	Logros	Aprendizajes
V*isita inicial de presentación y acercamiento,* así como un *recorrido de campo*.	EJIDOS<br>CONAFOR<br>LAIF<br>JUNTAS INTER-MUNICIPALES<br>PEACE CORPS U.S.<br>INCIDENCIA SOCIAL A.C.	Permitió el acercamiento con los cuatro ejidos seleccionados, se reconocieron las estructuras y esquemas de gobernanza local de cada uno de los ejidos, además de conocerse tanto los sistemas de cargos existentes en cada ejido como sus usos, costumbres y tradiciones, así como sus principales actividades económicas y de desarrollo comunitario.<br>El recorrido en específico, llevó a la explicitación de las problemáticas y preocupaciones de los ejidos con respecto al manejo y conservación de sus recursos y su territorio.	Aunque en los cuatro ejidos se reconocieron estructuras sociales dadas por las figuras que se establecen en la Ley Agraria (asamblea general, comisariado ejidal, consejo de vigilancia), el funcionamiento de éstas como mecanismos para el análisis y resolución de sus problemas –y no sólo como medios instrumentales- fue muy diferenciado.<br>El potencial organizativo de un núcleo agrario es muy probable que se vea reflejado cuando hay asambleas concurridas y con participación activa de los ejidatarios, cuando las autoridades que lo representan tienen las competencias necesarias para coordinar y promover la participación y los esfuerzos colectivos, y cuando existe un respeto por las normas, escritas o no, que ellos mismos se han dado para la convivencia y funcionamiento del ejido.<br>Es una condición tener un buen nivel organizativo para constituir y poner a funcionar un comité de monitoreo comunitario, ya que se trata de una trabaja especializado que requiere el consenso y la participación de la asamblea de ejidatarios, el apoyo de las autoridades y el desarrollo de capacidades para funcionar. El comité de monitoreo comunitario de hecho significa la apropiación de un proceso técnica y organizativamente complejo que antes era manejado exclusivamente por asesores técnicos.

Actividad	Actores	Logros	Aprendizajes
Búsqueda y análisis de información documental	INCIDENCIA SOCIAL A.C.<br>LAIF<br>CONAFOR	Permitió conocer aún más las condiciones sociales, económicas, ambientales y culturales de los ejidos	El acercamiento de información de los ejidos es fundamental para diagnosticar el estado que guardan. En este sentido, se observó una gran debilidad tanto de los propios núcleos agrarios como de las instituciones que los apoyan, ya que no existe una cultura de conservar y ordenar los documentos y archivos con la información básica de éstos. En los cambios de autoridades locales, es muy frecuente que desaparezcan documentos, por lo que es necesario sensibilizar a las asambleas sobre este punto, a efecto de que haya pequeños centros de documentación en los ejidos.<br>Las dependencias públicas también tienen mucho qué hacer en el ordenamiento de sus documentos.<br>Una forma de gestionar información –que además detona el ejercicio de un derecho ciudadano- es el uso de la información pública por medio de solicitudes que se presentan ante las dependencias públicas. Los procedimientos para hacerlo se encuentran en las leyes de transparencia y acceso a la información federal y estatales.

Actividad	Actores	Logros	Aprendizajes
Talleres para la generación y análisis de información	INCIDENCIA SOCIAL A.C.<br>LAIF<br>CONAFOR<br>JUNTAS INTER-MUNICIPALES	Durante el acompañamiento y seguimiento al proceso de cada uno de los comités de monitoreo comunitario, se realizaron entrevistas semi-estructuradas con actores clave y la aplicación de herramientas participativas de diagnóstico como la identificación de los actores internos y externos del ejido, el diagrama de Venn, dinámicas de conocimiento como la del "objetivo", mapas regionales, calendarios estacionales, saberes locales, maquetas de cuencas…	Durante el proceso de integración y fortalecimiento de comités de monitoreo de los recursos naturales se emplearon herramientas e instrumentos participativos que tuvieron una doble función. En principio, fueron útiles para recabar información en voz de los principales actores del proceso, los ejidatarios, con la cual se elaboraron diagnósticos y contribuyó también al análisis de temas y problemas específicos. Pero también cumplieron una función pedagógica, en virtud de que permitieron la reflexión y análisis en reuniones y talleres tanto de sus problemas como de posibles soluciones.<br>Este tipo de procesos en los que se busca el desarrollo de capacidades de los ejidatarios requieren como condición ser de carácter participativo, si lo que se busca es generar impactos más cercanos a los objetivos propuestos; cualquier otra estrategia tendrá resultados pobres.

Actividad	Actores	Logros	Aprendizajes
Talleres para la generación y análisis de información	INCIDENCIA SOCIAL A.C.<br>LAIF<br>CONAFOR<br>JUNTAS INTER-MUNICIPALES	Se efectuaron reuniones y acercamientos con personal de la JISOC y JIRCO para identificar el involucramiento y grado de participación de ellos hacía los comités de monitoreo comunitario y el reconocimiento del papel que juegan como institución intermunicipal y la interacción de su personal con los ejidos	Hubo un intercambio de opiniones respecto del monitoreo. Se buscó que las juntas fueran las encargadas de la gestión y acompañamiento del monitoreo. No se tuvo claro los alcances de la participación de las juntas, eso obstaculizó y retrasó algunas de las acciones. Se observó un involucramiento diferenciado de parte de las dos juntas, y esto se debió a que hay personal que ha logrado tener presencia entre las comunidades rurales de la región. Las juntas coadyuvaron a acercar a los diferentes actores con los representantes de los núcleos agrarios. Claves para procesos exitosos -que se defina claramente el papel de las juntas -que las juntas funjan como vínculo entre los asesores técnicos de cada región. -que lleven un registro y seguimiento de los proyectos de uso y aprovechamiento de recursos naturales de los ejidos

Actividad	Actores	Logros	Aprendizajes
Asamblea inicial y creación del comité comunitario de monitoreo	EJIDOS<br>CONAFOR<br>LAIF<br>JUNTAS INTER-MUNICIPALES<br>PEACE CORPS U.S.<br>INCIDENCIA SOCIAL A.C.	Las asambleas ejidales se llevaron a cabo y se dialogó sobre la preocupación de los núcleos agrarios en torno a las problemáticas concretas de cada núcleo agrario y su interés de poder tomar acciones sobre un tema en específico.<br>Se abordaron los temas de gobernanza y revalorización de los recursos y el territorio así como los acuerdos de integrar un comité de monitoreo comunitario, su función y resultados. En esta asamblea se especificaron los temas que son prioritarios atender e importantes de monitorear a partir del conocimiento local aunado a la construcción y adquisición de métodos de medición más sistemáticos.<br>Los temas más relevantes fueron plagas forestales (muérdago), medición y manejo forestal (incremento de volúmenes) y agua.	Fue diferenciado el involucramiento de las asambleas en el proceso de monitoreo. En aquellos ejidos donde las asambleas se involucraron y participaron de manera activa, se obtuvieron resultados positivos en el análisis y definición del recurso a monitorear, la organización para la integración del comité de monitoreo y en la puesta en marcha de esta actividad.<br>En los núcleos agrarios donde no hubo asambleísmo fuerte, el resultado de los trabajos fue deficiente. En uno de los ejidos, el proyecto se vino abajo porque se observó un problema de rendición de cuentas de parte de las autoridades hacia los ejidatarios, y por lo tanto no convocaban a asambleas.<br>Las asambleas de ejidatarios tienen un rol de primera importancia en este proceso de construcción de comités de monitoreo, pues si no dan todo su apoyo a los integrantes de dichos comités, es muy probable que nazcan con una debilidad congénita que solo llevará a erogar recursos financieros y esfuerzos.

Actividad	Actores	Logros	Aprendizajes
Construcción de conocimientos	EJIDOS CONAFOR LAIF JUNTAS INTER-MUNICIPALES PEACE CORPS U.S. INCIDENCIA SOCIAL A.C. CORPS U.S. SIERRA GORDA INCIDENCIA SOCIAL A.C.	Capacitación: Con los temas ya definidos por cada núcleo agrario a monitorear y analizando la pertinencia en ese momento del proyecto, se realizaron capacitaciones concretas que se programaron de acuerdo a cada necesidad local. Los comités integrados en cada asamblea se citaron a una reunión de intercambio de experiencias entre ellos para generalizar el proceso en el que se abordaría el proyecto, con acuerdo de tiempos y compromisos de las partes.<br>Los dos ejidos que priorizaron el monitoreo de la plaga de muérdago que afecta a especies de *Pinus spp,* la metodología fue la apegada a cuantificar superficie mediante el SIG y daños mediante criterios forestales.<br>Para el ejido que eligió monitorear su aprovechamiento forestal, se ocuparon las metodologías de medición forestal básica de volúmenes y apegados a su PMFM.<br>Para el caso del ejido que eligió el tema de monitoreo de agua, se aplicó solo la medición de caudales y cantidades. El tema de calidad de agua quedó suspendido por la ausencia de equipo básico necesario para dicho monitoreo, dado que la inversión es fuerte en la compra de kits adecuados a la metodología de Global Water Watch, que momentáneamente no se percibe que la medición de la calidad sea eje motor del comité (aunque no la descartan como	Para que los comités de monitoreo comunitaria tuviera herramientas para comenzar su trabajo, se requirió impartir capacitación en temas específicos como plagas forestales, medición y manejo forestal (incremento de volúmenes) y agua.<br>Antes, la asamblea general de ejidatarios realizó el análisis y la determinación de objeto de monitoreo.<br>Enseguida, se diagnosticaron las necesidades de capacitación de acuerdo al recurso que se monitoreó, y se gestionaron recursos para cubrir el costo de la capacitación.<br>Estos dos puntos son complejos porque se requieren conocimientos técnicos básicos para la definición del problema que será monitoreado, el despacho o institución más apropiado para impartir la capacitación, y la capacidad de gestión para obtener recursos que solventen los gastos.<br>Otros de los puntos que deberán tener mucha atención es la dificultad en la interpretación de datos, ya que se emplea un lenguaje especializado difícil de entender por ejidatarios.<br>Adicionalmente, como servicio especializado, los integrantes del comité de monitoreo requerirá de recursos

	importante).<br><br>Paralelamente se consiguió apoyo del Servicio Forestal de Estados Unidos quien donó equipo y materiales para la medición forestal, el cual está a resguardo de cada junta intermunicipal.	financieros para cubrir viáticos, gastos de papelería y honorarios.<br>Finalmente, hay que comentar que el equipo e instrumental para realizar algunos monitoreos es costoso, por lo que este punto se puede convertir en un cuello de botella.<br>Se puede concluir entonces, que en su fase de inició, la integración de comités de monitoreo comunitario requerirán el acompañamiento de asesores técnicos que den impulso a las iniciativas de los ejidos.

Actividad	Actores	Logros	Aprendizajes
Construcción de conocimientos	EJIDOS<br>CONAFOR<br>LAIF<br>JUNTAS INTER-MUNICIPALES<br>PEACE CORPS U.S.<br>INCIDENCIA SOCIAL A.C.<br>CORPS U.S.<br>SIERRA GORDA<br>INCIDENCIA SOCIAL A.C.	Se inició la capacitación partiendo de la identificación de los conocimientos y capacidades locales, específicamente con cada una de las temáticas que corresponden a las necesidades y problemáticas a resolver internamente	Es fundamental el análisis de los problemas que se quieran resolver, no sólo por las afectaciones en diversos ámbitos (social, económico, ambiental), sino también considerando los alcances y posibilidades reales del propio ejido, en términos de su capacidad organizativa, complejidad del problema, disponibilidad de recursos financieros, capacidad de gestión, entre otros.

Actividad	Actores	Logros	Aprendizajes
Primer monitoreo comunitario	EJIDOS<br>CONAFOR<br>LAIF<br>JUNTAS INTER-MUNICIPALES<br>PEACE CORPS U.S.<br>INCIDENCIA SOCIAL A.C.	Posterior a la capacitación técnica básica, se definieron y acordaron conjuntamente las áreas, sitios, amplitudes, intensidades de muestreo, delimitación de áreas y se realizó un Primer Ejercicio de Monitoreo.	Las primeras actividades son difíciles porque no hay experiencias previas en los ejidos sobre este tipo de trabajo. En este punto se requiere aún el acompañamiento de asesores técnicos y que quien guíe el proceso tenga suficientes capacidades para lograr integrar y motivar al comité

Actividad	Actores	Logros	Aprendizajes
Adecuación de aprendizaje y repaso de conocimientos	EJIDOS<br>CONAFOR<br>LAIF<br>JUNTAS INTER-MUNICIPALES<br>PEACE CORPS U.S.<br>INCIDENCIA SOCIAL A.C.	Del primer ejercicio de monitoreo surgieron dudas principalmente sobre el uso y manejo de instrumentos de medición y otras con respecto a la metodología a utilizar para los sitios de monitoreo y las intensidades de los muestreos.	

Actividad	Actores	Logros	Aprendizajes
Análisis y retrospectiva, adecuación y revalorización.	EJIDOS<br>CONAFOR<br>LAIF<br>JUNTAS INTER-MUNICIPALES<br>PEACE CORPS U.S.<br>INCIDENCIA SOCIAL A.C.	En esta etapa se realizó una recapitulación del proceso en los siguientes ámbitos<br>Social: evaluación del nivel de aceptación del proyecto, conceptualización y participación, utilidad del monitoreo para la toma de decisiones, fortalecimiento de la institución comunitaria.<br>Ambiental: identificación de actividades de protección, conservación y manejo identificadas con oportunidad de fortalecerlas. Grado de apropiación.<br>Económicas: identificación del costo beneficio del monitoreo. Inversión de largo plazo y beneficios secundarios<br><br>Para la recapitulación, en cada núcleo agrario, se analizó aspectos del proceso (cronológicamente), específicamente de los sucesos que hasta la fecha han ocurrido. La planificación de cómo abordar y entregar la información a la asamblea y una propuesta de compromiso del ejido para el seguimiento del proceso.	El producto de la recapitulación fueron las iniciativas internas generadas por la construcción colectiva del modelo de atención a la problemática ambiental concreta a monitorear.<br>Esta propuesta de modelo de atención, se llevó a la asamblea para afianzar compromisos y su complejidad dependió de varios factores internos como las capacidades técnicas previas existentes en los ejidatarios, los conocimientos y aptitudes, compromiso social de cada integrante del comité, conocimiento general del predio, arraigo y sentido de pertenencia.<br>Las asambleas de cada núcleo agrario, recibieron la información por parte del Comité Interno sobre el tema monitoreado y destacaron la importancia de continuar con el monitoreo y tomar decisiones con los resultados del mismo. En esta asamblea se trabajó explícitamente la importancia de la información generada, pero implícitamente la fortaleza de que esta información se generó a partir de los mismos ejidatarios. Esto se llama validación social y auto-reconocimiento comunitario.

Actividad	Actores	Logros	Aprendizajes
Aprobación y aceptación del modelo interno de atención a la problemática ambiental concreta	EJIDOS		Con este capital humano instalado, con el reconocimiento de la asamblea de las nuevas capacidades locales y con la información generada por el mismo comité, se tomaron decisiones de atender la problemática inicial. En tres de los cuatro ejidos la respuesta fueron acciones que detonaron procesos como esquemas de atención de la plaga, un modelo de restauración de suelos y agua, así como el fortalecimiento del sistema de manejo comunitario de su bosque, respectivamente.<br>La sistematización se llevó a cabo durante todo el proceso estando estructurado con una base crono-temática, de acuerdo a las actividades, que estuvieron apegadas a una planeación con las propias comunidades sobre los tiempos y etapas del proceso que se diseñaron.

LECCIONES APRENDIDAS

Indudablemente se tienen que respetar e identificar los esquemas de gobernanza local y sus estructuras sociales internas, además de la enmarcada en la legislación agraria como los órganos de representación y vigilancia del ejido o comunidad agraria (asamblea general, comisariado y consejo de vigilancia), también existen comités locales que tienen cierta representación y peso en la toma de decisiones en las asambleas.

Al inicio de cualquier proceso de desarrollo en los ejidos o comunidades agrarias se deben identificar, respetar, dialogar y llegar a acuerdos consensuados con los actores internos y externos que intervienen en la vida de la comunidad, como ejemplo podemos encontrar los siguientes:

Actores internos	Actores externos
Comisariado	Visitador agrario
Consejo de vigilancia	Asesores técnicos
Autoridad auxiliar municipal	Promotores institucionales
Consejo de principales	Instituciones gubernamentales
Comités comunitarios	Instituciones u organizaciones no gubernamentales
Liderazgos naturales	Juntas intermunicipales
Promotores comunitarios	Ayuntamientos
Entre otros	Entre otros

Los actores externos debemos de respetar la dinámica comunitaria (tiempos, usos, costumbres y tradiciones) así como las reglas escritas y no escritas a las que dan obediencia en la comunidad, con el afán de consolidar y fortalecer la confianza con ellos. En algunos ejidos tienen por costumbre reunirse determinado tiempo, ya sea a inicios o finales de cada mes o cada dos meses o tener estipulado un día en específico a los que nos debemos acoplar y sólo por causas de fuerza mayor realizar una convocaría, si es necesario, para tocar los asuntos que nos conciernen, exclusivamente.

Los actores externos que pretendan contribuir al desarrollo del ejido deben estar abiertos a otras posibilidades de facilitación, es decir, no enfocarse únicamente al asunto que nos atañe en ese momento, si está dentro de nuestra posibilidad también debemos contribuir u orientar la necesidad comunitaria.

De ser posible debemos entrelazar los procesos de desarrollo que vive núcleo de población con la finalidad de fortalecer o reforzar el camino que están recorriendo en el ejido o comunidad agraria.
Reconocer y hacer valer el conocimiento tácito del núcleo de población, dejar en claro que ambas partes aprenderán, uno de otro y que habrá un intercambio de conocimientos y experiencias acumuladas.

Involucrar a jóvenes y mujeres en el proceso de monitoreo comunitario porque pueden hacer la diferencia en la dinámica del comité sobre todo para la motivación, administración, uso de GPS y computadora que se requiere para el monitoreo de los recursos naturales.

Las necesidades del monitoreo deben surgir desde la comunidad y no se debe imponer, porque los ejidatarios e integrantes del comité nunca se apropiaran de ello y las actividades que se realizarán lo harán por compromiso.

Involucrar más instituciones gubernamentales afines como CONANP, SEMARNAT, SAGARPA, otras áreas de la misma CONAFOR, la gerencia estatal de CONAFOR, entre otras, para analizar, discutir y tomar acuerdos sobre el monitoreo comunitario.

Las Juntas Intermunicipales deben tener presentes las dolencias locales (diagnósticos situacionales) de los ejidos y comunidades de su área de intervención e involucrarse en el tema del monitoreo comunitario además de estar abiertos a otras posibilidades.

Los comités de monitoreo comunitario necesitan de un incentivo económico para motivar su participación y asegurar el sustento alimenticio de sus familias.

RECOMENDACIONES

Se presenta diversas recomendaciones con dos temas principales, con la acotación de que las presentes recomendaciones, son sólo del alcance del presente sub-producto y no reflejan las recomendaciones finales del proyecto. Con ello se presentan primero las gubernamentales que tienen que ver con acuerdos y estrategias; las segundas, que engloban aspectos de la vida comunitaria.

Gubernamentales

Integrar en medida de lo posible actores institucionales que participen en el tema de REDD+ principalmente ejes temáticos dentro de Conafor con la finalidad de nutrir la experiencia.

Promover espacios de difusión de los avances con actores involucrados con la finalidad de homogenizar información y sensibilización con respecto a la vida y dinámica comunitaria y cómo esta influye en la deforestación y degradación.

Integrar otros actores como las Secretarías de Desarrollo Rural de los órdenes estatal y municipal, a espacios de análisis y discusión del modelo de monitoreo local.

Promover dentro de algunas áreas de Conafor, en específico el personal de la Gerencia Estatal de Jalisco su inclusión y participación en el proceso, así como los promotores regionales con que cuenta dicha Gerencia.

Sobre lo local

En medida de lo posible que el trabajo con los ejidos que cuentan con otros asesores técnicos, promover su inclusión en el proceso participativo de monitoreo comunitario.

Contemplar la dinámica comunitaria y su conocimiento del medio, de sus recursos y su distribución en el territorio, como base para el monitoreo comunitario de los recursos naturales.

REFERENCIAS BIBLIOGRÁFICAS

- Consultoría Ambiental Mascota, S.C., (2012), *Informe final de actividades del **acompañamiento para el fortalecimiento de empresas forestales comunitarias** del ejido El Empedrado*, municipio de Mascota, Jalisco. Conafor.
- Consultoría Ambiental Mascota, S.C., (2012), *Informe final de la modalidad de apoyo **estudio de ordenamiento territorial comunitario**, realizado en el ejido El Empedrado*, municipio de Mascota, Jalisco. Conafor.
- Pérez Pérez, Isidro (2012) *Informe de actividades realizadas al 30 de noviembre del 2012 de la modalidad de apoyo **promotor forestal comunitario**, realizado en el ejido El Empedrado*, municipio de Mascota, Jalisco. Conafor.
- Pérez Pérez, Isidro (2012) *Informe final de actividades de la modalidad de apoyo **promotor forestal comunitario**, realizado en el ejido El Empedrado*, municipio de Mascota, Jalisco. Conafor.
- Consultoría Ambiental Mascota, S.C., (2012), *Informe final del desarrollo de actividades para la elaboración del **estudio de ordenamiento territorial comunitario** del ejido Santiago de los Pinos*, municipio de San Sebastián del Oeste, Jalisco. Conafor.
- Consultoría Ambiental Mascota, S.C., (2012), *Informe único y final de la modalidad de apoyo **comité de vigilancia participativa**, realizado en el ejido Santiago de los Pinos*, municipio de San Sebastián del Oeste, Jalisco. Conafor.
- García Robles, Antonio, (2011, julio), Primer informe parcial bimestral ***Promotor forestal comunitario local**, realizado en el ejido El Jorullo*, municipio de Puerto Vallarta, Jalisco. Conafor.
- García Robles, Antonio, (2011, agosto), Primer informe parcial bimestral ***Promotor forestal comunitario local**, realizado en el ejido El Jorullo*, municipio de Puerto Vallarta, Jalisco. Conafor.
- García Robles, Antonio, (2011, septiembre), Tercer informe parcial ***Promotor forestal comunitario local**, realizado en el ejido El Jorullo*, municipio de Puerto Vallarta, Jalisco. Conafor.
- García Robles, Antonio, (2011, octubre), Cuarto informe parcial bimestral ***Promotor forestal comunitario local**, realizado en el ejido El Jorullo*, municipio de Puerto Vallarta, Jalisco. Conafor.
- Servicios Agroforestales de Occidente, S.C., (2011), *Asesoría para el fortalecimiento de empresas forestales comunitarias, Informe único **Empresa Ecoturística "Sierra Madre Canopy Tours S.C. de R.L.**, realizado en el ejido El Jorullo*, municipio de Puerto Vallarta, Jalisco. Conafor.
- Servicios Agroforestales de Occidente, S.C., (2011), ***Comité de vigilancia participativa**, realizado en el ejido El Jorullo*, municipio de Puerto Vallarta, Jalisco. Conafor.
- Villareal Castañeda, Leonor, (2012), *Informe final **taller participativo de servicios ambientales** del ejido El Jorullo y Anexos*, municipio de Puerto Vallarta, Jalisco. Conafor.

- Agencia Española para la Cooperación Internacional y el Desarrollo (2013), Comp. Rafael González Franco de la Peza, "***Crónica de la creación y el Desarrollo de la junta Intermunicipal de Medio Ambiente para la gestión integral de la cuenca baja del Río Ayuquila***", *Conafor.*
- Agencia Española para la Cooperación Internacional y el Desarrollo (2013), Comp. Rafael González Franco de la Peza, "***La gobernanza Intermunicipal y la Implementación de mecanismos REDD+ a nivel Local***", *Conafor.*
- Vargas Guillén, Adalberto; Álvarez Pérez, Micaela; Cuesta, Irene. ***"Guía Didáctica para la Participación Local en programas de Servicios Ambientales".*** Ed. Fray Bartolomé de las Casas A.C.
- Torres Rojo, Juan Manuel; ***"Estudio de tendencias y perspectivas del Sector Forestal en América Latina Documento de Trabajo".*** Secretaría de Medio Ambiente y Recursos Naturales de México (SEMARNAT) Organización de las Naciones Unidas para la Agricultura y la Alimentación (FAO), Roma, Italia; 2004.
- ***"Situación de los Bosques del Mundo 2009"***, Organización de las Naciones Unidas para la Agricultura y la Alimentación (FAO), Roma, Italia; 2004. ISSN 1020-5721

Páginas de internet

- *Padrón Historial de Núcleos Agrarios* - http://phina.ran.gob.mx/phina2/
- Instituto Nacional de Estadística y Geografía - http://www.inegi.org.mx/
- Comisión Nacional Forestal - http://www.conafor.gob.mx

Legislación mexicana

- Ley Agraria.
- Constitución Política del estado de Jalisco.
- Ley Orgánica Municipal del Estado de Jalisco.
- Ley de Planeación para el Estado de Jalisco y sus Municipios.
- Ley de Información Pública del Estado de Jalisco y sus Municipios.
- Reglamento de Policía y Buen Gobierno, del municipio de San Sebastián del Oeste.
