# Supplementary material for: Moving from Measuring, Reporting, Verification (MRV) of Forest Carbon to Community Mapping, Measuring, Monitoring (MMM): Perspectives from Mexico
Source: PLoS One. 2016 Jun 14;11(6):e0146038. doi: 10.1371/journal.pone.0146038 (PMC4907456; doi:10.1371/journal.pone.0146038)
Supplement: S2 Appendix — (PDF) [file pone.0146038.s002.pdf]

2013

incidencia social
Asociación Civil

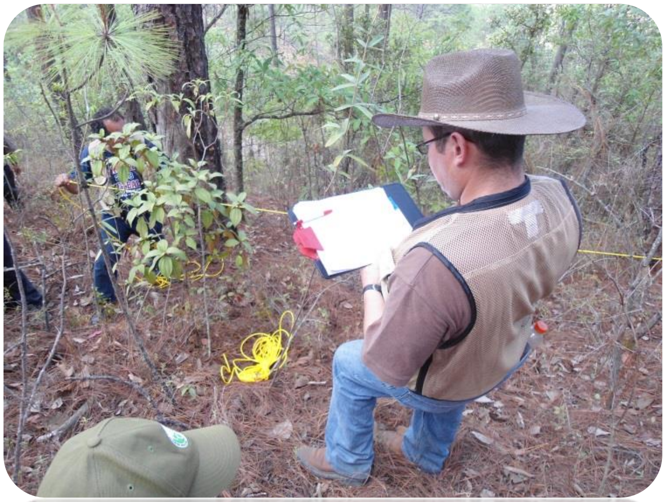

[LECCIONES APRENDIDAS Y BUENAS PRÁCTICAS]

Acompañamiento y Sistematización del Programa de Monitoreo Comunitario en Áreas Forestales que se encuentran en la Región de Cuencas Costeras de Jalisco.

AFD Agence Française de Développement · SEMARNAT Secretaría de Medio Ambiente y Recursos Naturales · CONAFOR Comisión Nacional Forestal · CONABIO Comisión Nacional para el Conocimiento y Uso de la Biodiversidad

incidencia social

Asociación Civil

Noviembre de 2013
Jalisco, México.

LECCIONES APRENDIDAS Y BUENAS PRÁCTICAS

* * * * * * * * * *

Acompañamiento y Sistematización del Programa de Monitoreo Comunitario en Áreas Forestales que se encuentran en la Región de Cuencas Costeras de Jalisco.

AGENCE FRANÇAISE DE DÉVELOPPEMENT · SEMARNAT SECRETARÍA DE MEDIO AMBIENTE Y RECURSOS NATURALES · CONAFOR COMISIÓN NACIONAL FORESTAL · CONABIO COMISIÓN NACIONAL PARA EL CONOCIMIENTO Y USO DE LA BIODIVERSIDAD

Lecciones Aprendidas y Buenas Prácticas

* * * * * *

Acompañamiento y Sistematización del Programa de Monitoreo Comunitario en Áreas Forestales que se encuentran en la Región de Cuencas Costeras de Jalisco

incidencia social

Asociación Civil

Textos e ideas:
Renato Ravelo Rodríguez, y
Camilo Tlacaelel Simancas Del Águila

Incidencia Social, A.C.
Calle: Altamirano No.15,
Col. Guerrero 200,
Chilpancingo de los Bravo,
Guerrero, México.
Tel.: +52 (747) 11609-44
E-mail: incide.guerrero@gmail.com

LECCIONES APRENDIDAS Y BUENAS PRÁCTICAS

Acompañamiento y sistematización del programa de monitoreo comunitario en áreas forestales que se encuentran en la región Cuencas Costeras de Jalisco.

Ubicación Geográfica:

Ejidos:

» Barranca del Calabozo, municipio de Pihuamo, Jalisco
» El Empedrado, municipio de Mascota, Jalisco
» El Jorullo y sus Anexos, municipio de Puerto Vallarta, Jalisco
» Santiago de los Pinos, municipio de San Sebastián del Oeste, Jalisco

Periodo de acompañamiento:

Junio del 2 mil 13 – Octubre del 2 mil 13

Lecciones aprendidas generales:

- Indudablemente se tienen que *respetar e identificar los esquemas de* **GOBERNANZA LOCAL** *y sus* **ESTRUCTURAS SOCIALES INTERNAS**, además de la enmarcada en la legislación agraria como los órganos de representación y vigilancia del ejido o comunidad agraria (asamblea general, comisariado y consejo de vigilancia), también existen comités locales que tienen cierta representación y peso en la toma de decisiones en las asambleas.

- Al inicio de cualquier proceso de desarrollo en los ejidos o comunidades agrarias se deben *identificar*, *respetar*, *dialogar* y *llegar a acuerdos consensuados* con los **ACTORES INTERNOS Y EXTERNOS** que intervienen en la vida de la comunidad, como ejemplo podemos encontrar los siguientes:

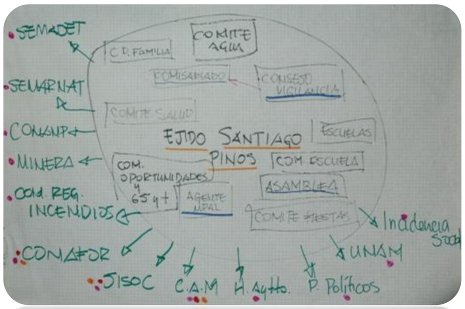

ACTORES INTERNOS	ACTORES EXTERNOS
• Comisariado	• Visitador agrario
• Consejo de vigilancia	• Asesores técnicos
• Autoridad auxiliar municipal	• Promotores institucionales
• Consejo de principales	• Instituciones gubernamentales
• Comités comunitarios	• Instituciones u organizaciones no gubernamentales
• Liderazgos naturales	• Juntas intermunicipales
• Promotores comunitarios	• Ayuntamientos
• Entre otros	• Entre otros

- Los actores externos debemos de respetar la **DINÁMICA COMUNITARIA** (tiempos, usos, costumbres y tradiciones) así como las reglas escritas y no escritas a las que dan obediencia en la comunidad, con el afán de consolidar y fortalecer la confianza con ellos. En algunos ejidos tienen por costumbre reunirse determinado tiempo, ya sea a inicios o finales de cada mes o cada dos meses o tener estipulado un día en específico a los que nos debemos acoplar y sólo por causas de fuerza mayor realizar una convocaría, si es necesario, para tocar los asuntos que nos conciernen, exclusivamente.

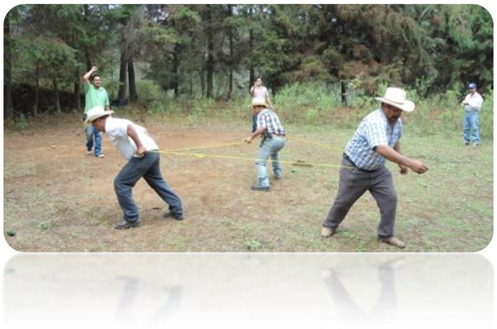

- Los actores externos que pretendan **CONTRIBUIR AL DESARROLLO DEL EJIDO** deben estar abiertos a otras posibilidades de facilitación, es decir, no enfocarse únicamente al asunto que nos atañe en ese momento, si está dentro de nuestra posibilidad también debemos contribuir u orientar la necesidad comunitaria.

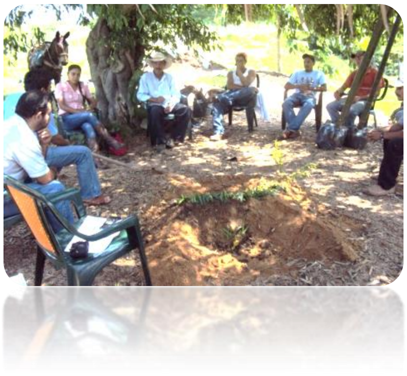

- De ser posible debemos **ENTRELAZAR LOS PROCESOS DE DESARROLLO** que vive núcleo de población con la finalidad de fortalecer o reforzar el camino que están recorriendo en el ejido o comunidad agraria.

- Reconocer y **HACER VALER EL CONOCIMIENTO TÁCITO** del núcleo de población, dejar en claro que ambas partes aprenderán, uno de otro y que habrá un intercambio de conocimientos y experiencias acumuladas.

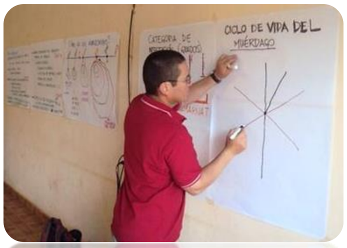

- **INVOLUCRAR A JÓVENES Y MUJERES** en el proceso de monitoreo comunitario porque pueden hacer la diferencia en la dinámica del comité sobre todo para la motivación, administración, uso de GPS y computadora que se requiere para el monitoreo de los recursos naturales.

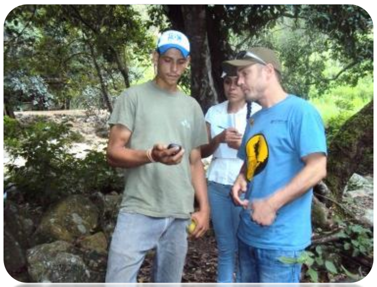

- Las necesidades del **MONITOREO DEBEN SURGIR DESDE LA COMUNIDAD** y **no** se debe imponer, porque los ejidatarios e integrantes del comité nunca se apropiaran de ello y las actividades que se realizarán lo harán por compromiso.

- **INVOLUCRAR MÁS INSTITUCIONES GUBERNAMENTALES** afines como CONANP, SEMARNAT, SAGARPA, otras áreas de la misma CONAFOR, la gerencia estatal de CONAFOR, entre otras, para analizar, discutir y tomar acuerdos sobre el monitoreo comunitario.

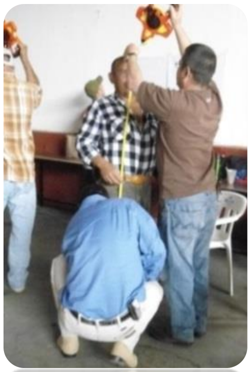

- Las **JUNTAS INTERMUNICIPALES** deben tener presentes las dolencias locales (diagnósticos situacionales) de los ejidos y comunidades de su área de intervención e involucrarse en el tema del monitoreo comunitario además de estar abiertos a otras posibilidades.

- Los comités de monitoreo comunitario necesitan de un **INCENTIVO ECONÓMICO** para motivar su participación y asegurar el sustento alimenticio de sus familias.

Lecciones aprendidas particulares:

La estrategia de intervención del proceso de monitoreo comunitario de cuencas costeras plasmada en la siguiente imagen nos dejó muchos aprendizajes los cuales se detallaran a partir de la misma, enfatizando en cada uno de ellos:

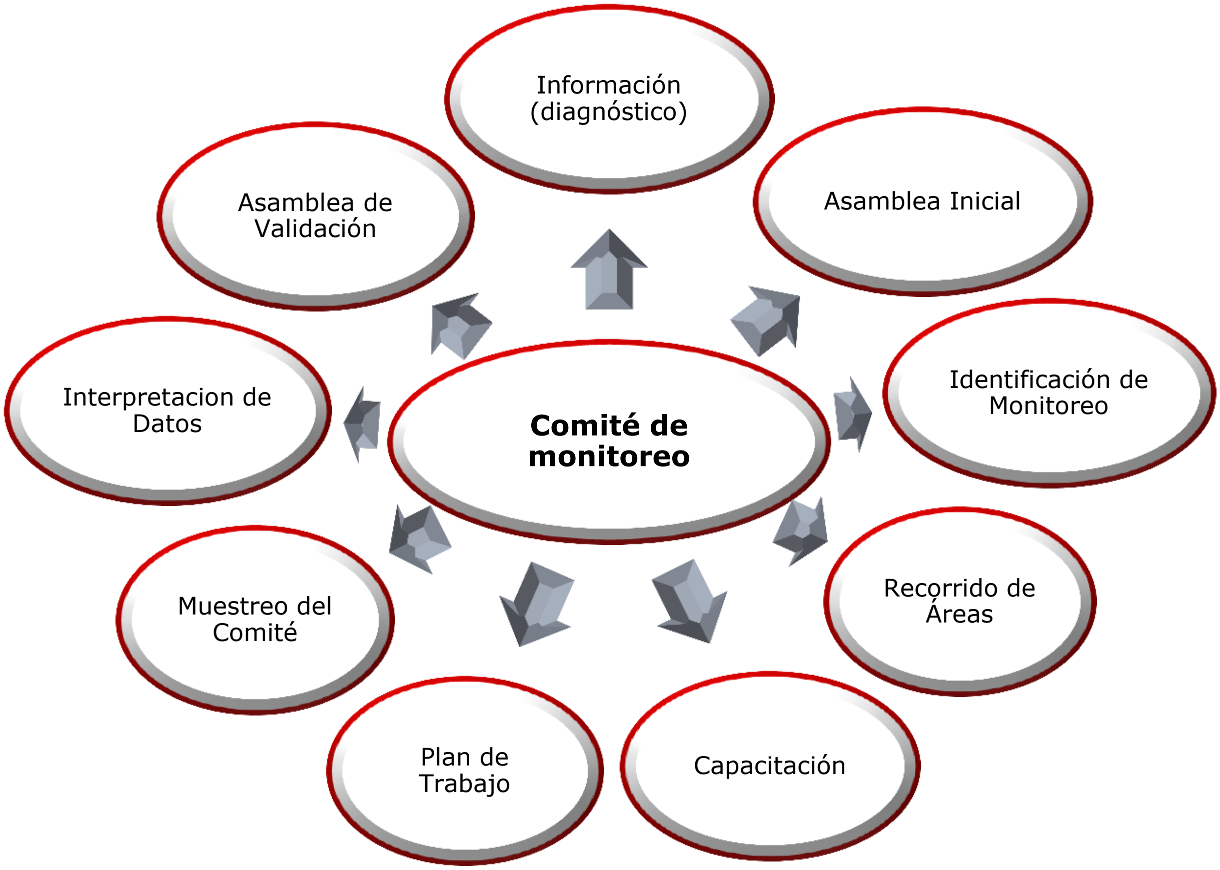

Cabe señalar que en algunas de las etapas del proceso de monitoreo comunitario pueden parecer repetitivos los ***posibles errores***, sin embargo cada una de las etapas son distintas, las cuales se abordan de manera diferente en las ***alternativas de atención para la posible solución***. Los posibles errores son variables porque pueden presentarse de manera diferente ya que cada ejido o comunidad agraria tiene dinámicas distintas.

EN EL DIAGNÓSTICO:

Posibles errores	Alternativas de atención para posible solución
Exclusión de actores externos que fomentan el desarrollo al interior del ejido o comunidad agraria	Durante el proceso del diagnóstico es importante analizar los actores internos como los externos del ejido o comunidad que tienen vínculos en el desarrollo del mismo como: *asesores técnicos*, *ayuntamientos*, *promotores institucionales*, *visitadores agrarios*, *juntas intermunicipales*, *entre otros*.<br>Sobre todo identificar en que procesos se involucran para poder considerar su participación en el desarrollo de este tipo de proyectos. Siendo necesario tener un acercamiento con estos para plantearles el proceso de monitoreo comunitario y si fuera posible que nos acerquen con las autoridades del ejido o comunidad y pedirles que estén presentes en la asamblea general si fuera posible.
Desfavorecer a las autoridades locales (comisariado y consejo de vigilancia), consejo de ancianos, principales o consejos consultivos de las autoridades y líderes	En este proceso se debe considerar aún más que los actores externos, cómo está integrado el ejido o comunidad agraria para que sean tomados en cuenta en el proceso de monitoreo comunitario y saber a quién nos dirigiremos para plantearles la idea del monitoreo de sus recursos solicitándoles que nos inviten a una asamblea general de ejidatarios o comuneros para plantearles el proyecto.
Depender de la información que nos proporcionen las instituciones a través de los informes de algunos proyectos que se hubiesen desarrollado en el ejido o comunidad agraria	En los ejidos y comunidades agrarias tienen dinámicas cambiantes (usos y costumbres) que no necesariamente están plasmados en los informes que se elaboran porque de un año a otro pueden cambiar, dependiendo sus necesidades locales, por ello debemos hacer preguntas dirigidas a los ejidatarios o comuneros, asesores técnicos, promotores, visitador agrario, entre otros, con el afán de conocer la dinámica del ejido o comunidad agraria.
Depender de la información solicitada a las instituciones de manera verbal	Será necesario hacer una solicitud de información pública a través de los mecanismos locales, estatales y federales como el Infomex, sólo que tendrá que hacerse con tiempo, esto con la necesidad de tener información confiable que nos ayude a entender a lo que nos enfrentaremos.
Depender de la información que nos proporciona una sola persona del ejido o comunidad agraria	Generalmente cada persona cuenta lo que mejor le convenga para beneficio de su ejido o comunidad; por ello necesitamos conocer diferentes puntos de vista de los actores locales para poder sacar conjeturas y cruzar la información con los documentos conseguidos.

EN ASAMBLEA INICIAL:

Posibles errores	Alternativas de atención para posible solución
Acudir a la asamblea general inicial sin tener una entrevista previa con las autoridades del ejido o comunidad agraria	Solicitar al presidente de la mesa de los debates que nos permita brindar una información al respecto, en la que no necesariamente planteemos dar inicio con las actividades, sino que sea informativa y programar otra asamblea en la que se incluya nuestra participación en el orden del día, al finalizar la asamblea será necesario platicar más a fondo con las autoridades (comisariado y consejo de vigilancia) sobre lo que se pretende con el monitoreo para poder identificar a que otros actores (asesores técnicos, instituciones, promotores, visitador agrario) podemos contemplar en el involucramiento del monitoreo comunitario y si es posible que los inviten a la próxima asamblea que se hubiese acordado.
Asistir al lugar de los hechos sin tener un panorama general del ejido o comunidad agraria	Identificar el nivel de organización y participación cuándo estemos ahí, por lo menos dejar clara la idea de lo que se pretende con el proceso de monitoreo comunitario, si nos permitieran participar; recomendando tener otra asamblea en lo posterior donde podamos también nosotros ordenar nuestra información a través de una presentación con diapositivas o bien sobre hojas rotafolio, preferentemente elaborar una ficha descriptiva o un plan de formación. Esto nos servirá para hacer un pre-diagnóstico del ejido o comunidad dónde identificaremos algunos de los actores locales de quienes debemos considerar. También nos dará pauta para disculparnos por nuestra repentina presencia y hacerles saber que respetamos sus usos y costumbres.
Apropiarse y tomar la dirección de la asamblea general.	Debemos aprender a percatarnos de lo que hacemos, sin duda alguna, al tomar la dirección de la asamblea nos daremos cuenta al ver el comportamiento de las mismas personas (inquietas, indiferentes, tímidas), inmediatamente debemos pasar la palabra al presidente de la mesa de los debates o presidente del comisariado. Sobre todo debemos ir con el pensamiento de que no somos parte del ejido o comunidad para que no suceda.
Desfavorecer a las autoridades locales	Siempre que hablemos en asamblea debemos referirnos a las autoridades del ejido o comunidad (comisariado y consejo de vigilancia) con mucho respeto y valorando su encargo. Si se trata de tomar los acuerdos dejarlo en manos de estos para que se organicen como acostumbran. Sólo intervenir cuando veamos que no haya claridad en los asuntos, nunca debemos arrebatar la palabra.

Posibles errores	Alternativas de atención para posible solución
Indicar únicamente la importancia del monitoreo comunitario	Al expresar únicamente la importancia del monitoreo comunitario ante la asamblea general también debemos dejar en claro cuáles son los beneficios de este para todos y no sólo para un grupo de persona. Esto dependerá de los recursos naturales con los que cuenten o de los proyectos que se ejecuten en el ejido o comunidad.
Imposición de los integrantes de los comités	No debemos excluir la participación de personas, sino todo lo contrario, porque entre más involucrados mejor serán nuestros resultados. Debemos dejar que la asamblea decida para que incluya a aquellas personas que deseen participar en el proceso, sean mujeres o jóvenes, aunque no necesariamente los jóvenes participan en la asamblea debemos dejar en claro que el involucramiento de los hijos e hijas de ejidatarios es importante también.
Cambiar las formas y costumbres de organización en el ejido o comunidad agraria	Al inmiscuirnos en ciertos procesos de desarrollo de los ejidos y comunidades, nos sentimos parte de ellas o bien queremos que se hagan las cosas a como nosotros queremos, sin embargo debemos dejar que se organicen y hagan las cosas a como lo acostumbran, podemos ejemplificar algunas cosas para mejorarlas, pero sólo hacerlas como recomendaciones, nunca como imposición de algo en lo que no tenemos por qué cambiarlo si no es el momento que ellos consideran.
Perspectiva de género	No debemos hacer menos las participación de las mujeres en los comités de monitoreo, seguramente habrá quienes quieran involucrarse porque se sienten comprometidas con el uso de los recursos naturales del territorio ejidal o comunal y son ellas quienes pueden ayudar a motivar la participación e involucramiento de más mujeres en el proceso. Recomendamos que en el diagnóstico se identifique hasta qué grado se involucran las mujeres en la vida de la comunidad y en la toma de decisiones en las asambleas.
Descartar la participación de jóvenes	No distinguir de ejidatarios e hijos de estos para el involucramiento de este proceso, seguramente habrá jóvenes que les interesa el desarrollo del ejido o comunidad, que lamentablemente no se les considera para las decisiones en las asambleas, muchas ocasiones no pueden entrar a estas, pero se puede dejar la tarea de buscar a quiénes puedan apoyar en el manejo del GPS y computadora, siendo los más indicados para esto.
Llegar tarde a un evento que se ha acordado con tiempo de anticipación	Si nos permitieran retomar el punto, tendremos que disculparnos por la demora, abordar el punto de manera rápida y concreta porque los hemos hecho esperar. Recomendamos si es un lugar lejano, estar un día antes o bien un par de horas.

EN LA IDENTIFICACIÓN DE MONITOREO:

Posibles errores	Alternativas de atención para posible solución
Acudir al taller de capacitación sin conocer el propósito o tener claridad de lo que vamos hacer	Muchas de las veces nos confiamos en lo que vamos hacer, pero si no tenemos claro el objetivo de lo que estamos haciendo y bien cimentada la metodología de lo que queremos lograr con los integrantes de los comités, los resultados serán igual, confusos. Por ello recomendamos que la planeación del taller para la identificación se aboque únicamente, a la identificación de los recursos naturales que yacen en el territorio de los ejidos o comunidades y determinar los beneficios si los monitoreamos, dejando en manos de los integrantes de los comités la priorización del monitoreo en coordinación con las autoridades del ejido o comunidad o bien en sus respectivas asambleas, dándonos pauta para determinar los instrumentos de medición que se utilizaran.
Cambiar las formas y costumbres de organización en la comunidad	Si como facilitadores determinamos el monitoreo a partir de lo que queremos saber, esto no tendrá el éxito deseado porque estaríamos imponiendo qué debemos monitorear, por ello es fundamental dejar a los integrantes de los comités de monitoreo lo determinen y consensen con sus autoridades o asambleas generales.
Desfavorecer a las autoridades locales (comisariado y consejo de vigilancia)	Si dejamos que la decisión del monitoreo únicamente los integrantes de los comités de monitoreo, estaremos menospreciando a las autoridades, corriendo el riesgo de enemistarnos con ellas, por ello es de vital importancia que se involucran en este proceso o en su caso los integrantes del comité acudan a ellos, dejando en claro, que nunca deben actuar sin considerar a sus autoridades porque fueron electas en asamblea general.
Indicar únicamente la importancia del monitoreo comunitario	No debemos enfatizar únicamente en la importancia que tienen el monitoreo de los recursos sino acentuar también en los beneficios ambientales, sociales y en su caso económicos que pueden traer, no sólo para los integrantes de los comités sino para todo el núcleo de población del ejido o comunidad agraria.
Capacitaciones expositivas, no participativas	Debemos emplear técnicas de educación popular (metodologías participativas), con la finalidad de que los integrantes de los comités de monitoreo comunitario determinen su propias propuestas y construyan colectivamente su desarrollo. Previo a cada capacitación o curso previsto debe traer consigo un plan de formación o una ficha descriptiva para poner en marcha, debiendo trabajarse con días de anticipación.

Posibles errores	Alternativas de atención para posible solución
Tomar decisiones sin consultar a la asamblea general	Una vez conocidas las necesidades del monitoreo y los beneficios que se obtendrán de ello, es preciso que la asamblea determine que monitorear. En su caso, tendremos que hacer que los integrantes analicen los pormenores o posibles discusiones que pudieran presentarse en la asamblea para poder determinar por si mismos el monitoreo a implementar, no es necesario que la asamblea valide este proceso pero también se le debe tomar en cuenta. Al final del proceso se realizará una asamblea general para que validen el proceso la cual debe ser obligatoria.

EN EL RECORRIDO DE ÁREAS:

Posibles errores	Alternativas de atención para posible solución
Asistir al lugar de los hechos sin tener un panorama general del territorio	Previo a realizar los recorridos de campo, es necesario que se identifiquen las áreas o zonas a visitar, si por algún motivo no hubiera tiempo se tendrá que preguntar a los integrantes de los comités cuales son las mejores rutas de acceso y tiempos en los que llegamos. De preferencia que los integrantes de los comités realicen unos mapas en hojas rotafilo para tener acceso a estos lugares.
Desfavorecer a las autoridades locales (comisariado y consejo de vigilancia)	Si los integrantes del comisariado o consejo de vigilancia no pudieran asistir o acompañar en los recorridos al finalizar estos, tenemos que buscarlos y decirles los lugares que visitamos para que se sientan incluidos en el proceso de monitoreo comunitario, donde también se especificará la fecha acordada para realizar una visita posterior y efectuar la capacitación para el manejo de instrumentos de medición.
Tomar decisiones sin consultar a la asamblea general	Dejar de tarea a los integrantes de los comités que consulten e informen a la asamblea general de los procesos por los cuales se han pasado y los pasos siguientes del monitoreo comunitario.

EN LA CAPACITACIÓN:

Posibles errores	Alternativas de atención para posible solución
Acudir al taller de capacitación sin conocer el propósito o tener claridad de lo que vamos hacer	Muchas de las veces nos confiamos en lo que vamos hacer, pero si no tenemos claro el objetivo de lo que estamos haciendo y bien cimentada la metodología de lo que queremos lograr con los integrantes de los comités, los resultados serán igual, confusos. Por ello recomendamos que la capacitación se enfoque a las necesidades del monitoreo de los recursos que cada comité y ejido o comunidad agraria hubiese determinado. Esta capacitación debe estar dirigida por personas que tengan experiencia en inventarios y monitoreos comunitarios preferentemente. Muy recomendable es que elaboren un plan de formación o por lo menos una ficha técnica para su puesta en marcha.
Desfavorecer a las autoridades locales (comisariado y consejo de vigilancia)	En esta parte de la capacitación se debe invitar a las autoridades del ejido o comunidad, con la finalidad de que entiendan de lo que se trata. En caso de olvidárseles será importante que los integrantes de lo comité informen de las actividades que se realizaron durante la capacitación.
Capacitaciones expositivas, no participativas	Debemos emplear técnicas de la educación popular (metodologías participativas), con la finalidad de que sean, los integrantes de los comités de monitoreo comunitario, quienes determinen su propias propuestas y construyan colectivamente su desarrollo, por ello recomendamos siempre traigan consigo manuales o archivos que nos permitan facilitar estos procesos, sin embargo esta etapa debe ser más práctica que teórica por el manejo de los instrumentos de medición.

EN LA ELABORACIÓN DEL PLAN DE TRABAJO:

Posibles errores	Alternativas de atención para posible solución
Cambiar las formas y costumbres de organización en la comunidad	Las actividades se realizarán de acuerdo a las posibilidades (dinámica local) de los integrantes de los comités. Imponer actividades y tiempos los desmoralizará, debemos siempre dejar que ellos inicien con las decisiones, y el papel del facilitador deberá ser este precisamente, facilitarlas.

**Posibles errores**	**Alternativas de atención para posible solución**
Indicar únicamente la importancia del monitoreo comunitario	Si sólo nos enfocamos a las actividades del monitoreo, ámbito que nos atañe, pueden ser valiosos los resultados, sin embargo hay ejidos o comunidades donde desconocen por completo las instituciones que pudieran apoyar al ejido o comunidad en diferentes actividades como la realización de obras de conservación de suelo, reforestación, aprovechamientos maderables y no maderables, etc. y será un buen momento para darlo a conocer. Los facilitadores deberán tener conocimiento de ello o en su defecto orientar a que a través de las autoridades locales gestionen pláticas o talleres para estar enterados de los conceptos de apoyo que las instituciones brindan, estas necesidades tendrán que plasmarse en el programa de trabajo.
Tomar decisiones sin consultar a la asamblea general	La construcción del plan de trabajo la realizan los integrantes de los comités de monitoreo comunitario y en su caso acompañan las autoridades del ejido o comunidad agraria, pero recordemos que las actividades que se realicen serán en territorio ejidal o comunal siendo indispensable que en una asamblea puedan conocerlas todos los ejidatarios o comuneros, para que, por lo menos estén informados.
Perspectiva de género	Debemos incluir las tareas de las mujeres y analizar como ellas participan en el aprovechamiento de los recursos naturales para poder hacer énfasis en que ellas también son parte del ejido o comunidad, sean o no ejidatarias o comuneras y destacar que si se involucran podremos tener mejores resultados.
Descartar la participación de jóvenes	En esta etapa es fundamental la participación de los jóvenes porque se programarán recorridos para el monitoreo y serán ellos quienes apoyen con el uso de instrumentos como el GPS y el SIG.
Imponer actividades para el monitoreo	Si creemos que hacemos bien imponiendo actividades para el plan de trabajo del comité, cometemos un error; sin embargo cuando lo hagamos y no nos percatemos de ello en el momento, hay que decir que es lo más viable si es que compaginan con la idea, en caso de que no, pues se tendrá que eliminar del plan de trabajo; de preferencia se tienen que ordenar las actividades.
Desfavorecer a las autoridades locales (comisariado y consejo de vigilancia)	Muchas de las veces, las actividades siempre recaen en las autoridades o representantes de los comités, sin embargo habría que hacer una distribución equitativa del trabajo porque se puede abarcar más de lo deseado. Invitar a las autoridades del ejido o comunidad dará oportunidad a realizar compromisos en beneficio del ejido o comunidad agraria.

Posibles errores	Alternativas de atención para posible solución
Apartar el involucramiento de las instituciones y juntas intermunicipales	Es indispensable que los representantes de las instituciones y juntas intermunicipales involucradas, acudan en esta etapa, porque pueden surgir actividades en las cuales pueden decidir sobre las necesidades que se enuncien en el plan de trabajo.

EN EL MUESTREO COMUNITARIO:

Posibles errores	Alternativas de atención para posible solución
Involucrarse demasiado en la práctica de los comités	El muestreo comunitario es una de las actividades donde se involucran directamente los integrantes de los comités para poner a prueba lo aprendido. Si nos involucramos demasiado no pondrán en práctica sus conocimientos. Debemos dejar clara nuestra función en esta etapa, en calidad de observador.
Recalcar que lo hicieron mal	Si en la práctica los integrantes de los comités cometieron errores no debemos decirles eso, sino todo lo contrario, recordándoles que el proceso de aprendizaje para esto es a base de prueba y error, destacando que si no nos percatamos de los errores siempre los estaremos cometiendo.
Desfavorecer a las autoridades locales (comisariado y consejo de vigilancia)	En este proceso es importante que se involucren las autoridades del ejido o comunidad para que se percaten del proceso de aprendizaje de los integrantes del comité, así como para que ellos también aprendan, por lo menos la utilidad de los instrumentos para el monitoreo.

EN LA INTERPRETACIÓN DE DATOS:

Posibles errores	Alternativas de atención para posible solución
Excluir a las autoridades del ejido o comunidad agraria	Es necesario que en esta etapa las autoridades del ejido o comunidad se involucren, porque los resultados que arrojen los datos recabados en campo ayudaran para percatarse de la cantidad, calidad, etc. de los recursos naturales que se tienen en el territorio, dependiendo de los que monitoreamos.

Posibles errores	Alternativas de atención para posible solución
No asociar los datos con algo que los integrantes del comité se relacionen	Generalmente las personas de mayor edad argumentan que les es complicado o que no saben de operaciones matemáticas básicas, sin embargo cuando hablamos de dinero o de cabezas de ganado o de cantidad de siembras no hay quien les gane, por así decirlo. Entonces para poder interpretar los datos recolectados en el muestreo es necesario asociarlo con palabras que estos conocen, plasmarlo en letras y números o bien con dibujos en hojas. Sin menospreciar las capacidades locales que tienen.
Tomar decisiones sin consultar a la asamblea general	Los resultados del monitoreo son de vital importancia, el que hacer con ellos no depende únicamente de los integrantes del comité ni de las autoridades del ejido o comunidad, por ello deben presentarse en asamblea general para determinar qué es lo que se tiene que hacer al respecto, proporcionando el comité diferentes alternativas.

EN LA ASAMBLEA DE VALIDACIÓN:

Posibles errores	Alternativas de atención para posible solución
Acudir a la asamblea sin tener claro el propósito	La asamblea general de validación social es para precisar las actividades que realizó el comité de monitoreo comunitario durante todo el proceso de integración y capacitación para el monitoreo comunitario de recursos naturales.
Apropiarse y tomar la dirección de la asamblea general	Recordemos que la realización de la asamblea general es para que se validen las actividades de los comités, por ello deberán ser los integrantes de estos, quienes realicen la presentación de los resultados y sólo intervenir cuando sea necesario.
Desfavorecer a las autoridades locales (Comisariado y Consejo de vigilancia)	Si nos percatamos que los integrantes del comité de monitoreo comunitario, se están apropiado de la dirección de la asamblea, será necesario intervenir y hacerles ver que la dirección la lleva el comisariado o bien el presidente de la mesa de los debates, según sea el caso; para que ellos, en caso de tomar acuerdos sean quienes lo sometan a consideración de acuerdo a sus usos y costumbres.
Descartar la participación de los actores externos del ejido o comunidad agraria	El involucramiento de las juntas intermunicipales, organizaciones no gubernamentales (locales, nacionales e internacionales), instituciones gubernamentales (ambientales, agrarias, sociales, entre otras), permitirá conocer las necesidades reales de los ejidos y comunidades del país en la que pueden apoyarse además de construir o mejorar políticas públicas, para percatarse de las necesidades locales.
