# Supplementary material for: Moving from Measuring, Reporting, Verification (MRV) of Forest Carbon to Community Mapping, Measuring, Monitoring (MMM): Perspectives from Mexico
Source: PLoS One. 2016 Jun 14;11(6):e0146038. doi: 10.1371/journal.pone.0146038 (PMC4907456; doi:10.1371/journal.pone.0146038)
Supplement: S3 Appendix — (PDF) [file pone.0146038.s003.pdf]

AGENCE FRANÇAISE DE DÉVELOPPEMENT

SEMARNAT SECRETARÍA DE MEDIO AMBIENTE Y RECURSOS NATURALES

CONAFOR COMISIÓN NACIONAL FORESTAL

CONABIO COMISIÓN NACIONAL PARA EL CONOCIMIENTO Y USO DE LA BIODIVERSIDAD

GUÍA PARTICIPATIVA PARA INTEGRAR Y CONSOLIDAR UN COMITÉ DE MONITOREO COMUNITARIO DE RECURSOS NATURALES

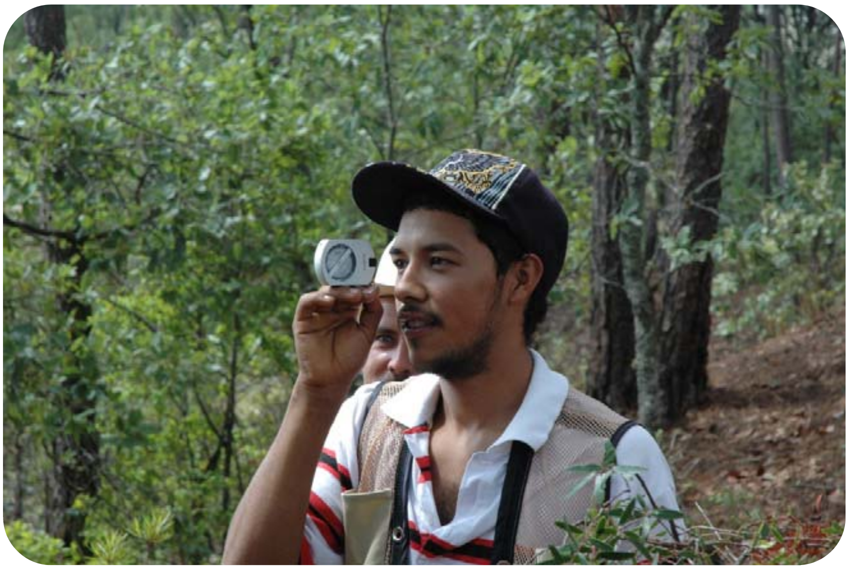

incidencia social

GUÍA PARTICIPATIVA PARA INTEGRAR Y CONSOLIDAR UN COMITÉ DE MONITOREO COMUNITARIO DE RECURSOS NATURALES

Noviembre de 2013
Jalisco, México.

Guía participativa para integrar y consolidar un comité de monitoreo comunitario de recursos naturales

incidencia social
Asociación Civil

Textos e ideas:
Renato Ravelo Rodríguez y
Camilo Tlacaelel Simancas Del Águila

Incidencia Social, A.C.
Calle: Altamirano No.15,
Col. Guerrero 200,
Chilpancingo de los Bravo,
Guerrero, México.
Tel.: +52 (747) 11609-44
E-mail: incide.guerrero@gmail.com

CONTENIDO

Presentación 5

Por qué vigilar y monitorear los recursos naturales de los ejidos y comunidades agrarias 7

Marco jurídico que promueve la participación social en la vigilancia de los recursos naturales 10

Comités comunitarios y el monitoreo de los recursos naturales 13

Aspectos a considerar para la conformación e integración del comité de monitoreo comunitario 16

Estrategia de intervención 21

- Diagnóstico local comunitario 22
- Asamblea general inicial 24
- Identificación y priorización del monitoreo 27
- Recorrido de áreas a monitorear 30
- Capacitación 32
- Plan de trabajo comunitario 38
- Muestreo comunitario 40
- Interpretación de datos 41
- Asamblea general de validación social 43

Reflexiones finales 45

- Recomendaciones bibliográficas y legislativas 47

PRESENTACIÓN

Esta guía tiene el propósito de mostrar los pasos para la integración y funcionamiento, con amplio consenso social, de un comité de monitoreo comunitario, con el apoyo del más alto órgano de toma de decisiones en los núcleos agrarios, para que pueda cumplir eficazmente las actividades del monitoreo de sus recursos naturales en beneficio de su ejido o comunidad agraria.

Se trata de un compendio de información relacionada con el proyecto piloto "Instrumentación y sistematización de la experiencia de monitoreo comunitario de recursos naturales en Jalisco" auspiciado por la Agencia Francesa de Desarrollo a través del Fondo de Inversión para Latinoamérica (LAIF) en México, siendo la Comisión Nacional Forestal (CONAFOR) y la Comisión Nacional para el Conocimiento y Uso de la Biodiversidad (CONABIO), las dependencias operativa y administrativa, respectivamente.

La finalidad de este trabajo está relacionada con la reproducción de la experiencia del establecimiento de comités de monitoreo comunitario en cuatro ejidos localizados en la región de cuencas costeras del estado de Jalisco, a efecto de que puedan ser replicables en otros ejidos y comunidades agrarias en la tarea de monitorear sus recursos naturales de sus respectivos territorios.

Primero debemos reconocer que el trabajo de los comités de monitoreo comunitario son un insumo importante para la toma de decisiones en cuanto al manejo y aprovechamiento de los recursos naturales, al mismo tiempo los integrantes de estos comités son interlocutores responsables de presentar datos sobre el comportamiento de sus bosques, selvas, agua, plagas, enfermedades, entre otros, a las asambleas generales de ejidatarios o de comuneros, quienes con información verídica y objetiva pueden tomar decisiones sobre la circunstancias que debieran realizarse como los aprovechamientos maderables y no maderables, creación de empresas sociales, saneamientos, nuevas inversiones, ampliación de los programas de manejo, entre otras.

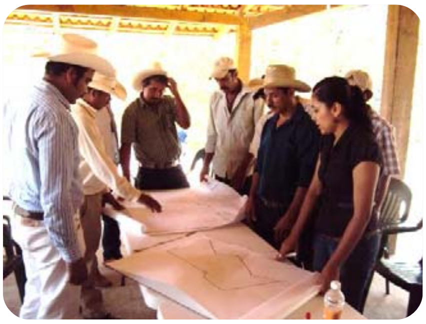

Las actividades relacionadas con el monitoreo de los recursos naturales en cada ejido o comunidad agraria implican la inversión de tiempo, porque constantemente se debe de monitorear el comportamiento de los recursos naturales, a través del uso de instrumentos como: las cuerdas compensadas, cinta diamétrica, cinta métrica, clinómetro, taladro de Pressler, GPS (Geo-Posicionador Satelital) y el SIG (Sistema de Información Geográfica); pero no hay un esquema de apoyo económico establecido a la fecha que pueda retribuir el tiempo y esfuerzo que los integrantes de los comités de monitoreo comunitario invierten.

El uso de los instrumentos para realizar el monitoreo debe enseñarse de manera sencilla, es decir con un lenguaje entendible, porque en la mayor parte de la población de los ejidos y comunidades agrarias del país no tienen un alto grado de escolaridad y los términos académicos, utilizados por los asesores técnicos, en la mayoría de los casos son de difícil entendimiento, recomendando pues, recurrir al uso de técnicas de educación popular en la impartición de cursos y talleres formativos.

Con esta guía buscamos contribuir en la conformación, integración, capacitación en uso de instrumentos, interpretación y manejo de datos así como su validación social de estos ante la máxima autoridad de los ejidos o comunidades agrarias: las asambleas generales; proporcionándoles información básica que creemos es de gran utilidad para ello; pero también es un documento para la reflexión con el que se busca propiciar mayor sensibilidad en el monitoreo de sus recursos naturales.

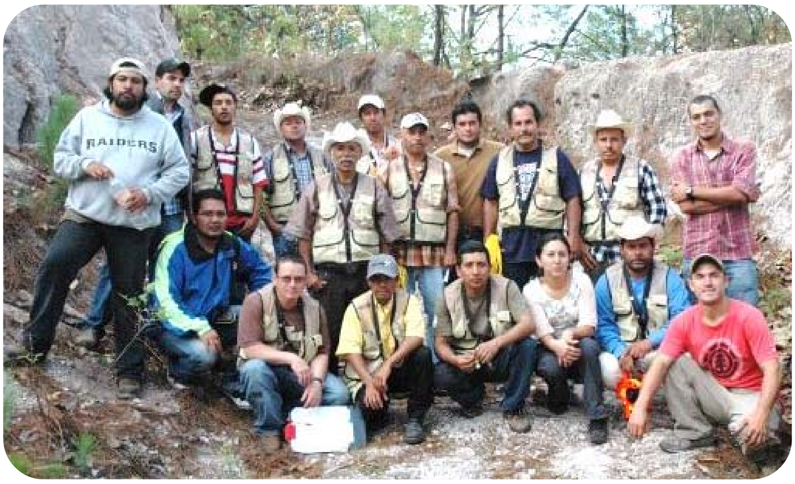

POR QUÉ MONITOREAR LOS RECURSOS NATURALES DE LOS EJIDOS Y COMUNIDADES AGRARIAS

Los ejidos y comunidades en México cuentan con diversos recursos naturales como la flora y fauna silvestre en el interior de sus territorios; estos forman ecosistemas locales contribuyendo a la regeneración natural o por medio de un manejo sustentable, de servicios ambientales (captación y filtración de agua, generación de oxígeno, protección de la biodiversidad, retención de suelo, refugio de fauna silvestre, belleza escénica, entre otros) para beneficio de la humanidad, ya sea a nivel local o regional.

Los núcleos agrarios tienen componentes internos distintos, es decir la manera de atender y entender la naturaleza es diferente, porque algunos aprovechan los recursos de manera sustentable, mientras que otros lo hacen sin ninguna racionalidad, esto se debe a la falta de sensibilización y concientización de la población, porque creemos que a estas alturas todos los mexicanos estamos conscientes de la importancia de estos recursos naturales, sin embargo se presentan factores sociales, económicos y culturales que nos orillan a aprovecharlos sin ningún control, un claro ejemplo es la falta de empleo conllevando los bajos ingresos económicos para el sustento familiar; buscando una salida fácil recurrimos al uso de los recursos naturales (árboles para leña y preparar alimentos, animales silvestres para cocinarlos y alimentar a la familia), volviéndose pues una costumbre que se ha venido heredando que no limitamos hasta que se presenta la escases o desaparición de estos recursos. En la práctica podemos percatarnos de esto porque cada vez tenemos que caminar más lejos para tener acceso a la leña, y para encontrarnos un animal silvestre (alimento) es baja la posibilidad; lo mismo pasa con el agua que antes era abundante, escaseándose en estos tiempos.

Hay ejidos y comunidades agrarias que no hace mucho tiempo se han organizado para realizar un manejo y aprovechamiento sustentable de sus recursos naturales, siendo factor fundamental para la generación de empleos para el uso sustentable de sus recursos naturales (maderables y no maderables), además ha disminuido la migración fuera de su territorio. No ha sido fácil para muchos núcleos agrarios llegar hasta donde están, nos atrevemos a decir que han pasado por lo menos una década que implicó un arduo trabajo de sensibilización y concientización, presentándose así problemas (menores) al respecto.

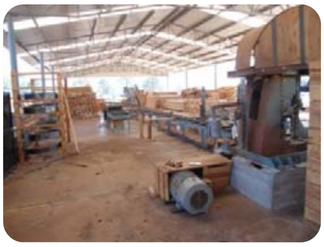

Los núcleos agrarios conscientes de la problemática ambiental adquieren otros compromisos y necesidades, sí su visión de desarrollo comunitario está enfocada al aprovechamiento sustentable de sus recursos naturales, están obligados a saber qué y cuántos recursos naturales están en mi territorio, porque sí quiero vender, por ejemplo, debo saber qué y cuál es su calidad, entre otras cosas para poder tomar una decisión sobre lo que puedo hacer. En caso de que los árboles tengan plaga o estén enfermos, se pueden contar, si fuera necesario hacer derribos sabríamos cuántos tendríamos que tirar; en el caso del agua también se puede cuantificar para saber cuánta atraviesa por los causes y cuánta se filtra en el territorio, así como el nivel de contaminación de la misma.

Hacer un inventario de lo que tenemos, es el primer paso para poder monitorear los recursos naturales que queramos, por ejemplo:

Tipo de vegetación
Árboles enfermos
Árboles plagados
Árboles secos
Árboles para aprovechar madera
Especies de arboles
Arboles nuevos
Arboles jóvenes
Arboles adultos

Árboles para semilla
Arboles viejos
Crecimiento de árboles
Cantidad de agua
Calidad de agua
Contaminación de agua
Especies de fauna silvestre
Cantidad de fauna silvestre
Usos de plantas y animales

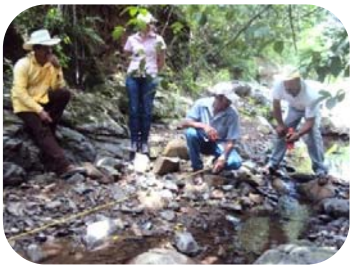

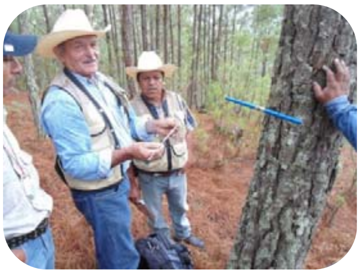

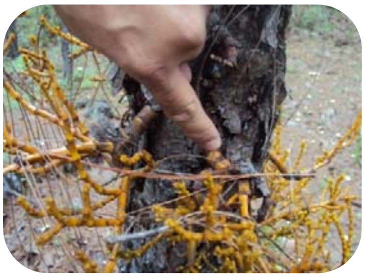

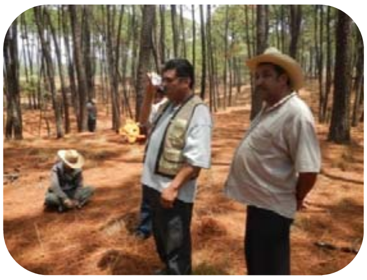

Si con el inventario nos damos cuenta de lo que tenemos en nuestro territorio, con el **monitoreo** sabremos los cambios que se presentan en ellos para que en asamblea general, se puedan decidir las mejores opciones para realizar un buen manejo de nuestros recursos naturales.

El inventario nos sirve, entre otras cosas para conocer información de interés para el ejido o comunidad agraria, como las especies de plantas y animales, cuáles son comestibles o medicinales, la cantidad de madera, ramas muertas que pueden ser combustible de posibles incendios o que se puede aprovechar como leña, entre otras más…

MARCO JURÍDICO QUE PROMUEVE LA PARTICIPACIÓN SOCIAL EN LA VIGILANCIA DE LOS RECURSOS NATURALES

Durante muchos años en México se ha incluido el tema de la conservación de los recursos naturales; sin embargo, recientemente han tenido auge por las condiciones climatológicas que se presentan actualmente en el territorio mexicano (escases de lluvias, calor, plagas y enfermedades en los árboles, erosión de suelos, entre otras), ante estos fenómenos, previendo condiciones extremas en el ambiente se ha facultado la participación ciudadana y social para mitigar estos fenómenos. Por ello es importante resaltar el marco jurídico mexicano que promueve la participación para la conservación de los recursos naturales:

En la **Constitución Política de los Estados Unidos Mexicanos** se establece el derecho de toda persona a un ambiente adecuado para su desarrollo y bienestar (artículo 4). Por otra parte, la misma carta magna sienta las bases para la participación de la sociedad en el desarrollo, con el afán de que sea integral y sustentable, así como en su planeación democrática (artículo 25 y 26).

En la **Ley General del Equilibrio Ecológico y la Protección al Ambiente** (LGEEPA), se establece que su objeto es propiciar el desarrollo sustentable para establecer las bases para garantizar la participación corresponsable de las personas en forma individual o colectiva, en la preservación, restauración del equilibrio ecológico y la protección al ambiente (artículo 1).
Establece los criterios específicos de participación en materia de impacto ambiental (artículo 34), áreas naturales protegidas (artículo 56 bis), ordenamiento ecológico del territorio y, de manera acentuada, establece el mecanismo de denuncia ambiental, mediante el cual toda persona, grupos sociales, organizaciones no gubernamentales, asociaciones y sociedades podrán denunciar ante la Procuraduría Federal de Protección al Ambiente (PROFEPA) o ante otras autoridades, todo acto que produzca o pueda producir desequilibrio ecológico o daños al ambiente o a los recursos naturales (artículo 189).

El **reglamento de la LGEEPA** en materia de áreas naturales protegidas establece, en el Capítulo IV, los criterios para regular la participación social en las ANP's, mediante Consejos Asesores.

La **Ley General de Vida Silvestre y su reglamento** establecen disposiciones legales para promover e inducir la participación social en los programas de conservación y aprovechamiento de la vida silvestre.

La **Ley General de Desarrollo Forestal Sustentable y su reglamento**, cuyo objeto es regular, fomentar la conservación, protección, restauración, aprovechamiento, manejo, cultivo y producción de los recursos forestales del país, a fin de propiciar el desarrollo sustentable, establece que la política forestal tendrá como propósito incrementar la participación corresponsable de la sociedad en la protección, conservación, restauración y aprovechamiento sustentable de los recursos forestales (artículo 1); prevé que los acuerdos y convenios que en materia forestal celebre la Secretaría de Medio Ambiente y Recursos Naturales con personas físicas o morales del sector social o privado podrán versar, entre otras materias, en las labores de vigilancia forestal (artículo 8).

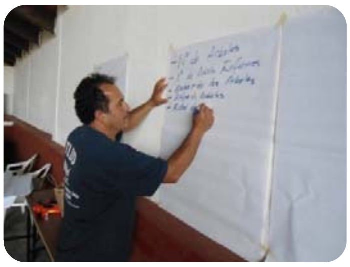

La **Ley General de Cambio Climático**, tiene como principal objetivo garantizar el derecho a un medio ambiente sano y establecer la concurrencia de facultades de la federación, las entidades federativas y los municipios en la elaboración de políticas públicas para la adaptación al cambio climático y mitigación de emisiones de gases y compuestos de efecto invernadero; pero también establecer las bases para la concertación con la sociedad y elaborar e instrumentar su programa en materia de cambio climático, promoviendo la participación social, escuchando a los sectores público, privado y sociedad en general.

El **Plan Nacional de Desarrollo**, el **Programa de Medio Ambiente y Recursos Naturales** y el **Programa de Procuración de Justicia Ambiental**, establecen políticas públicas para fomentar la participación social en el cuidado y conservación del medio ambiente, los recursos naturales, específicamente en la vigilancia e inducción del cumplimiento de la normatividad ambiental.

En este tenor, la PROFEPA tiene facultades y atribuciones para fomentar los mecanismos que garanticen la participación de los

individuos interesados en coadyuvar con la autoridad en acciones de conservación, preservación, restauración y manejo sustentable de los recursos naturales.

Como se puede observar, son variadas las normas que reconocen el derecho a participar, en coordinación con las autoridades ambientales (PROFEPA) para cuidar, conservar, proteger y aprovechar sustentablemente los recursos naturales de nuestro entorno con el afán de vivir en armonía con un ambiente adecuado para nuestro bienestar.

COMITÉS COMUNITARIOS Y EL MONITOREO DE RECURSOS NATURALES

Los comités comunitarios están integrados por personas que pertenecen a una comunidad, estas atienden diferentes necesidades o asuntos locales, son nombrados en la mayoría de los casos, por las personas que habitan la comunidad en asamblea general. Hay casos en los que se auto-proponen en otros son propuestos, esto porque, en el primer caso, además del compromiso moral, es una persona que le afecta y cree que su participación beneficiará a todos; en el segundo caso, necesitan sentir del apoyo de la comunidad y deciden no auto-proponerse, sin embargo, las personas que los proponen saben de la capacidad y responsabilidad de la persona. Generalmente estos comités tienen el compromiso de analizar la problemática local para mejorar las condiciones de vida de los habitantes de la comunidad.

Algunos comités comunitarios más comunes que se conforman en cualquier población de los ejidos y comunidades agrarias son:

COMITÉ	OBJETIVO
Comité de padres de familia	Atender las necesidades de las escuelas de la comunidad y coordinarse con los maestros y autoridades municipales en su comunidad...
Comité de Salud	Contribuir a que los centros o casas de salud en las comunidades funcionen correctamente…
Comité de Oportunidades	Coordinar acciones de participación comunitaria que beneficien a la misma comunidad y asegurar la permanencia de las y los beneficiarios con la finalidad de obtener apoyos para garantizar los estudios de los hijos de los padres de familia…
Comité de obras	Vigilar la adecuada y oportuna construcción de las obras públicas para su comunidad…
Comité de agua	Custodiar la distribución del agua en las viviendas de la comunidad así como la tubería de esta y en caso de que haya anomalías repararlas en coordinación con la autoridad local y los habitantes del poblado…
Comité de festividades	Organizar y realizar los preparativos para las fiestas de su comunidad…

Vale la pena resaltar que el funcionamiento de los comités es de carácter honorífico, es decir no reciben apoyo o estímulos económicos para su funcionamiento.

Recientemente en los ejidos y comunidades agrarias de México, con la finalidad de contribuir al cuidado, conservación y aprovechamiento sustentable de los recursos naturales de sus territorios, se ha incursionado en la conformación de comités de vigilancia ambiental

participativa (red vigía), promovidos por la Procuraduría Federal de Protección al Ambiente (PROFEPA) y por la Comisión Nacional Forestal (CONAFOR); la integración de éstos en ambos casos son de carácter honorífico, porque el apoyo de ambas dependencias son canalizados para capacitar a las personas que de manera voluntaria han decidido formar parte de éstos. La capacitación se basa en el conocimiento de la normatividad ambiental que regulan el aprovechamiento de los recursos naturales en su caso hacer las denuncias correspondientes contra quienes afecten de manera clandestina o ilegal daños al ambiente.

El esquema ideal para conformar el **comité de monitoreo comunitario** de recursos naturales, es que se utilice el que ya existe (comité de vigilancia ambiental participativa), en los ejidos donde hay, porque podrían integrarse comités y más comités en los cuales se podrían duplicar actividades pudiendo confrontar al mismo ejido o comunidad agraria y fracturar su tejido social. Para el caso de la conformación de nuevos comités de monitoreo comunitario se hacen las recomendaciones en el siguiente apartado.

La actuación del **comité de monitoreo comunitario** de recursos naturales está enfocado principalmente al manejo territorial sustentable, donde surgen beneficios ambientales y económicos, además de que proporciona datos reales de los recursos naturales que pueden servir al ejido o comunidad agraria para asimilar y dirigir los datos hacia el aprovechamiento de sus recursos naturales. Para poder lograr esto, es de vital importancia que los actores locales estén capacitados adecuadamente, pero a la vez se tiene que contemplar la validación social de las asambleas generales. Quienes dirijan las capacitaciones tienen que respetar los usos y costumbres, así como las estructuras locales de gobierno (sistemas de cargos locales), sin sobre pasar a quienes en asamblea general han decidido que los represente (comisariado).

Cada ejido o comunidad agraria presenta una problemática específica, es decir no podemos indicar que son las mismas, porque:

- Puede variar su ecosistema y tipo de vegetación;
- Cada ejido o comunidad agraria tiene su cosmovisión para el aprovechamiento de los recursos naturales;
- Las estructuras de gobernanza local no funcionan de la misma manera;
- Los liderazgos comunitarios no siempre radican en los comisariados;

No se comparten las mismas opiniones para el aprovechamiento sustentable de sus recursos naturales;
No tienen el mismo avance en su desarrollo comunitario;
Entre otras…

Entonces la sensibilización y concientización son factor preponderante para iniciar un esquema de este tipo (monitoreo comunitario) con el afán de hacerles ver, además de la importancia de los recursos naturales, los beneficios que estos pueden brindar a la población del núcleo agrario. Asimismo, está la capacitación para la integración del comité e ir afinando desde la planificación hasta la obtención de resultados para la toma de decisiones en colectivo (asamblea general).

ASPECTOS A CONSIDERAR PARA LA CONFORMACIÓN E INTEGRACIÓN DEL COMITÉ DE MONITOREO COMUNITARIO

En los ejidos y comunidades agrarias del país, cualquier iniciativa de desarrollo sea social, económico, ambiental o productiva inevitablemente debe considerar el componente de formación y capacitación como un ingrediente necesario para generar capacidades que permitan a los pobladores tomar en sus manos el curso de su propio desarrollo.

Sin embargo, se presenta como un gran reto el diseño y despliegue de acciones dirigidas al desarrollo de capacidades de la población de ejidos o comunidades agrarias, dado el perfil de sus habitantes: personas adultas, con altos índices de analfabetismo o analfabetismo funcional, y con dificultades para el aprendizaje conceptual.

Una de las claves para obtener resultados positivos en una estrategia de capacitación, es que las actividades sean ampliamente participativas y acordes con el perfil de los capacitados. Para ello se deben emplear técnicas de la educación popular, que es una propuesta metodológica pertinente para el trabajo de facilitación de procesos de desarrollo comunitario con el que es posible ir desarrollando alternativas de superación, construyendo colectivamente oportunidades para avanzar a partir de las realidades que viven los ejidatarios o comuneros.

Por ello, consideramos que los aspectos que son de suma importancia para conformar e integrar un comité de monitoreo comunitario deben estar basado en tres ejes fundamentales, sin limitar otras actividades que contribuyan a su acompañamiento y fortalecimiento:

1. **Revisión de información documental (**diagnóstico**).** Con esto podremos obtener un panorama general de la situación del ejido ¿A qué nos enfrentamos?

2. **Asambleas generales.** Son pertinentes para que los ejidatarios o comuneros conozcan el proceso que se realizará, haya involucramiento general, por lo menos informativo, y puedan tomar decisiones para beneficio de su núcleo agrario.

3. **Talleres participativos.** Como parte de las actividades de los talleres se deben considerar por lo menos los tres momentos clave del aprendizaje:

a) Primer momento. Recapitulación de aprendizajes anteriores ¿Qué se sabe del tema?
b) Segundo momento. Análisis y profundización del tema
c) Tercer momento. De lo aprendido, ¿qué podemos aplicar en nuestra realidad?

La **revisión de la información documental**, es importante porque así sabremos las condiciones del lugar que visitamos y podemos tener antecedentes de desarrollo comunitario, aunque es difícil encontrar documentos en internet, hay medios que nos permiten solicitar legalmente este tipo de información, en caso de que el ejido o comunidad agraria no los tenga, podemos recurrir a la herramienta del Infomex, esta nos permite solicitar cualquier tipo de información pública con base a la Ley Federal de Transparencia y Acceso a la Información Pública y Protección de Datos Personales.

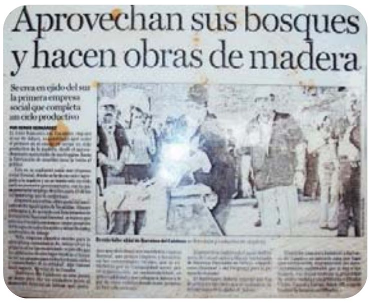
Aprovechan sus bosques y hacen obras de madera

Recomendamos en primera instancia, solicitar a las diferentes dependencias cuáles son los conceptos de apoyo y proyectos que se han ejecutado en cualquier núcleo agrario, para de ahí poder solicitar dichos informes de los proyectos realizados. A su vez será importante realizar un pequeño documento de diagnóstico el cual nos dé un panorama general de lo que se pretende realizar en el núcleo agrario.

Las asambleas generales de ejidatarios o comuneros son el máximo órgano de decisión sobre cualquier asunto relacionado con el bienestar de la población de cada núcleo agrario, en esta se pueden aceptar o rechazar propuestas de cualquier índole. Por eso consideramos que la integración del comité de monitoreo comunitario debe surgir en éstas, siendo pertinente darle a conocer el propósito y proceso que se llevará a cabo con los integrantes del comité de monitoreo para que haya un involucramiento general, por lo menos informativo, porque es la etapa inicial del proceso y es un buen momento en el que se pueden auto-proponer y/o proponer las personas (hombres y mujeres) que sean gustosas de involucrarse, sugiriéndoles antes el perfil más apropiado para ello:

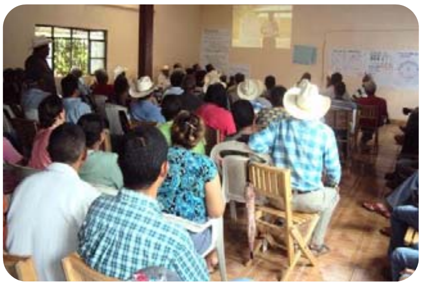

Saber leer, escribir y desarrollar operaciones n[illegible]
Conocimiento de la problemática local.
Conocimiento del territorio y accesos.
Ser reconocido en el ejido o comunidad.
Ser una persona honesta, responsable y comprometida.

La primera recomendación del perfil de los integrantes del comité de monitoreo **no** es necesario que todos la cumplan, esto porque sabemos que no todas las personas tuvieron las mismas oportunidades educativas, sobre todo las personas de mayor edad, que son quienes conocen en su totalidad o la mayor parte del territorio (límites, colindancias e historia) aunado a las características generales. Las personas adultas, en los ejidos o comunidades en México, cuentan con altos índices de analfabetismo o analfabetismo funcional y con dificultades para el aprendizaje conceptual.

También se debe considerar la participación de las mujeres en este tipo de comités porque son muchas las que son gustosas de involucrarse en el desarrollo de su ejido o comunidad, demuestran interés en ello, por lo que sugerimos no se les haga de lado y se debe de tocar el tema mientras se haga la presentación, es decir debemos ser incluyentes en este aspecto.

Por otra parte, la participación de jóvenes (hijos de ejidatarios o comuneros, o bien de avecindados) en este comité, es igual de importante porque son los que tienen un mayor acercamiento con la tecnología y pueden apoyar en estos aspectos (GPS –Sistema de Posicionamiento Global- y SIG –Sistema de Información Geográfica-) al comité de monitoreo comunitario.

Los **talleres participativos** tienen el principio básico del aprendizaje colectivo, pueden realizarse

En la experiencia vivida con la realización del proyecto, se tuvo el involucramiento de una **mujer** que le interesó el monitoreo comunitario y en asamblea solicitó ser parte del comité de monitoreo. Los integrantes y comisariado ejidal **de El Jorullo y Anexos** la nombraron representante del mismo porque su entusiasmo e interés por tratar de resolver una problemática general del núcleo agrario fue evidente.

En el caso de los **jóvenes** también fue necesario el involucramiento de los hijos de algunos ejidatarios de **Barranca del Calabozo**, porque se utilizaron herramientas como el GPS y el manejo de computadora para el SIG y fueron ellos quienes además de involucrarse en tareas de monitoreo (medición) fueron capaces de hacer un polígono y señalar la ubicación de su territorio, las cuales se presentaron en la asamblea general.

mediante el uso de diferentes técnicas, como las de educación popular:

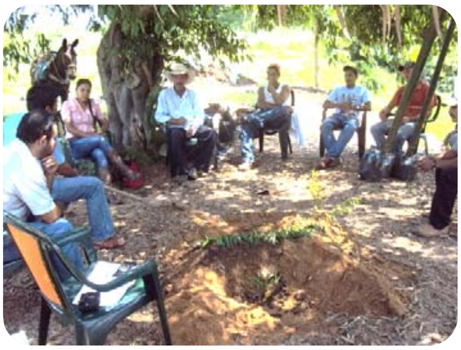

- Dinámicas de presentación;
- Dinámicas de animación;
- Exposiciones docentes;
- Exposiciones dialogadas;
- Ejercicios prácticos;
- Análisis y reflexión de temas en grupos o equipos;
- Recorridos de campo;
- Video debates;
- Lluvias de ideas;
- Entre otros.

Esta metodología es pertinente para el trabajo de facilitación de procesos de desarrollo comunitario, con el que es posible ir desarrollando alternativas de superación, construcción colectiva de oportunidades para avanzar partiendo de las realidades que viven los ejidatarios o comuneros.

Por otra parte, el diseño de una ficha descriptiva o plan de formación es una herramienta que facilita el desarrollo de cualquier actividad que tenga que ver con la formación porque es un instrumento flexible que evoluciona con el aprendizaje mutuo, porque:

- Posibilita reprogramar acciones y replantear estrategias al formador, pero también al formado;
- Concreta los objetivos en resultados de aprendizaje;
- Permite observar la evolución de la acción formativa a partir de evidencia;
- Apoya los procesos de evaluación y autoevaluación;
- Entre otras.

Esta ficha o plan de formación, en su elaboración debe considerar como mínimo, los siguientes elementos:

- Día, horario y duración;
- Actividades y temas;
- Propósito general del aprendizaje;
- Métodos y técnicas de enseñanza;
- Materiales didácticos;
- Método de evaluación; y
- Productos esperados.

En términos generales, debemos considerar lo siguiente para poder realizar un trabajo eficiente:

- Identificar los esquemas de gobernanza local (sus estructuras internas).
- Identificar y respetar las distintas modalidades de regulación internas como las tradiciones, costumbres, conocimientos, arreglos, acuerdos y normas, en específico las relativas al acceso a los recursos comunes y al usufructo de la tierra.
- Identificar actores, tanto internos como externos, que inciden en la toma de decisiones (estructuras e instituciones comunitarias, liderazgos, grupos vulnerables, estructuras de poder, asesores técnicos, instituciones gubernamentales).
- Considerar el intercambio de conocimientos y experiencias acumulados en las comunidades.
- Identificar la capacidad de adaptación como parte de su dinámica histórica.
- Estar sensible a las problemáticas que por mínimas que parezcan pueden incidir de alguna manera en el desarrollo de procesos.
- Aspectos legales como documentación agraria, padrones agrarios, reglamentos internos o estatutos comunales, actas de asamblea (acuerdos).
- Evaluación de habitantes que tienen presión sobre los recursos y el porcentaje con acceso formal a la tierra.
- Asuntos agrarios: cesión de derechos y porcentaje de personas sin derecho formal a la tierra, pero si con acceso en la práctica a ella.
- Identificar y entender las dinámicas locales particulares y los espacios de decisión colectiva (asambleas comunitarias y aspectos regionales).

ESTRATEGIA DE INTERVENCIÓN

En términos generales se sugiere la siguiente estrategia de intervención para la conformación, integración y funcionamiento de un comité de monitoreo comunitario, no podemos afirmar que este modelo sea el ideal, porque el núcleo de población en los ejidos o comunidades agrarias tienen sistemas dinámicos, es decir presentan un proceso continuo de cambio (adaptación, ajuste y reorganización), dependiendo las necesidades que se presenten en ellos.

Este planteamiento se basa en el acompañamiento y sistematización del programa piloto de monitoreo comunitario en áreas forestales que se encuentran en la región de cuencas costeras de Jalisco, el cual resumimos en el siguiente esquema:

Esquema de la estrategia de intervención

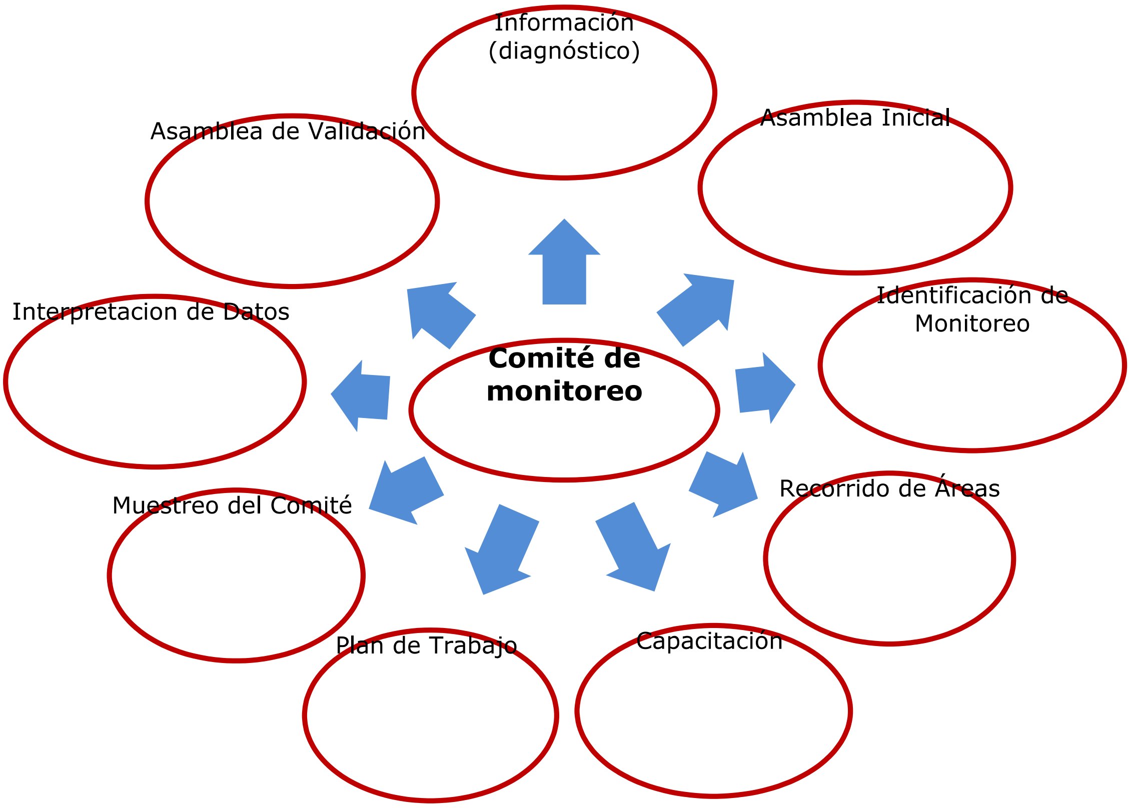

DIAGNÓSTICO LOCAL COMUNITARIO

Las herramientas y técnicas participativas para la elaboración de un diagnóstico comunitario son fundamentales porque permiten tener información de primera mano para poder identificar las necesidades locales, pero también áreas de oportunidad en las que se pueden detonar, fortalecer o acompañar procesos de desarrollo comunitario que se presentan en determinado núcleo agrario, estos pueden ser en los siguientes aspectos:

- Culturales;
- Económicos;
- Productivos;
- Ambientales; y
- Sociales.

Las herramientas participativas nos permiten identificar las circunstancias actuales de cualquier núcleo agrario y sobre todo su apropiación de cada circunstancia, porque son los actores locales quienes las viven cotidianamente.

En algunos casos, los ejidos cuentan con diagnósticos o proyectos en ejecución, sobre todo aquellos que han iniciado un proceso de desarrollo forestal comunitario, éstos pueden ser de gran utilidad para conocer la vida en el interior de la comunidad, los documentos útiles son: evaluación rural participativa, estudios de ordenamiento territorial comunitario, informes de cursos de capacitación, proyectos de inversión o productivos, entre otros.

Dadas las circunstancias de la creación de un comité de monitoreo comunitario de recursos naturales, tendrá que ser un diagnóstico más documental que de tallereo, pero vale la pena dedicarle un par de ejercicios para afinar e identificar las fortalezas y las amenazas internas y externas así como la identificación de los actores locales que intervienen en la vida del ejido o comunidad agraria, porque éstos son dinámicos porque presentan un proceso de cambio continuo.

La información que obtengamos de los ejidos o comunidades agrarias nos dirá a que nos enfrentamos y que podemos hacer para no llegar con los ojos vendados.

A veces la información no está a la mano, por ello recomendamos que hagamos uso del derecho de acceso a la información utilizando herramientas como el sistema Infomex mediante el cual podemos solicitar información pública casi siempre a un costo monetario mínimo, sin embargo, el trámite es tardado y tenemos que solicitarlo con mucho tiempo de anticipación. Prácticamente todos los estados de la república cuentan con su propio sistema local para acceder a la información pública.

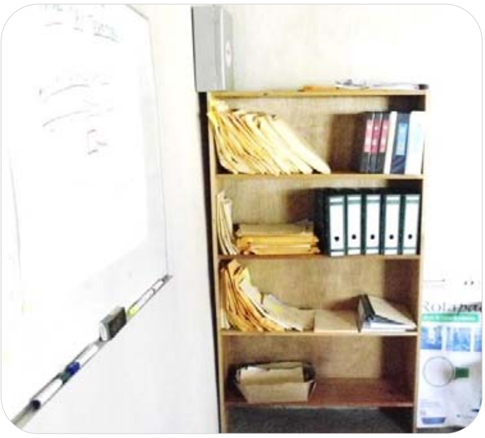

Una vez que tengamos un pre-diagnóstico del ejido o comunidad agraria, podemos acercarnos a las autoridades (Comisariado, Consejo de Vigilancia, principales o Consejo de ancianos) del núcleo agrario para platicar sobre la importancia de conformar un comité de monitoreo comunitario para el monitoreo de sus recursos naturales de uso común y concertar con estos actores una asamblea general para explicarlo a todos los ejidatarios o comuneros.

Es posible que mediante el pre-diagnóstico identifiquemos a otros actores externos como los asesores técnicos, Ayuntamientos, Juntas intermunicipales (en algunos estados), promotores de instituciones gubernamentales, visitador agrario, entre otros, a los que no debemos excluir porque son aliados estratégicos que permiten detonar procesos del desarrollo comunitario.

ASAMBLEA GENERAL INICIAL

La Ley Agraria y las propias costumbres de los ejidos o comunidades agrarias contemplan que el máximo órgano para discutir, analizar y tomar decisiones es la asamblea general porque participan todos los ejidatarios o comuneros con derechos legalmente reconocidos. La ley estipula que se pueden realizar dos tipos de asambleas: extraordinaria y ordinaria. En los núcleos agrarios, es muy probable que exista un reglamento ejidal o estatuto comunal que regula el comportamiento y funcionamiento de los mismos. Con base en este instrumento jurídico o por costumbre, los ejidatarios o comuneros se reúnen periódicamente y lo pueden hacer a finales o a principios de año, o estipulan un día de cada mes o cada dos meses o bien dos veces al año, y hay quienes sólo se reúnen cuando hay una convocatoria o citatorios personalizados, en el caso de las asambleas extraordinarias, pero también hay ejidos o comunidades que acuden a la Procuraduría Agraria a que les hagan las convocatorias además de que tiene que estar presente el visitador agrario (representante de la Procuraduría Agraria) para que se realice la asamblea. Legalmente no es necesario que asista el visitador agrario, sin embargo hay núcleos agrarios en los que si no se presenta, no tiene validez legítima la asamblea. De cada asamblea hay acuerdos que se toman y éstos deben estar asentados en actas de asamblea o bien en su libro de actas.

Tomados los acuerdos en el primer acercamiento con las autoridades locales del núcleo agrario (comisariado, consejo de vigilancia y/o principales o consejos de ancianos), se debe presentar la iniciativa explicando la importancia del monitoreo comunitario de recursos naturales, siendo pertinente preguntar cada cuándo se reúnen y cómo realizan la convocatoria, con el fin de asistir a una asamblea ordinaria o bien convocar a una extraordinaria, según sea el caso. Si ya estuvieran estipuladas sólo habría que solicitarles que se nos incluya en un punto del orden del día y así poder comunicarles la importancia del monitoreo, convencidos de ello, es apropiado solicitar que se auto-propongan y propongan a personas del ejido o comunidad para formar parte del comité y no olvidarnos que un ejidatario puede proponer a alguien más, mencionando que no tiene que ser exclusivamente ejidatario, sino que también pueden participar hijos de ejidatarios y avecindados sugiriéndoles el perfil adecuado para ello, el cual se menciona anteriormente. Vale la pena recalcar que no es necesario que todos

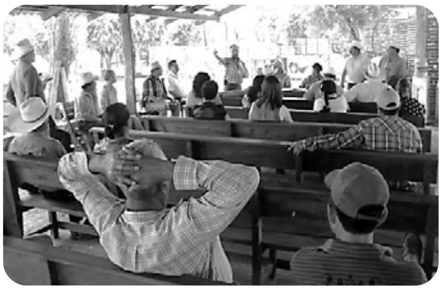

integrantes del comité de monitoreo comunitario deban saber leer, escribir y conocer operaciones matemáticas, porque generalmente son las personas adultas quienes carecen de esto pero son quienes conocen el territorio (limites, colindancias, parajes, etc.) del núcleo agrario. Sin embargo también es de vital importancia que se promueva la participación e involucramiento tanto de jóvenes como de mujeres.

La importancia de realizar esta asamblea, además de constituir el comité con auto-propuestas y propuestas de personas del núcleo agrario, es que haya un involucramiento general, por lo menos informativo, porque es la etapa inicial del proceso. En esta asamblea se puede tomar el acuerdo del inicio de las actividades para realizar el monitoreo comunitario de sus recursos naturales.

Se recomienda preparar una presentación ante la asamblea mediante la proyección de diapositivas; cuando no haya condiciones para hacerlo, se sugiere el uso de hojas de rotafolios o papelotes. Estas proyecciones o ilustraciones son herramientas que permiten atraer la atención de las personas, considerando que en las asambleas generales hay mucha concurrencia de personas y a veces es difícil tener su atención sobre todo cuando han estado mucho tiempo en ella. Se recomienda revisar bibliografía sobre técnicas de manejo de grupos e identificación de líderes comunitarios.

Una vez que se concluya el proceso y se acuerde la fecha para dar inicio con las actividades, será de vital importancia identificar, con el grupo auto-designado y/o propuesto, la definición de qué se quiere monitorear, las áreas que se quieren monitorear y hacer la priorización del mismo.

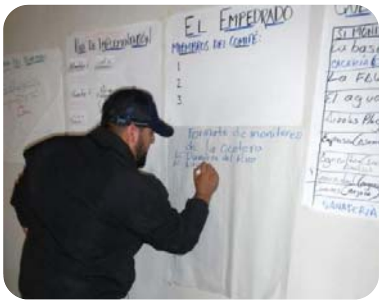

En caso de que los líderes comunitarios no se involucren en el comité de monitoreo, será de vital importancia, acercarse a ellos e invitarlos personalmente a que se incluyan en la siguiente sesión de trabajo, nunca y por ningún motivo se deben dejar fuera o de lado a estos líderes, porque son personas que saben y en momentos determinados influyen para la toma de decisiones en el ejido o comunidad agraria.

IDENTIFICACIÓN Y PRIORIZACIÓN DEL MONITOREO

Uno de los aspectos que deben considerarse en un primer acercamiento con los integrantes del comité de monitoreo comunitario, es la identificación de los actores locales, porque esto nos permitirá conocer la estructura de los mismos y de las actividades que realiza el núcleo de población en su interior. El análisis organizacional e institucional tiene como principal objetivo aprender sobre las organizaciones y grupos activos en el núcleo agrario y cómo los integrantes del comité de monitoreo comunitario los visualizan, permitiendo entender la interacción que tienen las organizaciones entre sí. Empero, pueden ayudar a determinar responsabilidades en la planificación. El uso de la herramienta diagrama de Venn, para este tipo de ejercicios es muy recomendado…

Una vez identificados los diversos actores tanto internos como externos al núcleo agrario, se recomienda realizar un mapa general del ejido o comunidad agraria para conocer cómo esta y qué recursos tienen en su territorio, en algunos casos los núcleos agrarios tienen un ordenamiento territorial comunitario, pero vale la pena analizar desde el punto de vista local, así también se determina la apropiación del estudio. Muchas veces, los jóvenes y mujeres desconocen la distribución y límites del territorio del núcleo agrario siendo este un buen momento para que vayan familiarizándose con él, en ocasiones los grupos necesitan ver el plano original y trazar el polígono para de ahí partir y plasmar lo que hay en el interior de su territorio, aquí hay que identificar y hacer énfasis en los recursos naturales de uso común porque se pretende realizar un monitoreo sobre éstos.

En el ejido Santiago de los Pinos, municipio de San Sebastián del Oeste, estado de Jalisco, con la experiencia piloto del monitoreo comunitario, determinaron monitorear la plaga (muérdago) en los pinos de su ejido y su avance; percatándose del daño que se está ocasionando. En primera instancia debían convencer a la asamblea general de ejidatarios para intervenir y solicitar apoyos a las instituciones correspondientes, así como presentar el caso a estas últimas y poder intervenir los árboles que apenas se están plagando, ya que por norma no se pueden intervenir, pero el caso es que si no se intervienen el problema seguirá, el análisis organizacional/institucional permitió fácilmente identificar a quién citar a la misma asamblea para exponer el caso.

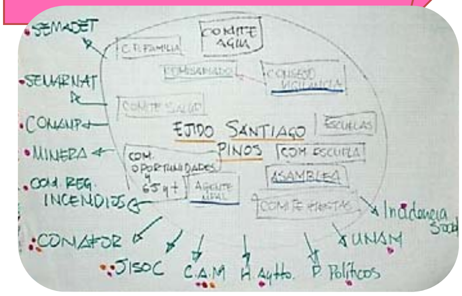

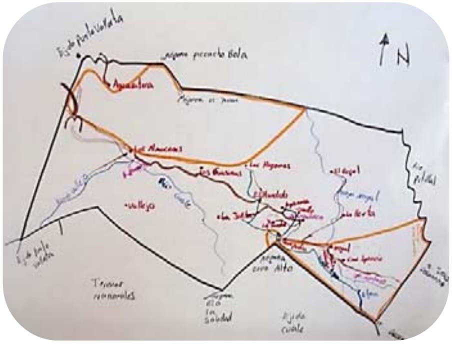

Después del proceso de ubicación de los recursos naturales de uso común en el territorio del ejido o comunidad agraria, es conveniente aterrizar para hacer las siguientes preguntas ¿qué recursos se podrían monitorear? y ¿cuáles serán los beneficios si los monitoreamos? esto debido a que todos estarán familiarizados con los recursos que hay en el territorio, refiriéndonos a que los jóvenes y mujeres que no conocían el territorio por lo menos en un plano general que el comité elaboró, ya tienen cierta noción de lo que existe y dónde están.

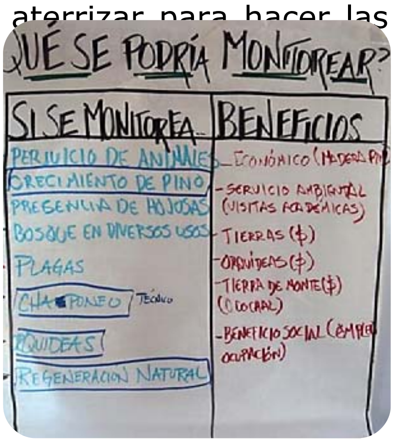

Este ejercicio se puede realizar mediante el uso de la técnica lluvia de ideas, será conveniente darle la batuta a uno de los integrantes del comité para que haga anotaciones, en una hoja de rotafolio, de los comentarios de sus compañeros o bien se le podría dar una hoja tamaño carta o media carta en blanco, acompañada de un plumón, a cada participante, y una vez que termine de llenarla, respondiendo a las preguntas generadoras, pase a pegarla en la hoja rotafolio, concluido el proceso se puede definir la importancia del monitoreo y el encargado de dirigir al grupo (facilitador) oriente el proceso a la importancia y tratar de identificar en colectivo el problema de raíz y asociarlo con los beneficios que nos traerá si lo monitoreamos. Esto porque en ocasiones se define que la disminución de agua es un problema que atañe a todos los habitantes del núcleo agrario, y sin lugar a dudas es un problema, pero lo que ocasiona esta disminución de los mantos acuíferos realmente es que no hay demasiados árboles o bien los han aprovechado sin ningún control en las partes altas, las cuales permiten la captación para una filtración de agua y se puede asociar a este entorno la pérdida del suelo y si un bosque se encuentra en mal estado (enfermo o plagado), realmente el problema es que nosotros ocupamos los árboles en las partes altas sin tener noción o falta de conciencia en este sentido.

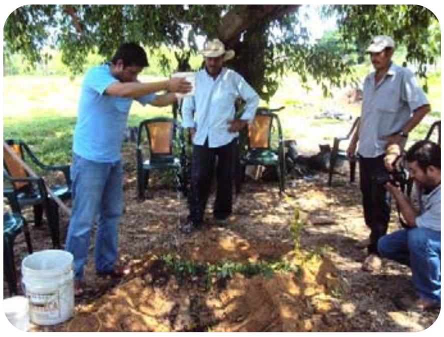

Será recomendable realizar un pequeño ejercicio con el comité para reforzar la idea del manejo de cuenca, para ello podemos recomendar el uso de una maqueta de tierra, donde simulen las montañas o cerros (preferentemente en parte aguas) y de un lado le acomodamos

muchas hojas simulando que es la parte may parte la dejamos sin hojas, posteriormente con la lluvia para analizar como es el comportamien lados, dándonos pues una idea general de l territorio.

Este ejercicio es una herramienta que puede adaptarse para poder identificar qué es lo más importante a monitorear o mejor dicho homogenizar una problemática entre el comité de monitoreo comunitario y partir de ahí, de esa problemática compartida…

Identificada la necesidad del monitoreo por los mismos actores locales (comité de monitoreo comunitario), será conveniente realizar un mapa del área que se pretende monitorear, especificando dónde están estos recursos y cómo podemos acceder al lugar (carreteras, brechas, caminos, etc.). Esto con el afán de acudir al lugar de los hechos en una visita posterior y determinar entre todos el grado de afectación y poder seguir sensibilizando y formalizando el quehacer del comité de monitoreo comunitario, tratando de prever el tiempo que nos llevará la posterior sesión de trabajo. Este ejercicio será mucho más fácil que el mapa general del núcleo agrario porque se enfocará únicamente al área de monitoreo en las zonas de uso común.

El comité de monitoreo comunitario de **El Jorullo y Anexos**, municipio de Puerto Vallarta, Jalisco, compartió la problemática del agua (disminución de los mantos acuíferos), modificando la estrategia inicial del programa piloto, sin embargo se dio seguimiento a este proceso, llevándonos a una reflexión colectiva que es de vital importancia mantener las partes altas del territorio con arbolado porque son éstos quienes permiten la captación y filtración de agua hacia los mantos acuíferos, orillándolos a realizar algunas reforestaciones. La asamblea general de ejidatarios les destinó un apoyo económico para reforestar y el comité se comprometió a presentar avances (rendir cuentas) a esta, sobre el comportamiento de la reforestación: monitoreo.

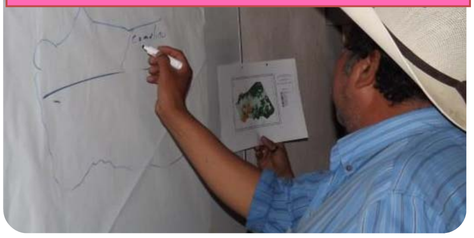

RECORRIDOS DE ÁREAS A MONITOREAR

Este recorrido que se plantea como un proceso de integración y consolidación del comité de monitoreo comunitario, es precisamente para reforzar y afianzar aún más la unificación de los mismos integrantes y sirve para reconocer la zona que se pretende monitorear, será fundamental hacer uso de la herramienta transecto o diagrama de corte una vez que se haya concluido. Sin embargo, el uso de otras herramientas como la del objetivo al inicio del recorrido permitirá reforzar el grado de organización y recalcar la importancia de comunicarse, coordinarse, organizarse y planificar los recorridos del monitoreo, previo, durante y después.

La dinámica del objetivo, utilizada con los comités de monitoreo piloto resulto en el caso del ejido Barranca del Calabozo, municipio de Pihuamo, estado de Jalisco, un éxito porque les permitió darse cuenta que aunque sea un ejido con un nivel organizativo alto en el estado, aún carecen de ciertos detalles que tienen que fortalecer en el ejido, además de aceptar que la comunicación, la coordinación y la organización son fundaméntales para que el comité y el mismo ejido funcionen adecuadamente; por otra parte, sirvió como una dinámica de animación previa al recorrido de campo, esto animó al grupo porque se divirtieron después de un rato sentados para trasladarse al lugar del recorrido e hicieron comparaciones con otros ejercicios en los que han participado.

El diagrama de corte o transecto, tiene el objetivo de propiciar discusiones y estructurar áreas dentro de la zona de influencia de la comunidad, con sus diferentes usos, problemas asociados y potencialidades de desarrollo, logrando servir también de punto de partida para la discusión de alternativas. Las preguntas generadoras de la discusión que frecuentemente se usan son: ¿qué hay en cada zona?, ¿por qué se encuentra específicamente en esa zona?, ¿quién trabaja y se beneficia de estos recursos? y ¿se han dado cambios importantes en el pasado?

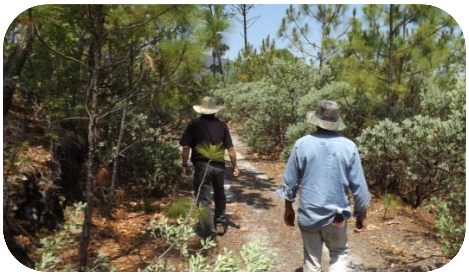

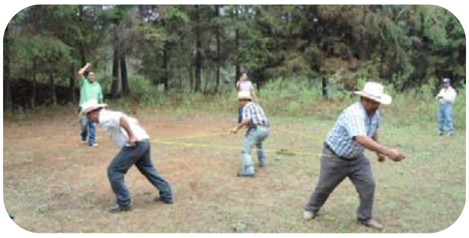

La dinámica del objetivo consiste en poner a tres personas en forma de círculo adentro de una cuerda (la cual tendrán que ponérsela en la cintura), entonces, a un metro de distancia de cada persona que se encuentra en el círculo viendo hacia afuera y a la cuenta de "tres" todos tienen que alcanzar su objetivo, sin dar explicaciones sobre lo que se pretende, si se jalonean cada quien por su lado es indispensable darles otra oportunidad, si vuelven hacer lo mismo es momento de preguntar a las personas, que no están en el círculo, ¿qué vieron? después a los que participaron

¿cómo se sintieron?, concluidas las reflexiones es necesario ahondar en la importancia de los cuatro elementos que hemos mencionado (coordinación, comunicación, organización y planificación) de acuerdo a los comentarios que hicieron los que participaron (dentro del círculo) y los observadores (fuera del círculo).

El recorrido pretende determinar el uso de los instrumentos para poder realizar tanto los inventarios como el monitoreo, dependiendo que es lo que pretendan monitorear para programar y preparar un esquema de capacitación adecuada a las necesidades del comité de monitoreo comunitario.

> La experiencia que se tuvo con el programa piloto de monitoreo comunitario se enfocó en dos cosas: agua y árboles. Como se ha mencionado, ambos nos llevan a lo mismo, pero uno es más tardado y requiere el uso de instrumentos diferentes inicialmente, esto porque no podemos utilizar los mismos instrumentos para monitorear agua y árboles. Sin embargo, la primera (agua) nos llevó a que es necesario monitorear árboles para mantener los mantos acuíferos, así que después se utilizaron los instrumentos para medir árboles y posteriormente los que monitorearon directamente árboles, es posible que después quieran monitorear el agua y tendrán que utilizar los instrumentos para medir el flujo de agua de sus mantos acuíferos.

CAPACITACIÓN

Cuando escuchamos las palabras educación o enseñanza, automáticamente las asociamos con alumnos, escuela, profesores y con nuestras propias experiencias vividas en épocas pasadas. Sin embargo, cuando nos toca enseñar o capacitar como técnico/a o promotor/a, la situación es diferente. Las y los participantes generalmente son personas adultas, con sus estilos de vivir y pensar, con sus experiencias previas, responsabilidades, motivaciones y facilidades.

La comunicación y la didáctica son las partes medulares de los procesos educativos, sin embargo solo ahondaremos en el proceso formativo de los adultos en un taller considerando que la fórmula más efectiva de enseñanza aprendizaje con estos, son: oír, ver, dialogar y hacer.

Entonces, la estrategia de capacitación propuesta debe estar basada en cuatro premisas fundamentales las cuales son: dinámico, conceptos teóricos sustentados, práctico y hacer uso del sentido común.

El proceso de capacitación implementado con los comités de monitoreo comunitario en el proyecto piloto en los ejidos de las cuencas costeras de Jalisco es el siguiente:

1. Reflexión sobre la necesidad del monitoreo.
2. Definición de conceptos.
3. Demostración de los conceptos.
4. Desarrollar la capacidad de planificar.
5. Creación e identificación de las herramientas apropiadas.
6. Práctica de campo.
7. Proceso y tratamiento de la información obtenida.
8. Análisis de los resultados.

La reflexión sobre la necesidad del monitoreo comunitario debe partir obligatoriamente de los problemas locales del ejido o comunidad agraria, nunca y por ningún motivo se debe imponer un punto de vista, ya que no se apropiarán porque no lo ven como parte de una necesidad que los pueda beneficiar, por ello todas las partes deben estar en común acuerdo; por ejemplo los técnicos, las instituciones gubernamentales y los mismos

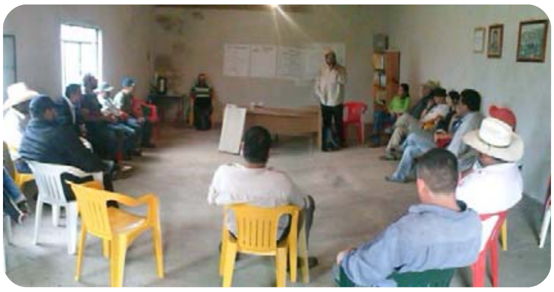

comunitarios o integrantes del comité de monitoreo comunitario deben estar de acuerdo para coincidir en las ventajas del monitoreo y ejemplificar algunos casos. Dentro de este tema se puede incluir la definición de conceptos (inventario, monitoreo y muestreo), cuya reflexión teórica va de la mano con los que se pretende realizar posteriormente, resaltando que con la información que se obtenga se adquiere cierto poder de decisión sobre lo que se hace, quiere y pretende realizar.

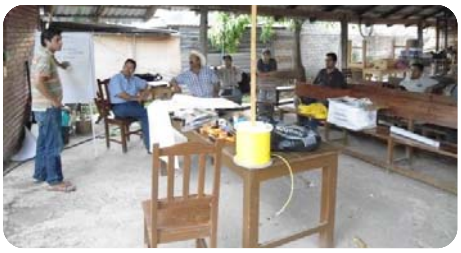

La demostración de los conceptos debe enfatizarse en la premisa de realizar un inventario o bien monitorear los recursos naturales, dejando en claro los conceptos de inventario, monitoreo y muestreo, con base en ejercicios prácticos que precisamente nos haga razonar a través de una muestra, la verdad de un asunto, por ejemplo:

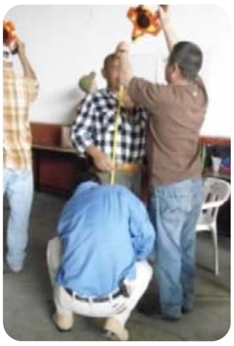

Durante el proceso de capacitación técnica que recibieron los integrantes de los comités de monitoreo comunitario de recursos naturales en los ejidos piloto de las cuencas costeras de Jalisco, se realizó un ejercicio que consistió en medir las alturas de cada uno de los participantes, con la finalidad de mostrar que se pueden obtener datos de importancia para ayudar a determinar la estatura promedio del grupo, se tuvo que medir (inventariar) las estaturas para la obtención del promedio de los participantes.

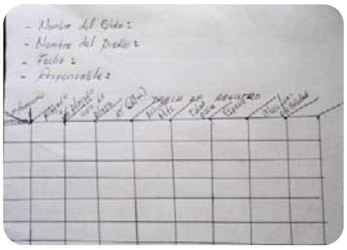

Desarrollar la capacidad para planificar de los integrantes de los comités de monitoreo comunitario de recursos naturales es la parte medular de la capacitación, porque se refuerza el interés para que sean ellos mismos quienes determinen sus necesidades con base en sus condiciones locales, ¿qué queremos saber? es la pregunta que deben hacerse a los integrantes de dichos comités para que con base en sus respuestas sean ellos quienes diseñen sus propios formatos y puedan determinar qué necesitan saber de sus recursos, para reflexionar colectivamente a base de prueba y error, es decir, en qué se falla y qué de lo hecho está bien.

Retomando la experiencia de los comités pilotos de los ejidos de Jalisco, determinaron qué querían saber: el diámetro, la altura, la edad, la especie, la plaga, el tipo de plaga, clasificación por calidad, el volumen, entre otros; en el caso particular de uno de los ejidos, determinó que le interesaba saber la cantidad y calidad de agua de sus arroyos y ojos de agua, posteriormente, con algunas reforestaciones, los llevó a considerar el diámetro, la altura y su propagación con la finalidad de rendir cuentas a la asamblea general, autorizando e invirtiendo para la ejecución del proyecto del comité de monitoreo comunitario.

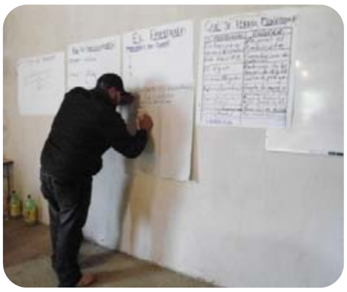

La planificación es una parte importante porque determina lo qué queremos saber de nuestros recursos, cómo tenemos que organizarnos y permite escoger el medio más apropiado para lograr lo que necesitamos, es decir, tenemos que decidir con anticipación lo que se tiene que hacer. Debemos programar (anticipar) las actividades para el levantamiento de datos en campo.

Una vez que se identifique la necesidad del monitoreo y determinado lo que queremos, se deben crear e identificar las herramientas o instrumentos apropiados, para poder realizar el inventario y monitoreo de los recursos, porque es cuando sabremos realmente qué instrumentos de medición necesitaremos…

Resaltando nuevamente la experiencia de cuencas costeras de Jalisco, donde se mostraron algunos de los instrumentos de medición como son: las cuerdas compensadas, la cinta diamétrica, la cinta métrica, el clinómetro, taladro de Pressler y el GPS (Geo-Posicionador Satelital); por otra parte, al ejido que le interesó la temática del agua se consideraron un par de pelotas plásticas que flotaran en el agua, cinta métrica, cronometro y el GPS.

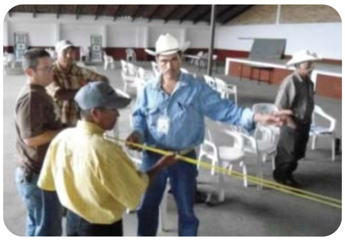

Es importante que una vez conocidos los instrumentos de medición de manera física se ponga en marcha una práctica de campo en algún lugar cercano y se pueda verificar el funcionamiento de cada uno de los instrumentos conforme a lo siguiente:

a) Delimitación del área con apoyo del GPS (Geo-Posicionador Satelital) para determinar la superficie a muestrear, lo cual nos ayudará a determinar el tamaño de la muestra e intensidad de muestreo mediante una discusión colectiva sobre las características y dimensiones de los sitios de muestreo y el tamaño de las cuerdas compensadas pueden determinar, los integrantes del comité de monitoreo comunitario, el número de sitios de la superficie para realizar el muestreo.

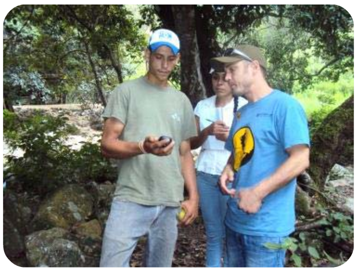

Con base en su mapa y conocimiento del territorio, facilitará la ubicación y localización de los sitios, porque podemos encontrar áreas accidentadas que impiden realizar y establecer los sitios de muestreo en esos lugares, pero también ubican los lugares donde hay más claros (áreas con menor cobertura de árboles) y en donde hay mayor cobertura o se intensifica el arbolado. Por ello se debe considerar el establecimiento de sitios en ambas áreas, ya que eso homogeniza y nos acerca a una realidad de lo que tenemos en el bosque.

La cuerda compensada determina el tamaño del sitio compensando las pendientes según el terreno donde se establecen los sitios.

b) La determinación del diámetro normal o grosor del tronco y la altura de los árboles, en los sitios establecidos previamente con la cuerda compensada, tendrá que hacerse con el uso de la cinta diamétrica y el clinómetro, respectivamente.

Si queremos conocer el bosque se deberán tomar algunas mediciones y así podremos tomar mejores decisiones para aprovecharlo mejor.

El diámetro de los árboles nos da una idea de su crecimiento, además de que es mucho más fácil medir su diámetro que su altura, ya que sólo se toma el dato a una altura promedio de 1 metro y 30 centímetros o bien podemos estandarizarla a la altura del pecho (Diámetro a la Altura del Pecho DAP), esta medida nos permitirá conocer el área basal.

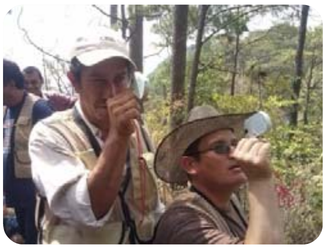

El clinómetro sirve básicamente para medir la altura de los árboles en cuyo caso podemos determinar cuánto crecen hacia arriba o a lo alto. Con el uso de estos dos instrumentos podemos determinar cuánto crecen a lo ancho y a lo alto, medidas que son fundamentales para determinar el volumen.

La demostración del muestreo debe hacerse con base en los datos que se recolecten junto con los integrantes del comité de monitoreo comunitario, porque se pretende demostrar los resultados del muestreo desarrollando una tabla donde se plasme el número de árboles por hectárea y total de plantas, el incremento medio anual en altura y diámetro normal, la sobrevivencia de las plantas, volumen por hectárea y total de superficie muestreada.

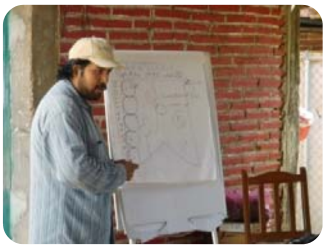

El análisis de los datos debe hacerse a los ojos de todos los integrantes del comité de monitoreo comunitario, de tal forma que vayamos entendiendo y reflexionando en colectivo, si hay dudas se debe enfatizar en el punto aunque lo repitamos varias veces, si es necesario. Debe ser convincente el análisis ya que la información que resulte seguirá motivando a los integrantes del comité de monitoreo comunitario, sobre todo manejar datos para presentarlos en asamblea y determinar cualquier acción al respecto dependiendo de lo que interese o pudiera interesar a los ejidatarios o comuneros en las asambleas generales.

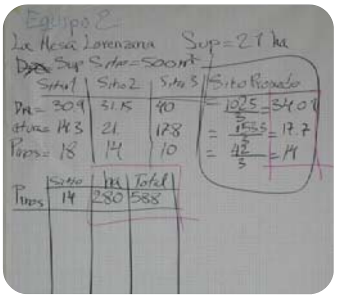

Al ejido Santiago de los Pinos, municipio de San Sebastián del Oeste, Jalisco, como ejido piloto, le interesó el tema para monitorear la plaga (muérdago), que está consumiendo poco a poco su bosque, además de que hay un avance significativo en su propagación; en asamblea general se determinó tomar las medidas necesarias para combatirlo y manejar el bosque.

Por otra parte, al ejido Barranca del Calabozo, municipio de Pihuamo, Jalisco, le interesó el monitoreo de su aprovechamiento en los rodales y cómo está en la búsqueda de su certificación internacional, tienen como una de sus obligaciones, establecer sitios de muestreo, pueden también determinar el volumen, la

clasificación de la madera y buscar compradores previamente antes de iniciar con el aprovechamiento.

Asimismo, el ejido el Jorullo y sus Anexos, municipio de Puerto Vallarta, Jalisco, por la escasez del agua decidieron monitorear este tema, porque tiende a disminuir considerablemente en estiaje cuyas decisiones son aprobadas en asamblea, las propuestas de reforestación y obras de conservación de suelo y agua son presentadas por el comité de monitoreo a la asamblea ya que aportó recursos económicos para realizar plantaciones pilotos para que el comité monitoree su crecimiento, propagación y determinar si ayuda o no a retener y aumentar el agua en parcelas del ejido.

Posteriormente se debe poner a prueba la asimilación de lo aprendido por los integrantes del comité de monitoreo comunitario con un ejercicio práctico donde sean ellos quienes pongan en marcha todo lo aprendido, y se debe acompañar sin involucrarse demasiado en la práctica porque la idea es que se den cuenta de las debilidades que tienen, al concluir esta práctica se puedan abordar o bien despejar dudas, recordando que es un proceso de aprendizaje de prueba y error. Esta práctica debe hacerse repasando todo lo que se ha visto desde la planeación, cuyos puntos centrales son:

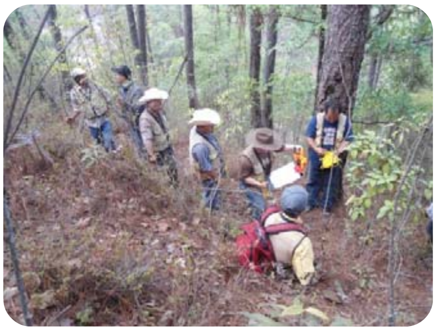

Identificación del área
Formato de campo
Delimitar el área
Tamaño de sitios
Intensidad de muestreo
Secuencia de cálculo

Otro de los aspectos que se debe abordar es la cuestión del sistemas de información geográfica, aunque pareciera complicado el uso de estos programas, es indispensable dar a conocer a los integrantes del comité de monitoreo comunitario el funcionamiento y utilidad de éstos. Los integrantes de los comités de monitoreo comunitario pilotos de cuencas costeras de Jalisco conocieron, exploraron y presentaron a sus asambleas mapas elaborados en estos programas, para su elaboración y diseño se involucraron prácticamente los jóvenes que manejan computadoras a quienes se les facilitó el uso del SIG, sin duda, a los mayores les pareció complicado pero entendieron su importancia.

PLAN DE TRABAJO COMUNITARIO

Se recomienda que una vez que se los integrantes del comité de monitoreo comunitario hayan conocido el uso de instrumentos de medición, estando capacitados para que por sí mismos puedan realizar un muestreo de sus recursos naturales, se haga un plan de trabajo con ellos, donde una de las actividades que se enmarque en él, sea la de establecer formalmente el área de monitoreo, esto con la finalidad de volver a repasar algunas cosas donde seguramente saldrán dudas.

El plan de trabajo necesita ser elaborado colectivamente con base a las capacidades del grupo o comité de monitoreo comunitario, este plan de trabajo puede elaborarse dando respuesta a las siguientes preguntas:

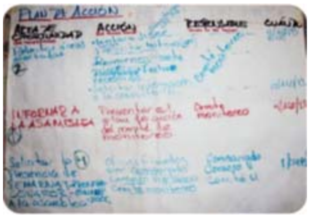

- ¿Qué necesitamos?
- ¿Qué vamos hacer?
- ¿Quién nos puede apoyar?
- ¿Quién lo va hacer?
- ¿Cuándo lo vamos hacer?

En la experiencia de los comités de monitoreo piloto de las cuencas costeras de Jalisco, estos planes de trabajo estuvieron enfocados a realizar actividades de monitoreo de los recursos naturales dependiendo sus problemas locales (Santiago de los Pinos –plaga-, Barranca del Calabozo –rodales-, El Jorullo –agua-). En el caso particular del comité del ejido el Jorullo y debido a la falta de información se hizo hincapié en realizar actividades que contribuyen a fortalecer el tejido social encaminado al fortalecimiento del capital social, además de acciones concretas con el monitoreo del agua.

Una de las actividades más sobresalientes que validarán las asambleas generales sobre el funcionamiento de estos comités es la rendición de cuentas tanto económicas como de actividades del comité de monitoreo comunitario, dando confianza a ambas partes de lo que se está haciendo.

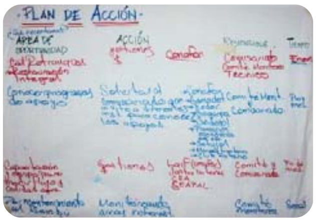

Algunos de los comités incursionaron en la solicitud de apoyos para fortalecer el trabajo de monitoreo, atreviéndose a plasmar en su plan de trabajo lo que necesitan, dónde y cuándo lo realizarán; para ello necesitan la validación de la asamblea general porque se

solicitarán a través del ejido a la Comisión Nacional Forestal y otras dependencias.

Para la construcción colectiva de este plan de trabajo y acciones del comité de monitoreo comunitario no se puede puntualizar debido a que cada comité de monitoreo tiene diferentes necesidades, pero éste se debe construir colectivamente donde se deben considerarse los tiempos (dinámica) de los integrantes de los comités de monitoreo comunitario.

MUESTREO COMUNITARIO

Establecida la actividad del muestreo sobre los recursos naturales de su ejido o comunidad en el plan de trabajo comunitario es indispensable que sean ellos mismos quienes lo realicen de acuerdo con lo aprendido durante la etapa de capacitación y práctica de campo, con el acompañamiento de los técnicos o asesores. Con esto se pretende que pongan en marcha el aprendizaje obtenido. En el proceso resultarán dudas, pero esa es la idea, para que durante el acompañamiento puedan despejarse. Recordemos que "para aprender debemos caer y levantarnos para no volver a cometer el mismo error", además de que la premisa de la capacitación plasmada en los apartados anteriores debe realizarse a base de la prueba y el error enfatizando que sí nos equivocamos no debemos retroceder sino todo lo contrario.

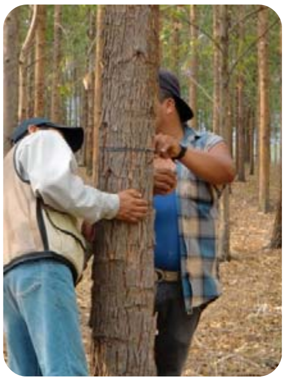

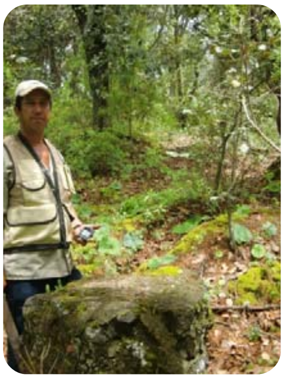

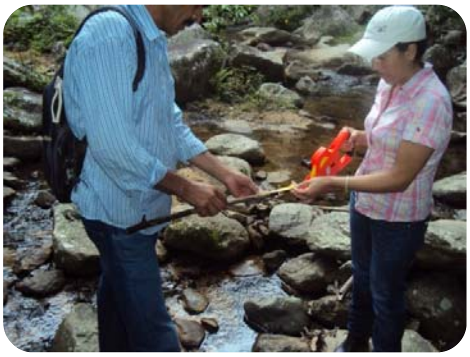

INTERPRETACIÓN DE DATOS

Antes de iniciar con la interpretación de datos, con el acompañamiento del asesor técnico, será indispensable hacer varias preguntas como: qué es lo que conocen de lo monitoreado, porque el comité de monitoreo comunitario del ejido Santiago de los Pinos con su problemática de la plaga detallaron el comportamiento de la misma, a sabiendas de la normatividad ambiental para el combate y control de plagas que no permite intervenirla desde la etapa inicial, el comité consideró que es fundamental atacar el problema de raíz.

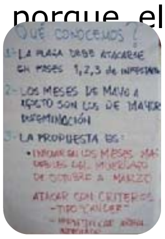

Incluso se incursionó en detectar el ciclo de vida de la plaga para determinar cuál es la época del año en la que se pudiera intervenir para erradicarla, vale la pena resaltar que han hecho esfuerzos, con apoyo de programas de gobierno, para combatirla sin embargo, no existe a la fecha un remedio que acabe con esta plaga, al aplicarla solo la tranquiliza pero pasa su efecto y revive. Con apoyo de un ejercicio que conocemos como calendario estacional, los mismos integrantes del comité de monitoreo determinaron las fechas en las que esta plaga está débil, pudiendo ser la época adecuada para combatirla.

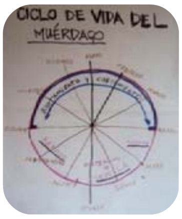

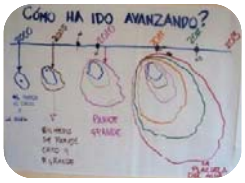

Después de identificar el ciclo de vida del muérdago se analizó el avance de hace diez años a la fecha, cuyo avance se tomó con apoyo del GPS, percatándose de que no hay efecto de los líquidos que se utilizaron para que supuestamente se erradicara esta problemática, percatándose que la plaga ya invadió otro ejido vecino.

La interpretación de los datos recolectados es sin duda una de las etapas más complicadas porque se trata de hacer cuentas, sin embargo a la hora de usar números, tenemos que traducirlos a su vida cotidiana, por ejemplo: el dinero, si hacemos cuentas suponiendo que los números son dinero todos podrán asimilarlo mejor porque aunque haya personas que no sepan leer y escribir son buenos manejando el dinero; esto sólo es un ejemplo de lo que se podría usar, por lo que debemos poner especial atención con que se relacionan más y utilizar esas formas para enseñar, es decir adaptarnos a sus formas de vida.

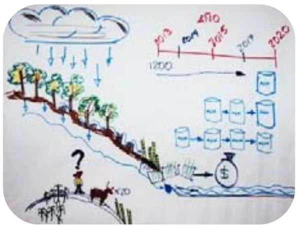

Por otra parte, en otro de los ejidos se hizo un ejercicio de manejo de números en el cual se utilizaron como ejemplos al ganado y litros agua, cuyas ejemplificaciones quedaron claras porque también se utilizaron dibujos para ejemplificar.

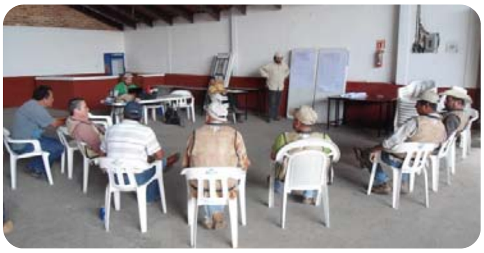

Esta etapa del análisis de los datos será de vital importancia acompañarla y solo intervenir cuando el comité de monitoreo así lo vea conveniente, ya que se trata además de propiciar que sean ellos quienes lo realicen, se valgan por sí mismos.

El análisis de datos de los integrantes del comité de monitoreo comunitario debe hacerse cada vez que se hagan los recorridos de campo para el monitoreo y de preferencia presentarlos en la asamblea.

ASAMBLEA GENERAL DE VALIDACIÓN SOCIAL

La propuesta de realizar una asamblea general en el ejido o comunidad agraria, al concluir este proceso, es con la finalidad de dar a conocer las actividades que el comité de monitoreo comunitario de recursos naturales ha realizado en el transcurso de la integración, consolidación y formación de sus integrantes, donde será de vital importancia destacar las actividades del plan de trabajo el cual debe incluir temas como: las actividades a realizar, cada cuándo se realizarán, los beneficios que se obtendrán, informar a la asamblea general tanto de las actividades como del manejo de recursos, si los hubiera. Esta información deberá servir a los ejidatarios o comuneros para la toma de decisiones para el manejo de sus recursos naturales.

Volviendo a los casos del programa piloto de monitoreo comunitario de recursos naturales en la región de cuencas costeras de Jalisco. La asamblea general de validación social de los trabajos pilotos consumados, con la exposición presentada por los integrantes del comité de monitoreo comunitario, analizaron, discutieron y tomaron acuerdos para realizar una adecuada intervención al problema de su bosque, la plaga (muérdago).

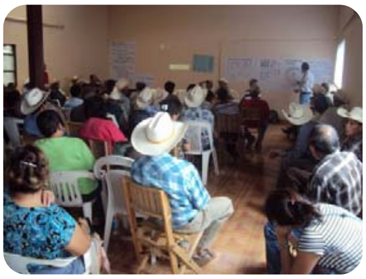

Los integrantes del comité de monitoreo comunitario de recursos naturales del ejido Barranca del Calabozo realizó su exposición ante la asamblea general a quienes les pareció importante monitorear sus recursos del bosque, sin embargo, les fue de mayor importancia este tema debido al proceso de certificación internacional por el cual están pasando.

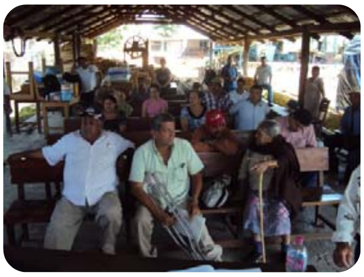

En el ejido el Jorullo, la asamblea general le vio cierta importancia al monitoreo comunitario, sin embargo, cuando los ejidatarios notaron la importancia y seriedad al proceso por los integrantes y miembros del comité de monitoreo comunitario de recursos naturales, decidieron invertir recursos económicos para realizar reforestaciones en unas áreas del ejido, cuyo monitoreo del crecimiento de las plantas como del flujo y calidad de agua

corresponde al comité para que cada dos meses se presenten los avances en las asambleas generales.

La importancia de realizar las asambleas generales es precisamente para que de una u otra forma los ejidatarios o por lo menos la mayoría estén enterados (informados) de las actividades del comité y sobre todo que la información que recaben estos, sirvan para discutir, analizar y tomar decisiones sobre el manejo de sus recursos naturales.

REFLEXIONES FINALES

La guía participativa para la integración, consolidación y formación de un comité de monitoreo de recursos naturales es precisamente que sirva para orientar un proceso participativo e incluyente en el que se analicen, discutan y tomen acuerdos del manejo de los recursos naturales a nivel núcleo agrario; las asambleas generales de acuerdo con la información que recaben los integrantes de estos comités, pueda determinar informadamente el aprovechamiento de sus recursos naturales.

Aunque este proceso piloto se inició en la región de las cuencas costeras del estado de Jalisco, puede ser replicable en otros ejidos y comunidades agrarias del país, porque es un proceso de vital importancia para el monitoreo de cualquier recurso natural que se encuentre dentro del territorio de cualquier núcleo agrario, puede ser fructífero siempre y cuando se realice a partir de las necesidades locales, siendo los propios actores locales quienes decidan qué recurso monitorear. Debemos considerar algunos aspectos como la historia, las estructuras de gobernanza local, la participación de la mujer, los jóvenes y personas que no tienen derechos agrarios en la vida del ejido o comunidad agraria.

El respeto a las autoridades comunitarias e invitación a los líderes naturales del ejido o comunidad y en algunos casos el convencimiento de consejo de ancianos o principales (personas de respeto) son de suma importancia porque así como pueden facilitar el proceso también pueden truncarlo. Es importante considerar a todos los actores externos (técnicos, asesores, promotores, instituciones gubernamentales, entre otros) con el fin de involucrarlos y tomarlos en cuenta porque en caso de no hacerlo puede ocasionar rupturas internas que no sólo trunquen o frenen el proceso, sino que puedan fracturar el tejido social comunitario del ejido o comunidad agraria.

Dar a conocer el marco jurídico que promueve la participación social y ciudadana en el tema de vigilancia y monitoreo de los recursos naturales es como un refuerzo que puede motivar a los ejidatarios para participar en lo que pretendemos, que sean los propios actores locales quienes tomen en sus manos el desarrollo de su ejido o comunidad agraria.

Afianzar este proceso a algún otro que estén desarrollando en el ejido o comunidad agraria facilitará y no duplicará procesos que tienen

objetivos similares, como es el caso de los comités de vigilancia ambiental participativa.
En cuanto a la capacitación, es de vital importancia considerar la implementación de técnicas de educación popular y propiciar el uso de los instrumentos de medición con un lenguaje entendible para que los integrantes de los comités de monitoreo comunitario puedan fácilmente hacer uso de ellos, además de dejar que hagan un monitoreo por ellos mismos, para que verifiquen cómo se usan. También se deben dejar muy claras las definiciones de muestreo, monitoreo e inventario. El involucramiento de mujeres como de jóvenes en estos comités pueden ser actividades que permitan y faciliten el manejo de instrumentos de medición, localización y recursos económicos y materiales que puedan tener a su cargo así como motivacionales para la inclusión de más personas en este tipo de proceso.

La estrategia de intervención que aquí se presenta no quiere decir que forzosamente así tiene que ser, pues es un ejemplo, pero las capacidades y conocimientos de los asesores técnicos podrán adecuar este modelo a conveniencia, siempre y cuando no se salgan del objetivo del proceso que aquí se presenta y esperamos que la experiencia que se comparte a otros, les sirva y sea de utilidad.

La validación del proceso de monitoreo comunitario de recursos naturales, afianzará la labor, que hasta el momento se considera honorifica, de los integrantes de los comités de monitoreo comunitario de recursos naturales además de permitir a la máxima autoridad de los núcleos agrarios, la asamblea general, tomar decisiones sobre el rumbo del desarrollo de su ejido o comunidad agraria.

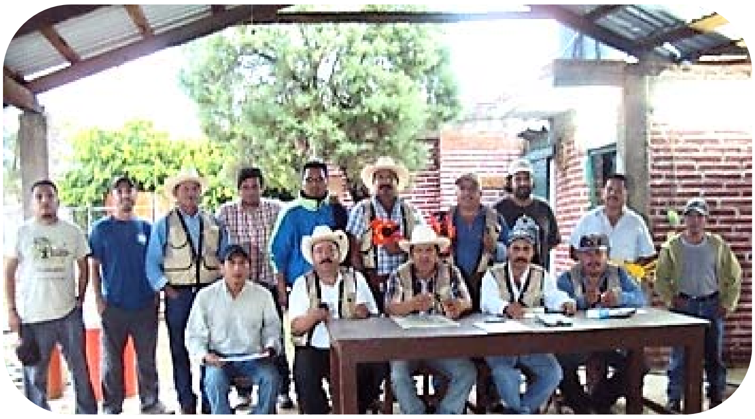

RECOMENDACIONES BIBLIOGRÁFICAS Y LEGISLATIVAS

- **GEILFUS** Frans (1997), "80 herramientas para el desarrollo participativo: diagnostico, planificación, monitoreo, evaluación"; SAGARPA, INCA Rural, e IICA, México, 2002.
- **O´HARA** Peter (2010), "Mejorando la participación de las partes interesadas en los programas forestales nacionales, Manual de capacitación" Organización de las Naciones Unidas para la Agricultura y Alimentación para los programas forestales nacionales (FAO), Roma.
- **YMCA** Mundial (2006), "Capacitación de mujeres jóvenes para liderar el cambio, manual de capacitación" Fondo de Población de las Naciones Unidas (FNUAP).
- **BAUTISTA** José y **AGUILAR** Elena, Coord., "Manual de acceso a la información, transparencia y rendición de cuentas para el fortalecimiento de las organizaciones civiles"; Instituto Federal de Acceso a la Información Pública (IFAI), octubre de 2005.
- Escuela Agraria de Oaxaca, Tequio Jurídico, A.C. y Comisión Nacional para el Desarrollo de los Pueblos Indígenas (2008). "Manual del comisariado", Escuela Agraria de Oaxaca; Tequio Jurídico, A.C., y Comisión Nacional Forestal. 2012.
- **GOMEZJARA,** Francisco (1977) "Técnicas de desarrollo comunitario", Distribuciones Fontamara, S.A. México. 1989.
- **VARGAS** Guillén, Adalberto; **ÁLVAREZ** Pérez, Micaela; **CUESTA**, Irene. "Guía Didáctica para la Participación Local en programas de Servicios Ambientales". Ed. Fray Bartolomé de las Casas A.C.
- **FAO – PESA – SAGARPA** (2007), "Metodología para fortalecer la equidad y la inclusión de grupos prioritarios", México.
- **EXPÓSITO** Verdejo Miguel, **GRUNDMANN** Gesa, **QUEZADA** Luís, **VALDEZ** Luisa; "Preparación y ejecución de talleres de capacitación, una guía práctica"; Proyecto, Comunicación y Didáctica (Centro Poveda), Republica Dominicana.
- **CÓRDOVA** Sofía y **CHAN** Ángel (Comp.), Parte y comparte, una experiencia para crecer, "Juegos y dinámicas para el trabajo grupal", Servicios de la Juventud A.C. y, Educación y Ciudadanía, A.C.
- **PROCURADURÍA FEDERAL DE PROTECCIÓN AL AMBIENTE**, "Manual de comités de vigilancia ambiental participativa", Subprocuraduría de Recursos Naturales.
- "Manual de campo: planeación comunitaria participativa" FAO- PESA-SAGARPA, México, 2007
- "Manual de técnicas participativas", estudio de validación del desarrollo rural participativo basado en la conservación de suelo y

aguas (Proyecto JALDA); Agencia de Recursos Verdes del Japón, Prefectura del Departamento de Chuquisaca, Sucre - Bolivia

- Constitución Política de los Estados Unidos Mexicanos.
- Ley Agraria.
- Ley General de Equilibrio Ecológico y Protección al Ambiente.
- Reglamento de la Ley General de Equilibrio Ecológico y Protección al Ambiente.
- Ley General de Desarrollo Forestal Sustentable y su reglamento.
- Ley General de Cambio Climático.
- Ley de Transparencia y Acceso a la Información Pública Gubernamental.
- Ley General de Vida Silvestre y su reglamento.
